# Molecular Docking and Molecular Dynamics Studies: Evaluation of the Potential Interaction between Ciprofloxacin-DAB-PAMAM Dendrimer-Based Prodrug Prototypes and Human Carboxylesterase 1

**DOI:** 10.1021/acsomega.6c04630

**Published:** 2026-10-06

**Authors:** Sophia Sueyoshi, Lucas B. Raimo, Lucas P. Coutinho, Marcos O. O. Brito, Jeanine Giarolla, Lucas C. Ducati

**Affiliations:** † Department of Pharmacy, School of Pharmaceutical Sciences, 28133 University of São Paulo, Av. Prof. Lineu Prestes, 580, São Paulo, São Paulo 05508-900, Brazil; ‡ Department of Fundamental Chemistry, Institute of Chemistry, 28133 University of São Paulo, Av. Prof. Lineu Prestes, 748, São Paulo, São Paulo 05508-000, Brazil

## Abstract

Ciprofloxacin is widely used as a drug for broad-spectrum treatment of infections. However, it is classified as class IV by the Biopharmaceutics Classification System, being characterized by low solubility and permeability. Aiming to contribute to the rational design that may improve the biopharmaceutical properties to increase drug efficacy, two series of prototypical compounds were designed: (1) ciprofloxacin prodrugs and (2) ciprofloxacin dendrimer prodrugs. Thus, this study performed molecular docking and molecular dynamics (MD) simulations to evaluate the interaction between the designed ligands and human carboxylesterase 1 (2H7C), focusing on comparing the results from both series of selected ligands. Additionally, nonspecific esterase was chosen due to its ability to cleave ester bonds *in vivo*, releasing the bioactive compound. The molecular docking results pointed out that the distance between the carbonyl group and Ser221 was shorter for Compounds 1 (dock 19: 2.2 Å) and 4 (dock 12: 1.8 Å). The MD simulations produced reasonable receptor-ligand complex interactions, which can be observed from the RMSD analysis showing significantly different trajectories with average atomic positions varying from 1.6 to 2.5 Å. Through the MD trajectories, the Potential Energy of Interaction (PEI) average values, ranging from −281.6 kJ/mol up to −1061.9 kJ/mol, showed strong interaction between the ciprofloxacin ligands and the Ser221 active site together with the MM/PBSA results. The findings indicated that the larger prodrug generations increased general receptor-ligand interaction and also specific ligand-SER interaction; thus, these findings demonstrate the potential of ciprofloxacin nanocompounds as a rationally designed treatment for infectious diseases.

## Graphical abstract

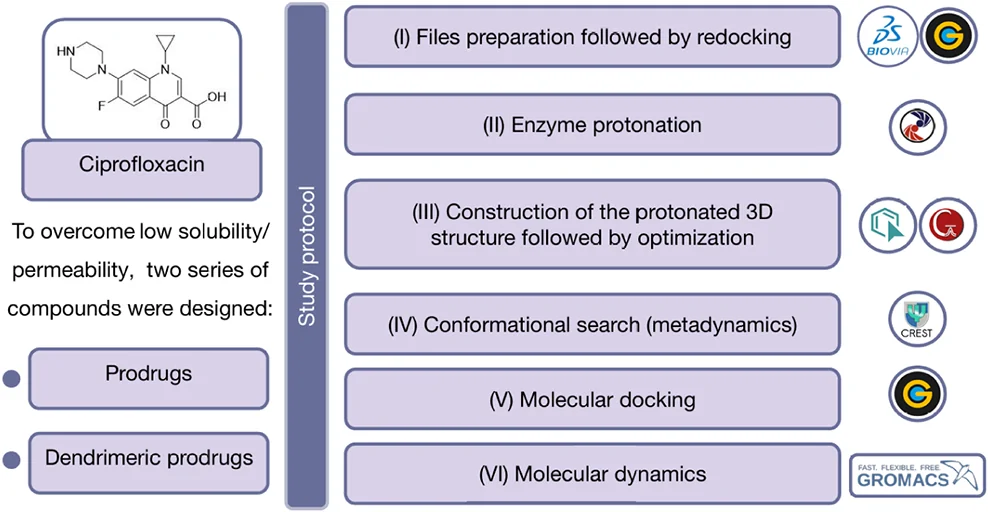

## 1 Introduction

Ciprofloxacin is an antibacterial drug used to treat infections caused by sensitive microorganisms. Its mechanism of action is based on the blockage of the DNA gyrase’s subunit A, interrupting DNA replication. In addition, this drug is considered to be of broad-spectrum and can be used against several gram-positive and gram-negative bacteria.^1,2^ It is also characterized by its low solubility and permeability, classified as class IV by the Biopharmaceutics Classification System. This zwitterionic molecule can suffer a change of behavior inside the organism, which causes variations according to the physicochemical and physiological exposed conditions. Thus, the improvement of oral bioavailability is still a challenge to overcome, and it represents one of the bigger issues related to drug discovery.^1,2^ Particularly, permeability (the capacity of the substance to overcome a barrier or membrane) and solubility (the ability to dissolve and form a solution) are important factors to be observed regarding the drug absorption processes. Thus, low solubility represents an important challenge since it can lead to poor data generation in preclinical and clinical development studies. Moreover, low permeability constitutes a critical optimization parameter, since it compromises drug absorption and bioavailability.^3,4^

Although ciprofloxacin remains one of the most widely used fluoroquinolones, the optimization of its pharmacokinetic and biopharmaceutical profile continues to attract considerable interest. Strategies capable of modulating drug distribution, controlling its release, and improving exposure at the target site may contribute to enhancing therapeutic performance. In this context, the design of macromolecular conjugates has emerged as a promising approach for the development of novel ciprofloxacin-based delivery systems.^5–7^ One possible approach to improve the biopharmaceutical properties of a bioactive compound is to use a modified diaminobutane-polyamidoamine (DAB-PAMAM) dendrimer as a drug carrier. Dendrimer differs from traditional polymers due to its unique structure formed by a central core and peripheral branches that define size, shape, and multiplicity. Thus, these nanoscale structures can adsorb an active component, in addition to covalently coupling with the molecule, resulting in the dendrimer prodrug.^8,9^

Among the available dendrimeric systems, DAB-PAMAM was selected for the present investigation. It was first described by Ficker et al., and its main modification was replacing the ethylenediamine core with a butanediamine one, increasing the reaction yield and its purity. It is worth highlighting that the high degree of synthetic control increases the structural versatility and makes DAB-PAMAM promising compared to the traditional linear polymers as drug and gene carriers.^10^ Considering the therapeutic relevance of ciprofloxacin and the need for novel approaches to optimize its biopharmaceutical performance, dendrimer conjugation represents a promising strategy for the development of potential prodrug prototypes.^11–13^ The presence of functional groups suitable for conjugation allows the design of macromolecular systems capable of modifying drug distribution and delivery.^14–16^ Consequently, understanding the interaction of these conjugates with enzymes potentially involved in their activation becomes an important step during the early stages of development.^6,17,18^

In this context, computational approaches can provide valuable information regarding enzyme-ligand recognition before experimental evaluation. The drug design process utilizes knowledge derived from the molecular basis of diseases to guide the development of new compounds, while also enabling the rational planning of structural modifications to improve existing molecules.^19^ When the molecular interactions between a ligand and its biological target are well characterized, the likelihood of generating more selective drug candidates increases, thereby enhancing therapeutic efficacy and minimizing potential adverse effects.^20,21^

In cases when the three-dimensional structure of the esterase is known and defined, and aiming to improve the cost and time of the drug development process, the computer-aided drug design (CADD) was developed. Molecular docking simulation, for instance, can predict the ligand conformation and positions inside the active site, evaluating the conformational accuracy of generated conformers according to a score function generated by the affinity energy values calculations.^22–24^ Complementary to the docking, the molecular dynamics (MD) method allows a more precise comprehension regarding the interaction between the ligand and the biological target since time evolution trajectories are provided, describing the molecular arrangements considering an explicit solvent model.^22,25–27^

Given the importance of enzyme-mediated activation in prodrug development, human carboxylesterase 1 (PDB 2H7C) was selected as an exploratory enzymatic model for this study. It is a serine hydrolase responsible for the hydrolysis of a broad range of ester-containing compounds, including prodrugs, and widely expressed in human tissues. This enzyme possesses a catalytic triad capable of nucleophilic attack on carbonyl-containing substrates, making it a suitable model for evaluating whether the designed conjugates can adopt catalytically relevant orientations within the active site of the enzyme.^28–30^

In addition, this enzyme exhibits broad substrate recognition, therefore presenting as a suitable exploratory model for evaluating the active-site accommodation of structurally complex prodrug candidates. Because the present work aims to evaluate molecular recognition rather than the catalytic reaction itself, human carboxylesterase 1 was selected as a biologically relevant model to investigate whether the proposed prodrugs are capable of adopting productive binding modes within the catalytic pocket.^31,32^ Although the designed conjugates contain an amide linkage, which is generally more resistant to hydrolysis than ester bonds, human carboxylesterase 1 was selected as an exploratory enzymatic model since mammalian carboxylesterases have been reported to hydrolyze a broad range of carbonyl-containing substrates, including certain amide-containing compounds.^31,32^

Considering all of the aforementioned data, the aim of this work was to investigate the receptor-ligand complexes formed between two ciprofloxacin prodrug prototypes and four ciprofloxacin-DAB-PAMAM dendrimer-based prodrug prototypes with human carboxylesterase 1, to evaluate their structural compatibility and their potential interaction with the enzyme’s catalytic machinery. The docking and molecular dynamics simulations were performed to evaluate whether the amide carbonyl could adopt a catalytically relevant orientation relative to Ser221, which is considered a structural requirement for productive interactions with serine hydrolases. Thus, those techniques were employed to elucidate the conformational stability of the complexes. Understanding their molecular recognition by carboxylesterases is an important step during the early stages of rational design, and can provide structural insights to support the rational design and optimization of prodrugs for therapeutic use in humans.

## 2 Computational Methods

To investigate structural features associated with potential esterase-mediated hydrolysis, computational studies were performed by placing the ligands within the protein active site to simulate key ligand-receptor interactions in an explicit-solvent environment. The resulting interaction data were analyzed to evaluate the accommodation of the designed prodrug prototypes within the catalytic site of human carboxylesterase 1 and their potential susceptibility to esterase-mediated hydrolysis.

### 2.1 Compound Selection

The ciprofloxacin prodrugs were designed using the strategies: (**i**) two prodrugs where the ciprofloxacin was connected directly to the butanediamine central focus; (**ii**) the dendrimer prodrugs, where the modified PAMAM dendrimer generation 0 (G0) has ciprofloxacin attached to its surface (Table 1).

**Table 1:**
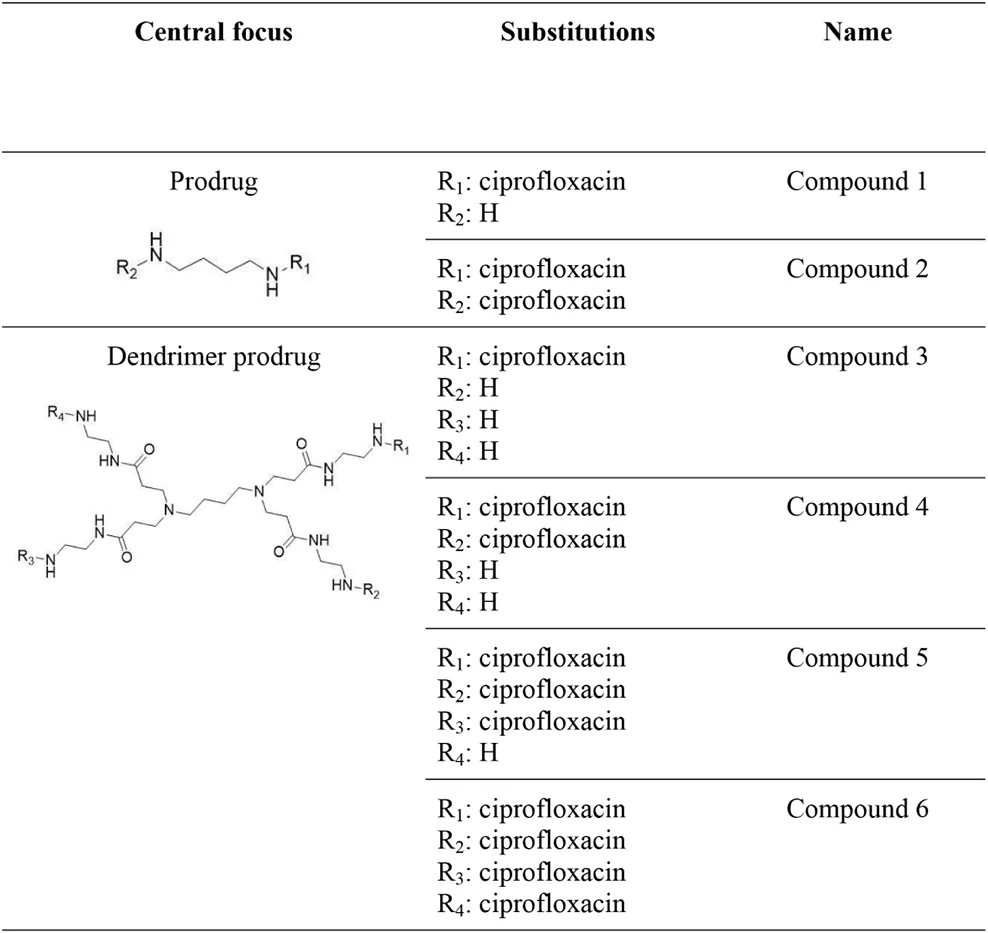
A Series of Prodrug Prototypes Containing Ciprofloxacin Applied in This Study

### 2.2 Protein Selection

The protein was selected among the options available at the Protein Data Bank, according to the resolution, robustness of the crystallography experimental data provided, and the report generated by the PROCHECK software.^33^ The protein chosen was a nonspecific esterase identified under the code 2H7C in the data bank.^28^ This enzyme is classified as human carboxylesterase 1 and is involved in the metabolism of a considerable number of substances, being extensively studied during the last decade. The 2H7C was selected as the most promising to evaluate the potential structural compatibility with the catalytic site through the prodrug candidates for having a multisite promiscuity and significant expression in the organism.^28,29^

One of the monomers was selected, and the remaining monomers were removed from the original crystal structure as well as the artifacts generated by the crystallization process; water molecules located closer than 4.0 Å were maintained since they could be involved with the mechanism of catalysis. All this procedure was made using the Discovery Studio 2019 software.^34^

### 2.3 Protein and Ligand Optimizations

The construction of the compounds 1–6 was made through GaussView software using the protonation state information generated by MarvinSketch, considering pH 7.4.^35,36^ The geometry optimization was conducted with Kohn-Sham Density Functional B3LYP, and 6–31+G­(d,p) basis set.^37,38^ Subsequently, a conformational search using metadynamics was performed in order to select the three lowest-energy conformers using CREST software.^39^ Regarding the enzyme, the adopted protonation state was determined considering the intracellular pH 7.4, and charge addition to the amino acid residues was generated by APBS-PDB2PQR software.^40^

### 2.4 Molecular Docking and Validations

The molecular docking was performed through the GOLD^41^ software for all compounds. The box size was set to 8.0 Å, and its location in the enzyme 2H7C was determined by the description published by Bencharit and coworkers, where the catalytic site is formed by Ser221, His468, and Glu354.^28^ The simulation generated a total of 90 conformers for each compound, considering that the molecular docking was made as a triplicate, and the search efficiency was set to 100%. The conformers of the redocking process were analyzed according to the average root-mean-square deviation (RMSD) related to the scores available: ChemPLP, GoldScore, ChemScore, and ASP.

Additionally, the GoldScore was selected, and the conformers generated by the docking simulation were evaluated according to the average shorter distances between the conformer carbonyl groups and the Ser221 to represent a more favorable geometrical arrangement within the catalytic site.

### 2.5 Molecular Dynamics Simulations

The MD simulations were carried out using the GROMACS (version 2022.4) software.^42,43^ The parameterization of the ligand systems for AMBER03 ^44^ (also the adopted force-field simulation) was made through ACPYPE.^45^ The complete ligand-receptor system was inserted in a triclinic simulation box filled with TIP3P water molecules and neutralized with 0.15 M NaCl. The long-range interactions were implemented using the particle mesh Ewald (PME) electrostatics with a cutoff of 1.0 nm, and general constraints were implemented through the LINCS algorithm. The geometry minimization was performed with the steepest descent algorithm^46^ followed by two 20 ns equilibration steps at 300 K: (**i**) a canonical NVT ensemble with a V-rescale thermostat;^47^ and (**ii**) an isothermic-isobaric NPT ensemble with V-rescale thermostat and Parrinello-Rahman barostat.^48^ For the MD production step, an NPT ensemble was used with the Leap-Frog integrator^49^ for 300 ns at the set temperature. For each ligand, a triplicate was performed using the three best conformers obtained from the molecular docking stage. The RMSD analysis was used to observe the difference between the triplicate simulations and the average atomic positions of the C-alpha to C-alpha backbone through the trajectories.

### 2.6 MM/PB­(GB)­SA Binding Free Energy

The binding free energy (Δ*G_bind_*) of the receptor-ligand complex systems were realized through Molecular Mechanics Poisson-Boltzmann and Generalized-Born Surface Area (MM/PB­(GB)­SA) simulations using the gmx_mmpbsa tool. The Δ*G_bind_* can be described as:^50,51^

1 $$\Delta G_{bind}=\Delta H+T\Delta S\approx \Delta E_{MM}+\Delta G_{sol}+T\Delta S$$

2 $$\Delta E_{MM}=\Delta E_{ele}+\Delta E_{VDW}$$

3 $$\Delta G_{sol}=\Delta G_{polar}+\Delta G_{nonpolar}$$

The molecular mechanics potential energy, electrostatic energy, van der Waals energy and polar and nonpolar Δ*G_sol_* solvent contributions are Δ*E_MM_*, Δ*E_ele_*, Δ*E_VDW_*, Δ*G_polar_* and Δ*G_nonpolar_*, respectively.

## 3 Results and Discussion

### 3.1 Docking Validation (Redocking)

The redocking is a commonly used step to validate the molecular docking setting. It uses the enzyme crystallographic structure with a cocrystallized ligand as a reference for molecular docking setting determination. This co-crystallized ligand is isolated from the complex and used as a molecular docking ligand with different settings to generate different RMSD results that will be compared to select the software settings corresponding to the lowest value.^52–54^

The best performing score was GoldScore with an RMSD of 4.6 Å, while ChemPLP, ChemScore, and ASP resulted in 5.4 Å, 6.3 Å, and 5.2 Å, respectively. It is important to highlight that although the RMSD is a commonly used parameter to assess the docking validation, in which an RMSD equivalent to <2 is considered successful and values corresponding to >3 are usually related to docking failure, there are limitations to this interpretation when using a flexible and peptide-like ligand.^55,56^

This happens due to the fact that the RMSD function compares the correspondence between two conformers and quantifies the difference between them. Other than that, the calculation is not made considering the protein environment. Additionally, the RMSD value is highly dependent on the number of atoms and how symmetric the ligand in question is.^55,57^

Considering the tendency of obtaining higher RMSD values when working with large ligands such as the coenzyme A, the best score selection was also based on the similar position presented by the conformers when compared to the co-crystallized ligand, through the interaction diagrams provided by Discovery Studio 2019 software.^34^ Despite the best RMSD value being superior to 2.0 Å, the result was considered acceptable once the co-crystallized ligand is large and flexible.^28^

### 3.2 Molecular Docking

The generated ligand conformers using CREST were used as the starting structures of the molecular docking, always selecting the three lowest-energy conformers. The results were evaluated using both scoring and mechanistic criteria. Particular emphasis was given to the distance between the prodrug carbonyl group and Ser221, as well as to the orientation of the ligand within the catalytic site, since these parameters were associated with geometries compatible with the proposed esterase-mediated hydrolysis mechanism.^58–60^ If these criteria are met, the docking structures are considered successful at this stage. The molecular docking calculations were used solely to generate physically reasonable starting poses for the molecular dynamics simulations and were not intended to rank the compounds according to their binding affinity.

#### 3.2.1 Prodrugs: Compounds 1 and 2

Considering compounds 1 and 2, the first one had the best results of the docking triplicate, adopting the aforementioned criteria: dock 2 (distance = 2.0 Å), 9 (distance = 1.9 Å), and 19 (distance = 2.2 Å) showed the three lowest energy results generated by the CREST software as the lowest-energy structures (Table 2). Additionally, regarding the 90 conformers generated for compounds 1, 11 (∼12.2%) had distances between the carbonyl group and the serine hydroxyl group inferior to 3.0 Å. Figure 1 shows dock 2, 9, and 19, in addition to the overlay of the conformers inside the active site. Thus, it is possible to observe that the best structures seemed to fit similarly inside the target, where the dendrimer core was located deeper in the pocket in comparison to the ciprofloxacin portion.

**Figure 1:**
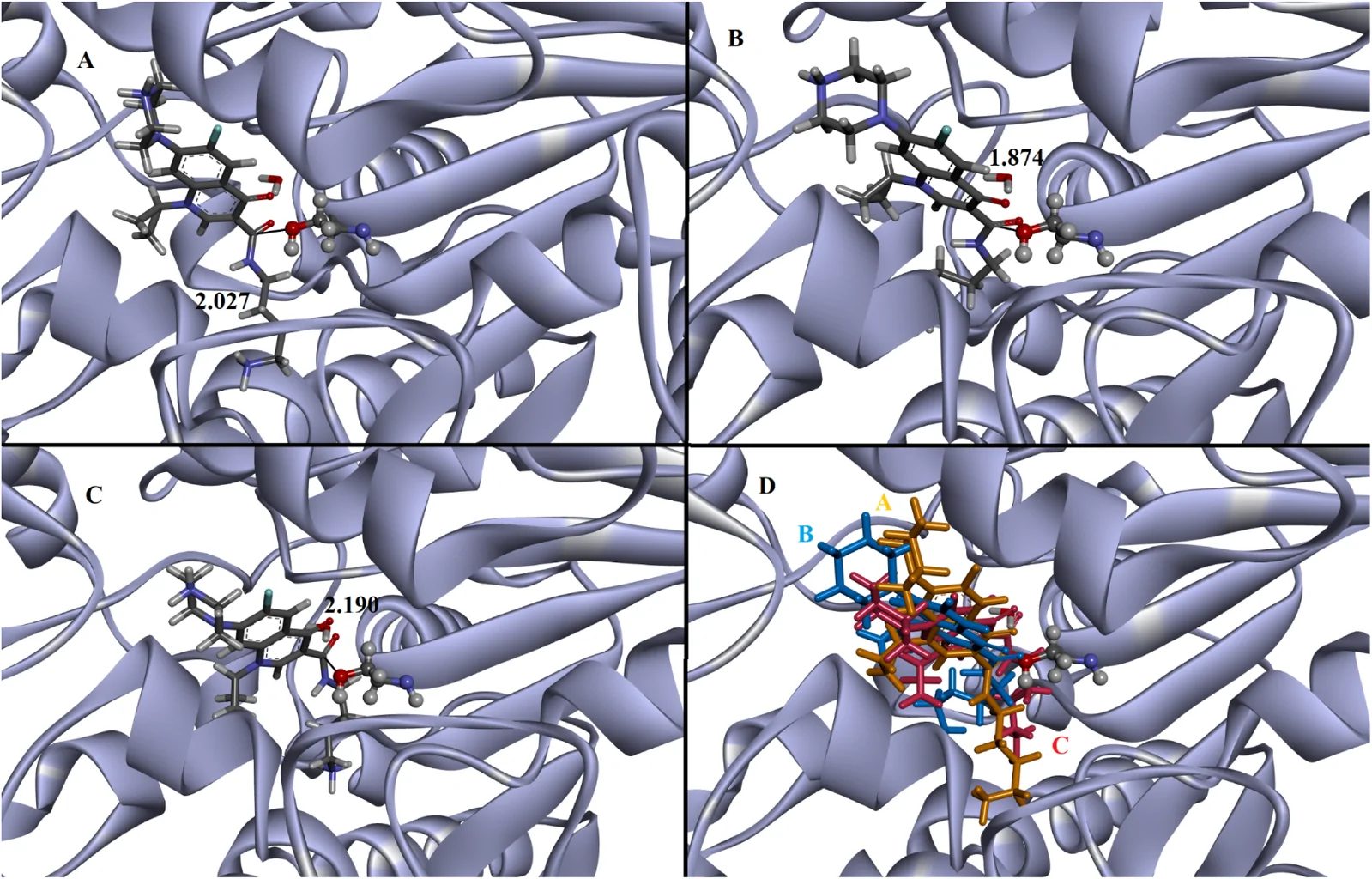
**A**, **B**, and **C** are the best conformers of Compound 1: dock 2, 9, and 19, respectively. And **D** represents the superpositions of those structures in the active site. All distances are given in Å.

**Table 2:** Best Conformers from Compounds 1 and 2 Selected for the Molecular Dynamics Simulations

**Name**	**Simulation**	**Dock**	**Distance (Å)**
Compound 1	1	2	2.0
	2	9	1.9
	3	19	2.2
Compound 2	1	20	2.2
	2	2	3.2
	3	8	1.9

On the other hand, the best results corresponding to the compound 2 were dock 20 (distance = 2.2 Å), 2 (distance = 3.2 Å), and 8 (distance = 1.9 Å) (Table 2). Moreover, compound 2 had 6 out of 90 (∼6.7%) of conformers with distances inferior to 3.0 Å, fewer than compound 1. Figure 2 presents dock 20, 2, and 8, as well as the comparison among their positions inside the active site. The selected structures fit differently inside the enzyme site.

**Figure 2:**
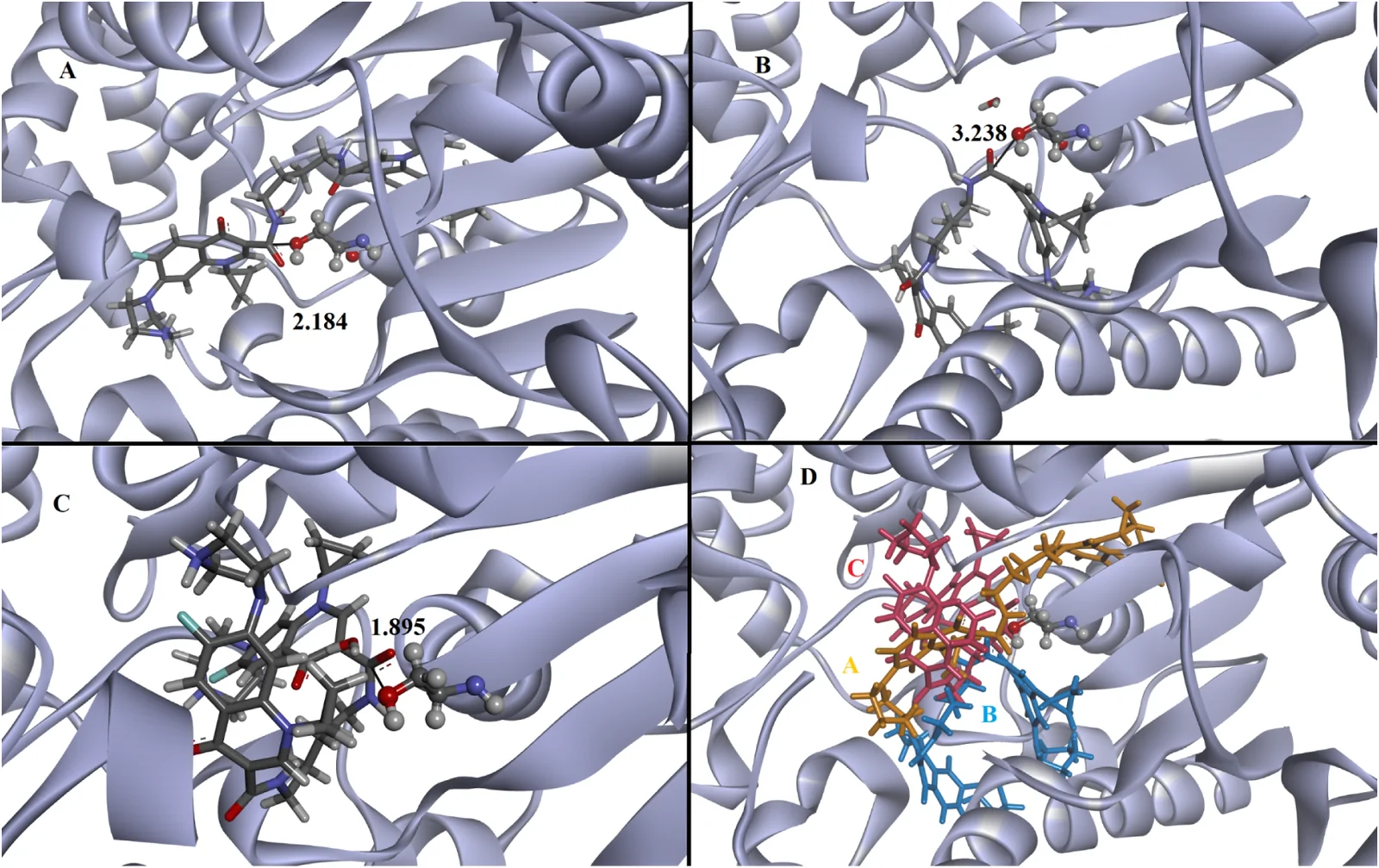
**A**, **B**, and **C** are the best conformers of Compound 2: dock 20, 2, and 8, respectively. And **D** represents the superpositions of those structures in the active site. All distances are given in Å.

#### 3.2.2 Dendrimer Prodrugs: Compounds 3 to 6

Two possible modes of interaction with SER221 were found for dendrimer prodrugs (Compounds 3–6): (**i**) through the carbonyl groups closer to the dendrimer (in blue in Figure 3), referred to as C1; and (**ii**) through the carbonyl groups closer to the drug (in yellow in Figure 3), referred to as C2.

**Figure 3:**
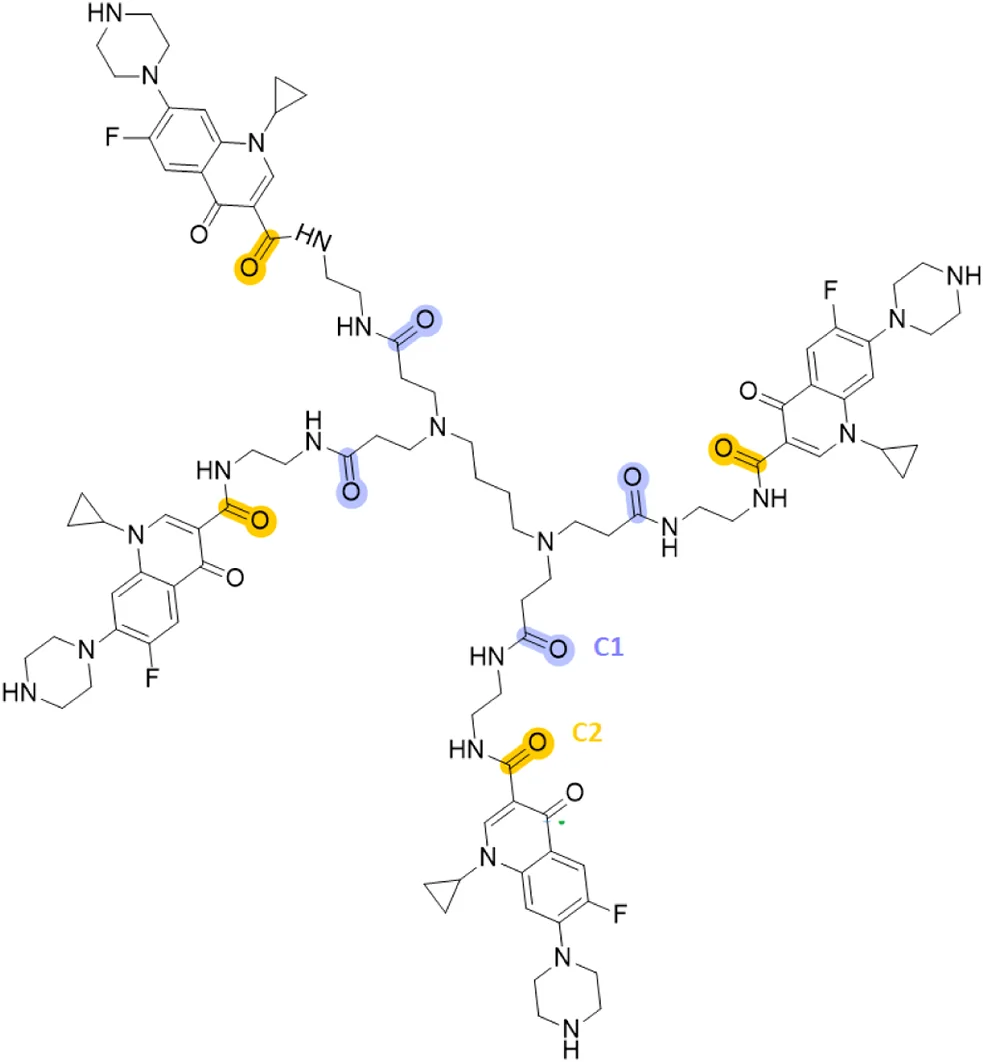
Representation of the carbonyl groups considered in this study. C1: carbonyl groups closer to the dendrimer (in blue), and C2: carbonyl groups closer to the ciprofloxacin (in yellow).

The best conformers of compound 3 were dock 22 (distance = 2.4 Å and C2 ≥ 5.0 Å), 14 (distance = 1.9 Å and C2 ≥ 5.0 Å), and 3 (distance = 1.3 Å and C2 ≥ 5.0 Å). Following the same sequence, compound 4 showed the conformers dock 12 (distance = 1.8 Å and C2 ≥ 5.0 Å), 2 (distance = 1.3 Å and C2 ≥ 5.0 Å), and 13 (distance = 2.5 Å and C2 ≥ 5.0 Å) as the best conformers. Compound 5 presented the most promising results for docks 23 (C1 distance = 3.5 Å and C2 = 3.5 Å), 4 (C1 distance = 2.9 Å and C2 = 4.3 Å), and dock 26 (C1 distance = 2.6 Å and C2 = 4.4 Å). Finally, compound 6 resulted in docks 7 (C1 distance = 2.4 Å and C2 = 4.8 Å), 4 (C1 distance = 4.3 Å and C2 = 1.9 Å), and 5 (C1 distance = 2.3 Å and C2 ≥ 5.0 Å).

Regarding the total number of carbonyl groups of the compounds that exhibited distances shorter than 3.0 Å relative to the hydroxyl group of Ser221, Compound 3 showed for distance C1: 24 out of 90 conformers (∼26.7%) with distances shorter than 3.0 Å, and none for distance C2. In contrast, compound 4 generated a total of 7 out of 90 conformers for C1 (∼7.8%) and 1 out of 90 for C2 (∼1.1%). Compound 5 likewise presented a total of 7 out of 90 conformers for C1 (∼7.8%) and none with distances shorter than 3.0 Å for C2. Finally, compound 6 exhibited 12 out of 90 conformers for C1 (∼13.3%) and 3 out of 90 conformers for C2 (∼3.3%). The positions of the best conformers selected for molecular dynamics simulations can be observed in Figures 4–7, in which it is noticeable that all were located within the active site. However, as the compound size increased, because of their larger molecular size, Compounds 5 and 6 occupied a larger portion of the catalytic pocket, with distal regions extending beyond the immediate vicinity of Ser221 while maintaining the reactive carbonyl group oriented toward the catalytic residue.

**Figure 4:**
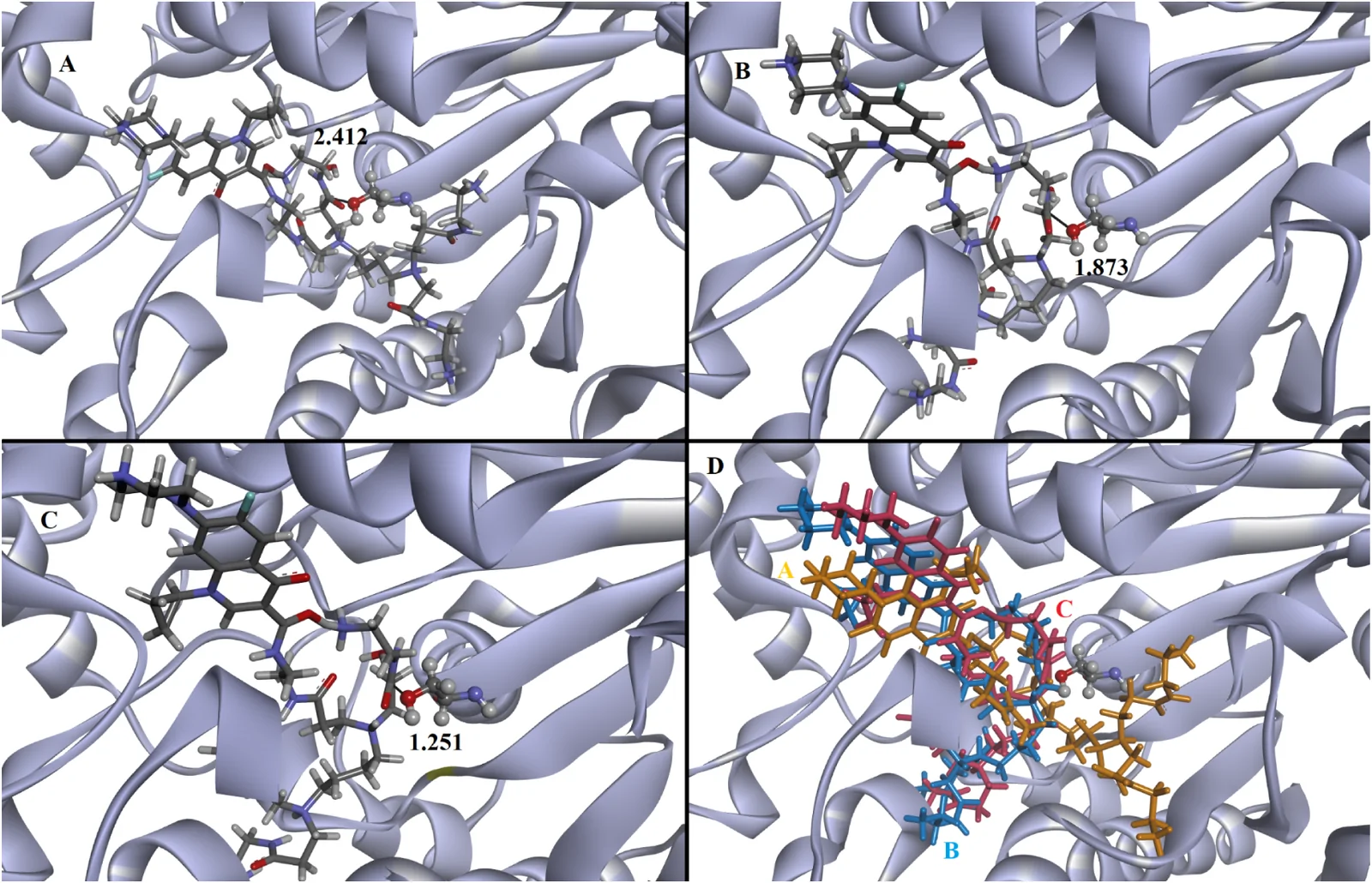
**A**, **B**, and **C** are the best conformers of Compound 3: dock 22, 14, and 3, respectively. And **D** represents the superpositions of those structures in the active site. All distances are given in Å.

**5:**
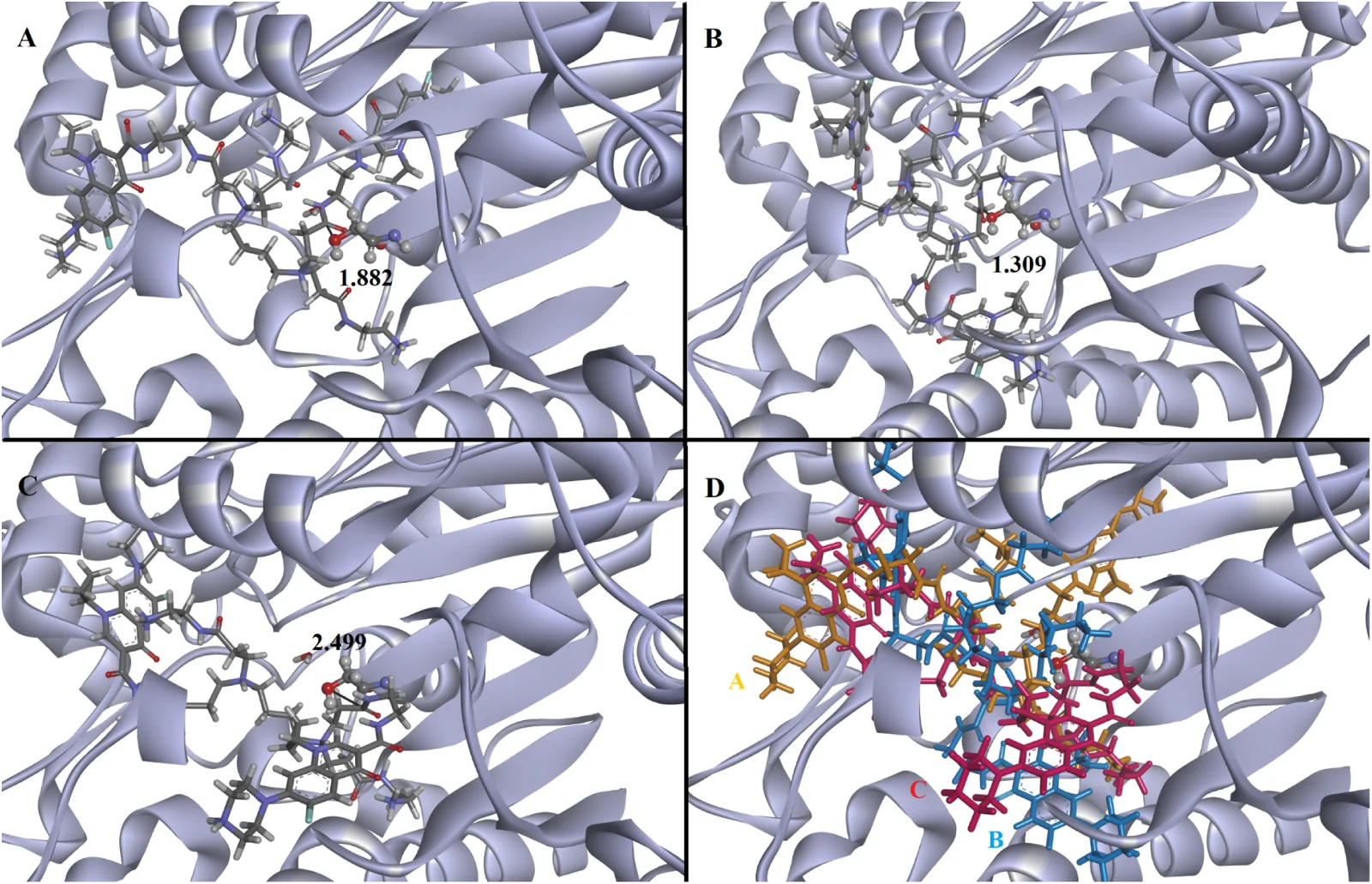
**A**, **B**, and **C** are the best conformers of Compound 4: dock 12, 3, and 13, respectively. And **D** represents the superpositions of those structures in the active site. All distances are given in Å.

**6:**
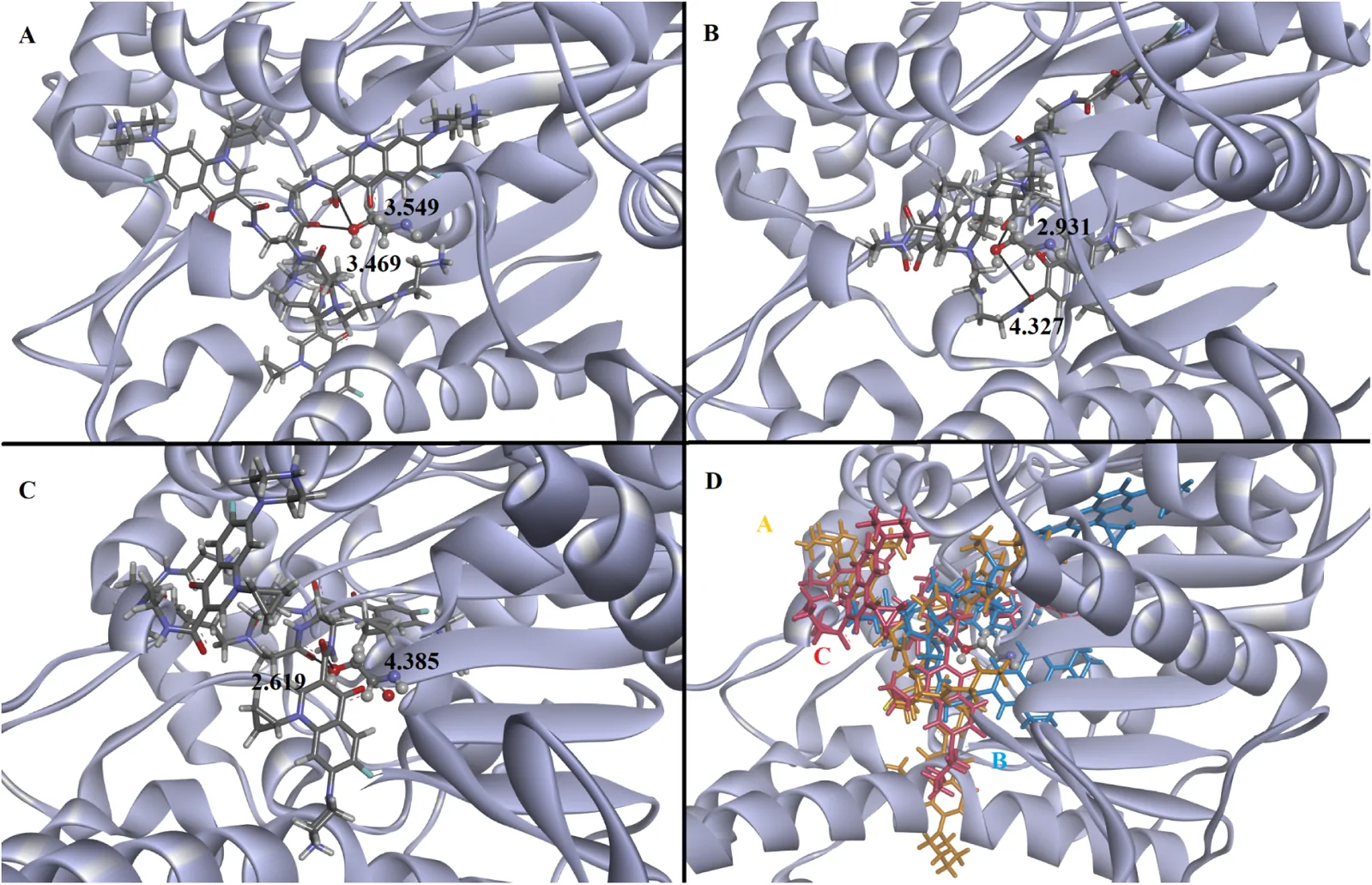
**A**, **B**, and **C** are the best conformers of Compound 5: dock 23, 4, and 26, respectively. And **D** represents the superpositions of those structures in the active site. All distances are given in Å.

**Figure 7:**
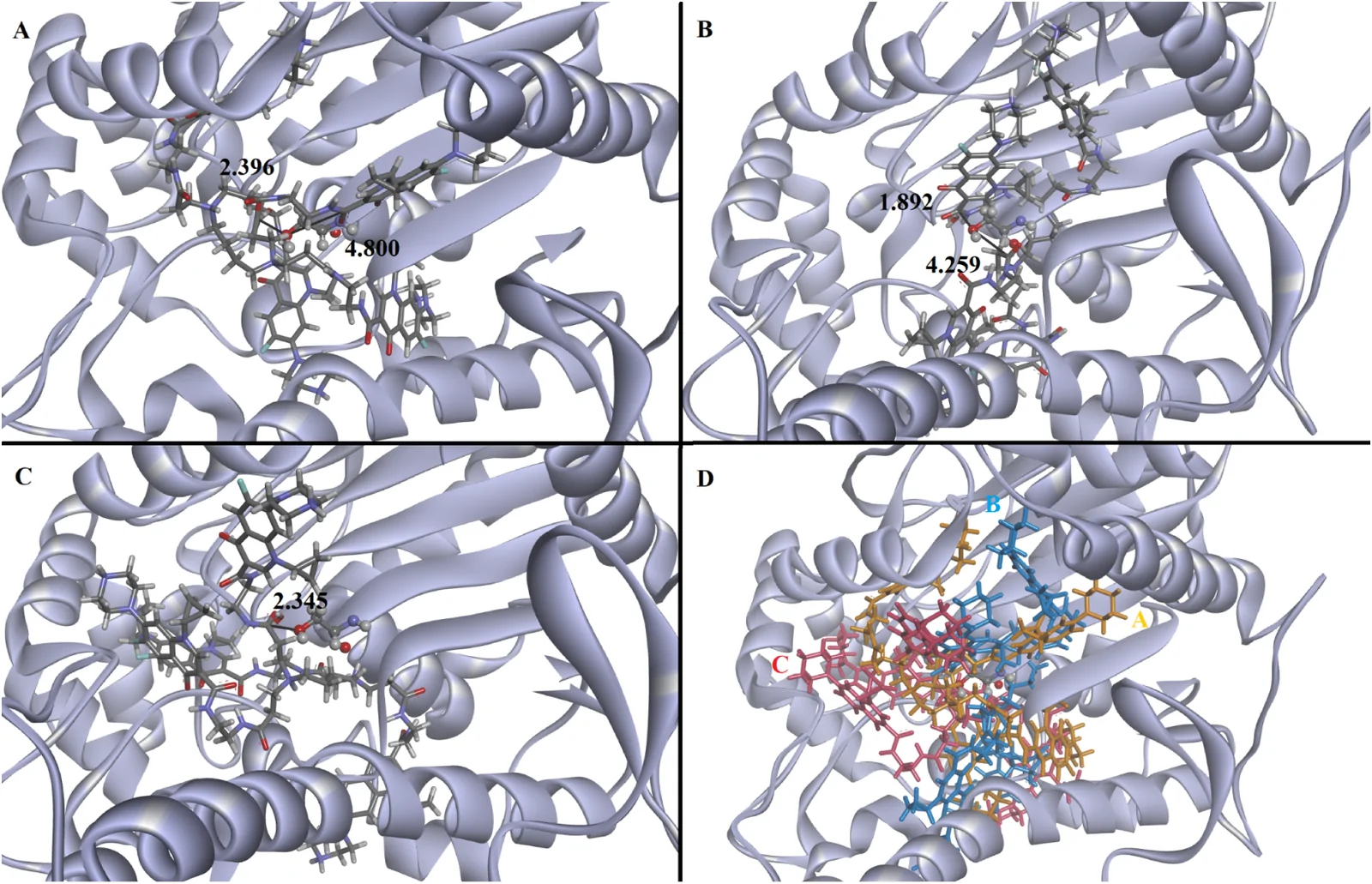
**A**, **B**, and **C** are the best conformers of Compound 6: dock 7, 4, and 5, respectively. And **D** represents the superpositions of those structures in the active site. All distances are given in Å.

In view of the obtained data, interactions involving Ser221 were more frequently observed through the carbonyl group closer to the dendrimer carbonyl group (C1) than closer to the ciprofloxacin (C2). Therefore, those atoms were chosen for the analysis described in this paper. Table 3 is a summary of the selected conformers of this series for the molecular dynamics simulations. Additionally, the docking results should be interpreted as evidence of active-site accommodation rather than proof of enzymatic hydrolysis.

**Table 3:** Best Conformers from Compounds 3 to 6 Selected for the Molecular Dynamics Simulations^a^ ^b^ ^c^

			**Distance (Å)**	
**Name**	**Simulation**	**Dock**	C1^a^	C2^b^
Compound 3	1	22	2.4	—^c^
	2	14	1.9	—
	3	3	1.3	—
Compound 4	1	12	1.8	—
	2	3	1.3	—
	3	13	2.5	—
Compound 5	1	23	3.5	3.5
	2	4	2.9	4.3
	3	26	2.6	4.4
Compound 6	1	7	2.4	4.8
	2	4	4.3	1.9
	3	5	2.3	—

a Carbonyl group closer to the dendrimer core.

b Carbonyl group closer to the ciprofloxacin.

c — Distance from the carbonyl group closer to the Ser221 was superior to 5.0 Å.

### 3.3 Molecular Dynamics Simulations (MD)

The MD simulations were performed in order to investigate the dynamics of the receptor-ligand interaction in an explicit solvent medium across time-temperature evolution, resulting in trajectory files.

The Potential Energy of Interaction (PEI) was calculated to analyze the receptor-ligand complexes’ interaction in an aqueous medium. The PEI analysis consists of short-range interaction energies: Lennard-Jones and Coulomb. Particularly, the Lennard-Jones potential aims to describe the van der Waals interactions occurring among atoms that are close enough to each other. This allows the interaction between the electron density regions in a way that induces a charge fluctuation, generating unspecific attraction by creating a balance between repulsion and attraction forces. On the other hand, Coulomb electrostatic interactions happen among two electrically charged atoms.^61–63^

The PEI calculations were performed using selected RMSD results. Thus, it was the trajectories files that showed greater structural stability (Figures S1 to S6 – Supplementary Material). It is important to note that the RMSD is related to the conformational stability of the receptor-ligand during the molecular dynamics simulation. Thereby, it compares the positions of the atoms (C-alpha to C-alpha reference points in the protein), building the MD parameterization from an initial reference. As a result, low RMSD values indicate that the system maintains conformations close to the reference structure of the simulation, reflecting structural stability, whereas higher values indicate significant conformational deviations from the reference structure.^56,64^

In addition to global ligand-protein PEI results, interactions with the active-site residue Ser221 were analyzed to verify whether similar trends were maintained at the catalytic site (Tables S1 and S2, Supplementary Material). PEI values were subsequently compared across ligands to assess interaction strength. It was also considered the results from Root Mean Square Fluctuation (RMSF), Radius of Gyration (Rg), and number of hydrogen bonds.^65–67^

#### 3.3.1 Prodrug Design: Compounds 1 and 2

Compounds 1 and 2 showed the RMSD values between 1.5 and 1.9 Å, within the expected RMSD range of ≤2.0 Å. Thus, the trajectories files regarding receptor-ligand complexes can be considered reliable for evaluation in triplicate. In addition, the PEI values suggested a strong interaction of the ligands inside the active site. The respective results for each simulation of compound 1 were −258.2 kJ/mol, −217.3 kJ/mol, and −369.4 kJ/mol. Compound 2 presented −538.2 kJ/mol; −493.8 kJ/mol; and −577.0 kJ/mol (Figure S1 – Supplementary Material). Table 4 summarizes the results obtained for compounds 1 and 2.

**Table 4:** Molecular Dynamics Results of Compounds 1 and 2 Considering Triplicate Simulations

	**Average Distance (Å)**	**The Best Distance (Å)**	**Average PEI (**kJ/mol)	**Average RMSD (Å)**
Compound 1	6.5	3.8	–281.6	1.6
Compound 2	7.3	4.5	–536.4	1.8

The RMSF (average values: 1.2 Å for compound 1 and 1.2 Å for compound 2) and radius of gyration (Rg) (average values: 2.3 nm for compound 1 and 2.3 nm for compound 2) showed highly similar profiles across triplicates, indicating consistent behavior between simulations and comparable structural stability for both models. The systems exhibited average RMSD values ≤2.0 Å; however, compound 2 presented lower energy values, suggesting stronger receptor–ligand interactions within the active site (Figure S2, Supplementary Material).

Regarding triplicate trajectories of each ligand-receptor complex, the best result from PEI energy was selected to calculate the ligand-Ser221 energy interaction. Compound 1 had the average Ser221-ligand energy of −4.1 kJ/mol, while compound 2 had −15.7 kJ/mol, and varied from −3.1 kJ/mol: compound 1 to −21.2 kJ/mol: compound 2, also indicating important interactions between Ser221 and compound 2 (Table S1 – Supplementary material).

Ultimately, both compounds remained inside the active site during the MD simulation. The number of interactions between the ligands and the protein possibly contributed to these findings. The average of the hydrogen bond was around 2.8 across the triplicates for compound 1, and 2.9 for compound 2. Figure 8 shows the interaction diagrams for the best conformation of compounds 1 and 2, in addition to a 2D illustration of the ligands inside the enzyme active site.

**Figure 8:**
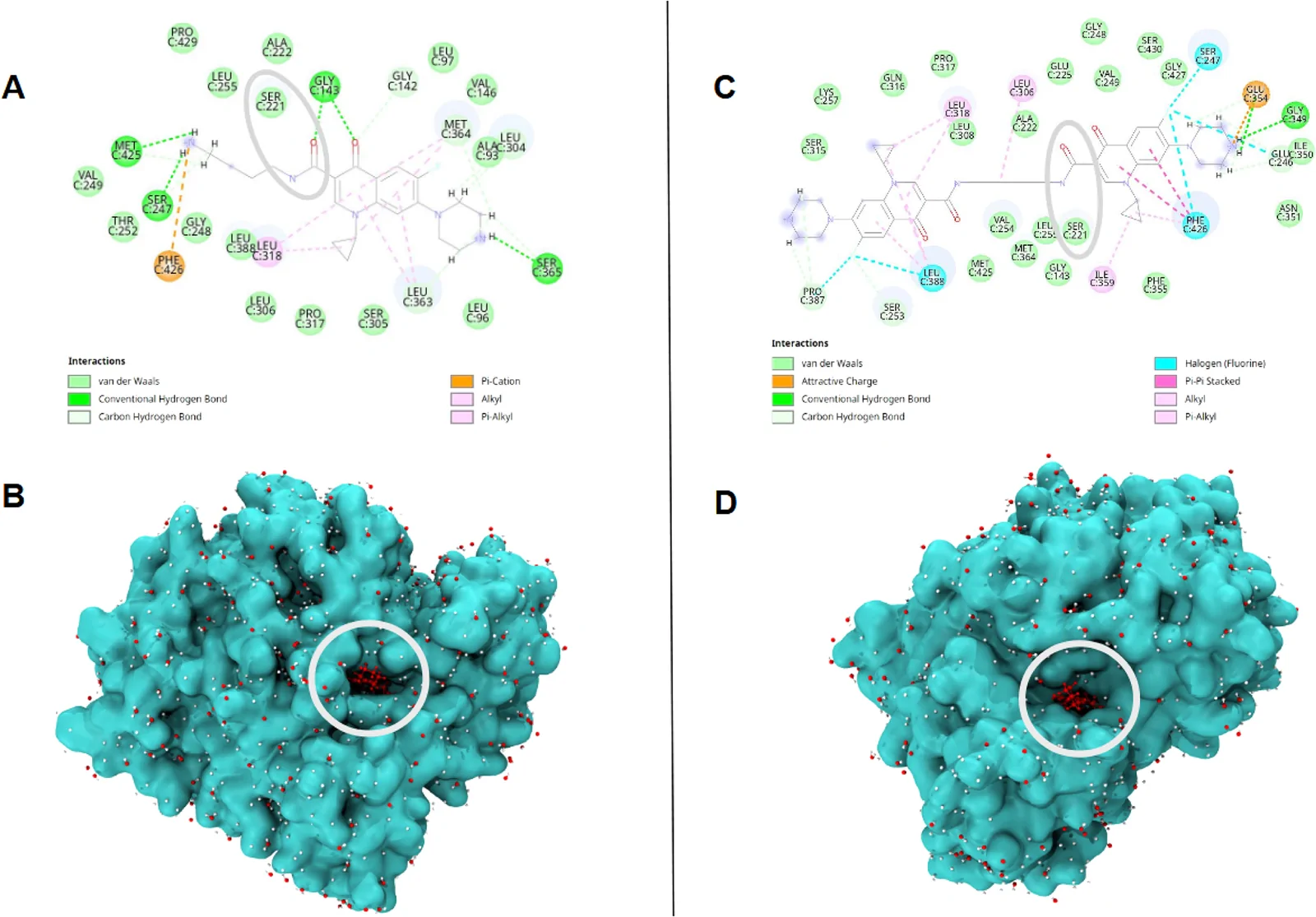
Best molecular dynamics results for compounds 1 and 2. **A**) Compound 1 2D diagram of interactions. **B**) Compound 1 (red) within 2H7C (blue) active site. **C**) Compound 2 2D diagram of interactions. **D**) Compound 2 (red) within 2H7C (blue) active site.

#### 3.3.2 Dendrimer Prodrug Design: Compounds 3 to 6

The RMSD values for compounds 3 to 6 varied from 1.8 to 2.6 across the triplicates. As previously mentioned, despite the ideal RMSD value being equal to or less than 2.0 Å, the values were considered satisfactory considering the size and flexibility of the designed ligands. Recently published *in silico* results in the drug design field presented higher RMSD values for smaller and less flexible molecules in comparison to the ones in the dendrimeric prodrugs compound series.^68–70^ Therefore, the receptor-ligand interactions in the explicit solvent medium of these systems are properly described by the MD trajectories.

The PEI values generated also suggested strong stability of the ligands for this series of compounds. The data related to the triplicate of compound 3 were: −929.8 kJ/mol; −787.5 kJ/mol; and −623.5 kJ/mol. The compound 4 results included the values: −947.8 kJ/mol; −878.4 kJ/mol; and −721.4 kJ/mol. Regarding compound 5, the energy values were: −1013.25 kJ/mol; −1068.16 kJ/mol; and −998.96 kJ/mol. Finally, for the compound 6: −893.27 kJ/mol; −1322.7 kJ/mol; and −990.779 kJ/mol. Those results suggested that the larger structures (compounds 5 and 6) exhibited more favorable PEI values in comparison to the smaller structures (compounds 3 and 4). Table 5 summarizes the results obtained for compounds 3 to 6.

**Table 5:** Molecular Dynamics Results of the Triplicates Obtained for Compounds 3 to 6

	**Average Distance (Å)**	**The Best Distance (Å)**	**Average PEI** (kJ/mol)	**Average RMSD (Å)**
Compound 3	5.2	2.9	–780.3	2.0
Compound 4	6.3	2.9	–849.2	2.5
Compound 5	5.0	2.8	–1026.8	2.0
Compound 6	6.0	3.0	–1061.9	2.2

The potential dendrimer prodrugs series showed RMSF results behaving similarly among triplicates and the compounds (general averages for compound 3: 1.3 Å, compound 4: 1.4 Å, compound 5: 1.3 Å, and compound 6: 1.4 Å). Similarly, the Rg results (general averages for compound 3: 2.3 nm, compound 4: 2.2 nm, compound 5: 2.0 nm, and compound 6: 2.2 nm) demonstrated the compounds behaved almost identically (Figures S3 to S6 – Supplementary material). Although compounds 3 and 5 presented better RMSD results, while the other ones had RMSD results superior to 2.0 Å, the PEI average values indicated more favorable results for stability between the complexes for compounds 5 and 6 (Table 5).

Likewise, to the previous series, one conformer was selected from the molecular dynamics in order to represent the conformation exhibiting the most favorable Ser221 interaction profile among the simulated trajectories. The average energy values for Ser221 and the designed ligands were also calculated for those promising conformations. The Ser221-ligand energies for this series were for compound 3: −6.5 kJ/mol, compound 4: −6.9 kJ/mol, compound 5: −15.5 kJ/mol, and compound 6: −23.0 kJ/mol, and varied from −0.1 kJ/mol: compound 4 to −56.8 kJ/mol: compound 6 (Table S2 – Supplementary material), suggesting a better potential interaction of this amino acid with compounds 5 and 6.

It is important to note that the entire set of compounds remained within the active site over the entire simulation, likely due to the quantity of hydrogen bond interactions formed between the ligands and the esterase. The general average of hydrogen bond formation for each simulation was for compound 3: 7.7, compound 4: 8.5, compound 5: 9.6, and compound 6: 7.9, indicating that although the compounds have different sizes, they seem to have similar amounts of hydrogen bond numbers. Figure 9 illustrates the interaction diagrams for the best conformation of compounds 3 to 6, in addition to an illustration of the ligands inside the enzyme.

**Figure 9:**
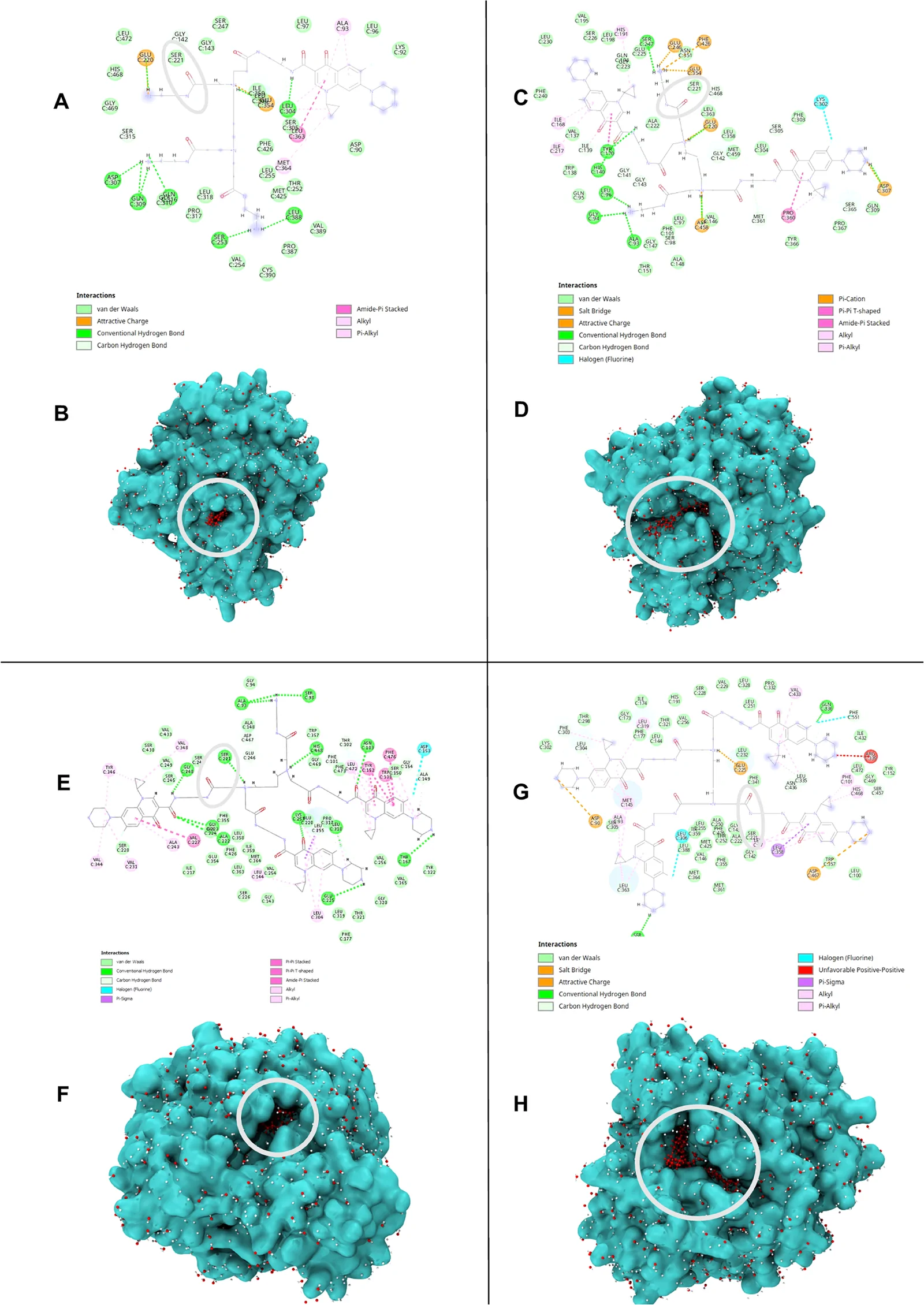
Best molecular dynamics results for Compounds 3 to 6. **A**) Compound 3 2D diagram of interactions. **B**) Compound 3 (red) within 2H7C (blue) active site. **C**) Compound 4 2D diagram of interactions. **D**) Compound 4 (red) within 2H7C (blue) active site. **E**) Compound 5 2D diagram of interactions. **F**) Compound 5 (red) within 2H7C (blue) active site. **G**) Compound 6 2D diagram of interactions. **H**) Compound 6 (red) within 2H7C (blue) active site.

In general, the atom position and energy RMSD results, similar to the Tot-Drift values, although not ideal, were considered favorable due to the size and flexibility of the designed ligands (Figures S1–S6 – Supplementary Material), suggesting a better potential interaction of Ser221 with the compounds 5 and 6. The large and flexible ligands usually present relevant energy differences between conformations and are also more susceptible to calculation errors because of the high amount of intra and intermolecular interactions, resulting in RMSDs and energy drift values with different patterns and references in comparison to the data obtained from small ligands.^71–73^ However, the obtained PEI results indicate a trend toward stronger receptor–ligand interactions as the number of dendrimer branches increases. The specific ligand-Ser221 results showed that the observed trend is also followed by the direct interaction with the protein active-site, corroborating that not only the chain additions increase interaction, but also the enhanced dendrimer-Ser221 interaction.

#### 3.3.3 MM/PBSA and MM/GBSA Binding Free Energy Analysis

To further characterize the interactions between human carboxylesterase 1 and the proposed ciprofloxacin prodrug prototypes, MM/PBSA and MM/GBSA binding free energy calculations were performed using the molecular dynamics trajectories. The trajectory range was selected using the same criterion applied to the PEI analysis, namely the most stable region of the RMSD profiles. Since both approaches produced similar energetic trends, only the average MM/PBSA binding free energies are discussed herein, whereas the complete MM/PBSA and MM/GBSA data sets are provided in the Supplementary Material (Tables S3 and S4 – Supplementary Material).

An overall increasing trend in protein–ligand binding affinity was observed from Compound 1 to Compound 6, with ΔG_bind_ values ranging from approximately −648.44 to −125.77 kJ/mol (Table S3 – Supplementary Material). The most favorable interactions were obtained for Compounds 5 and 6, whereas Compound 1 presented the least favorable potential interaction of this study. This trend is consistent with the larger molecular size and greater number of ciprofloxacin moieties present in the higher-generation derivatives, which increase the available surface for intermolecular contacts within the catalytic pocket (Tables 6 and 7).

**Table 6:** Average MM/PBSA Binding Free Energies Calculated for the Interaction of the Proposed Ciprofloxacin Prodrug Prototypes with Human Carboxylesterase 1

**Energy** (kJ/mol)	**Compound 1**	**Compound 2**	**Compound 3**	**Compound 4**	**Compound 5**	**Compound 6**
van der Waals energy	–200.79 ± 12.84	–394.50 ± 17.80	–367.31 ± 26.94	–499.60 ± 22.96	–741.54 ± 27.61	–823.02 ± 36.01
Electrostatic energy	–1255.37 ± 89.72	–1434.61 ± 92.51	–5198.24 ± 232.97	–4292.87 ± 226.26	–4203.32 ± 251.37	–3851.73 ± 291.21
Polar solvation energy	1328.17 ± 80.77	1595.97 ± 89.33	5254.69 ± 224.22	4510.56 ± 216.73	4555.71 ± 236.05	4198.83 ± 272.35
SASA energy	–21.35 ± 0.57	–33.47 ± 0.64	–41.34 ± 1.55	–51.88 ± 1.63	–65.21 ± 1.41	–76.72 ± 1.87
Binding free energy	–149.35 ± 24.11	–266.60 ± 31.95	–352.21 ± 61.48	–333.77 ± 44.38	–454.38 ± 50.86	–552.65 ± 51.92

**Table 7:** Average MM/PBSA Binding Free Energies Calculated for the Interaction of the Proposed Ciprofloxacin Prodrug Prototypes with Ser221 of the Human Carboxylesterase 1

**Energy** (kJ/mol)	**Compound 1**	**Compound 2**	**Compound 3**	**Compound 4**	**Compound 5**	**Compound 6**
van der Waals energy	–3.82 ± 1.90	–20.29 ± 3.29	–8.21 ± 3.88	–7.70 ± 2.08	–14.00 ± 3.01	–13.78 ± 2.91
Electrostatic energy	9.32 ± 8.74	24.49 ± 7.67	28.44 ± 14.63	23.04 ± 8.47	15.66 ± 7.70	–20.84 ± 12.80
Polar solvation energy	–2.78 ± 8.26	–4.83 ± 6.15	–14.71 ± 14.57	–14.70 ± 8.19	–2.89 ± 7.25	33.47 ± 12.08
SASA energy	–1.42 ± 0.39	–3.31 ± 0.21	–2.26 ± 0.53	–1.97 ± 0.47	–3.12 ± 0.26	–3.11 ± 0.40
Binding free energy	1.28 ± 2.85	–3.93 ± 3.75	3.28 ± 5.49	–1.32 ± 3.81	–4.37 ± 3.85	–4.24 ± 3.24

The MM/PBSA results complement the PEI analysis previously discussed. Although both approaches evaluate ligand–protein interactions, they are not directly equivalent. The PEI calculations are mainly based on Coulombic and van der Waals contributions, whereas MM/PBSA additionally incorporates solvent effects through polar and nonpolar solvation terms. Consequently, differences in the absolute energetic values are expected.^74–77^ Nevertheless, both approaches consistently indicated more favorable interactions for the larger dendrimer-based prodrug compounds.

The specific interaction between Ser221 and the ligands presented smaller energetic contributions than those observed for the entire protein–ligand complexes. In several cases, ΔG_bind_ values were close to zero. This behavior should not be interpreted as the absence of interaction between Ser221 and the ligands. Because MM/PBSA energies are obtained from the sum of multiple energetic components, small final values may arise from the partial cancellation of these contributions. Therefore, values close to zero indicate a limited net energetic contribution of Ser221 to the overall binding free energy rather than a lack of molecular contact, this is also due to the fact that only this one amino acid was analyzed and not the entire protein structure.^76,78^ On the other hand, the highest energy values regarding the ligands and protein interactions were associated with polar solvation energy for all ligands. These findings suggested that the stabilization of the complexes results from the cooperative contribution of multiple residues within the enzyme active site.

Summarizing, the computational analyses performed in this study demonstrated that all investigated ciprofloxacin-based prodrug candidates were capable of being accommodated within the active site of human carboxylesterase 1. Molecular docking identified conformations in which the amide carbonyl remained oriented toward the catalytic region of the enzyme, while molecular dynamics simulations confirmed the stability of the receptor–ligand complexes throughout the simulation period.

Although the non-dendrimeric compounds exhibited shorter distances to Ser221, the introduction of dendrimer branches resulted in a substantial increase in the overall receptor–ligand interaction energies. This behavior suggested that the dendrimeric structures contributed to the stabilization of the complexes through additional intermolecular contacts within the active site. The hydrogen-bond analyses further supported this observation, as all dendrimer-containing compounds maintained multiple interactions during the simulations while remaining accommodated within the catalytic pocket.

The PEI calculations revealed a trend toward stronger receptor–ligand interactions as the number of dendrimer branches increased. Moreover, the specific interaction energies calculated for Ser221 indicated that the enhanced interactions were not restricted to peripheral contacts with the protein but also involved the catalytic region of the enzyme. Overall, these findings suggested that dendrimer functionalization may favor active-site accommodation and promote conformational arrangements compatible with productive enzyme recognition.

The MM/PBSA results corroborated the PEI analysis, indicating increasingly favorable binding free energies for the dendrimer-based prototypes, with van der Waals and electrostatic interactions as the main stabilizing contributions. MM/GBSA showed consistent trends, and near-zero Ser221 interaction energies reflect energetic compensation rather than absence of contact. Therefore, both analyses supported enhanced stabilization of dendrimer-functionalized compounds within the human carboxylesterase 1 active site.

Overall, compounds presenting dendrimeric structures displayed the most favorable interaction profiles, combining active-site accommodation, conformational stability, and stronger interaction energies. These results support the selection of the proposed ciprofloxacin–dendrimer prodrug prototypes for future synthesis and experimental evaluation. Although these results support productive molecular recognition by human carboxylesterase 1, experimental studies are still required to confirm catalytic hydrolysis and prodrug activation.

The present study was designed as an early-stage computational investigation aimed at evaluating the structural compatibility between the proposed ciprofloxacin-based prodrug prototypes and the catalytic site of human carboxylesterase 1. Although molecular docking and molecular dynamics simulations provide valuable information regarding ligand accommodation, conformational stability, and the adoption of catalytically relevant orientations, these approaches do not directly demonstrate enzymatic hydrolysis, drug release, or the formation of reaction intermediates. Therefore, the observed interactions with the catalytic machinery of the enzyme should be interpreted as indicators of potential recognition rather than evidence of cleavage of the amide bond.

In addition, the present work does not evaluate reaction kinetics, hydrolysis rates, or the extent of ciprofloxacin release under physiological conditions. Particularly, although the computational analyses identified preferential interactions involving the carbonyl group closer to the dendrimer (C1), the present study does not establish whether enzymatic hydrolysis would proceed through one or multiple cleavage steps, nor whether any intermediate generated prior to complete ciprofloxacin release would retain antibacterial activity. These questions require dedicated experimental investigation.

Likewise, the preservation of the antibacterial activity of the released drug was not investigated and will require experimental confirmation. Furthermore, the proposed improvements in biopharmaceutical properties remain hypothetical at this stage, since no experimental studies regarding solubility, permeability, bioavailability, or pharmacokinetic behavior have yet been performed. Despite these limitations, the computational strategy employed herein provides a rational framework for prioritizing candidates for synthesis and experimental evaluation, contributing to the optimization of resources during the early stages of drug development.

In the development of dendrimer-based drug conjugates, *in silico* approaches such as molecular docking and molecular dynamics simulations are commonly used to prioritize the most promising candidates before experimental synthesis, reducing the number of compounds to be prepared and optimizing time and resources. Once computational compatibility with the biological target has been established, subsequent stages typically include the previously mentioned biopharmaceutical studies.^79–81^ Therefore, the present findings provide a rational basis for advancing the proposed ciprofloxacin–dendrimer conjugates toward synthesis and properties measurement assays.

## 4 Conclusions

This study aimed to investigate the interaction between ciprofloxacin prodrugs with dendrimeric chain modification and human carboxylesterase 1 through a theoretical approach. The receptor-ligand complexes without dendrimeric structures, compounds 1 and 2, were mostly closer to the Ser221 active-site (1.9 to 3.2 Å) in comparison to the other ciprofloxacin prodrugs, compounds 3–6 (1.3 to 4.8 Å). However, this did not correspond directly to enhanced PEI energy values. The compounds 3 to 6 exhibited more favorable receptor–ligand interaction energies with the esterase protein part due to their dendrimer ramifications, which, coupled with ciprofloxacin, increased the total number of intermolecular interactions with the active site.

This can be illustrated by the high PEI values (−281.6 to −1061.9 kJ/mol). The Ser221-ligand interactions also presented higher PEI values (−3.1 kJ/mol: compound 1 to −56.8 kJ/mol: compound 6), showing that not only do larger side-dendrimer chains influence the receptor-ligand interaction, but also the specific dynamics between the ligands and the active-site through the simulations, suggesting that the dendrimer ramifications can be accommodated within the protein pocket. The MM/PBSA data corroborated with the PEI values. The obtained results demonstrated favorable interactions between the designed ciprofloxacin–dendrimer prodrugs prototypes and human carboxylesterase 1, providing structural insights that support their selection for future synthesis, physicochemical characterization, and biological evaluation.

## Supplementary Material



## Notes

Detailed molecular dynamics simulation results for Compounds 1–6, including average simulation data and complete molecular dynamics data sets; Complete MM/PBSA and MM/GBSA results for protein–ligand and Ser221–ligand interactions (PDF)
