# Supplementary material for: Molecular Docking and Molecular Dynamics Studies: Evaluation of the Potential Interaction between Ciprofloxacin-DAB-PAMAM Dendrimer-Based Prodrug Prototypes and Human Carboxylesterase 1
Source: ACS Omega. 2026 Oct 6;11(41):62991–3005. doi: 10.1021/acsomega.6c04630 (PMC13689223; doi:10.1021/acsomega.6c04630)
Supplement: Supplementary file 1 [file ao6c04630_si_001.pdf]

Supplementary Material

Molecular docking and dynamics studies: evaluation of the potential interaction between Ciprofloxacin-DAB-PAMAM dendrimer-based prodrug prototypes and human carboxylesterase 1

Sophia Sueyoshi[1+], Lucas B. Raimo[2+], Lucas P. Coutinho[2+], Marcos O. O. Brito[2+], Jeanine Giarolla[1*], Lucas C. Ducati[2**]

[+]Those authors equally contributed to this work.

[1] Department of Pharmacy, School of Pharmaceutical Sciences, University of São Paulo, Av. Prof. Lineu Prestes, 580, 05508-900 - São Paulo, SP, Brazil
[2] Department of Fundamental Chemistry, Institute of Chemistry, University of São Paulo, Av. Prof. Lineu Prestes, 748, 05508-000 - São Paulo, SP, Brazil
corresponding authors: *jeanineg@usp.br, **ducati@iq.usp.br

The Tables 1 and 2 represent a summary of the average results generated by the molecular dynamics simulations, where the the column data consider the entirety of the simulation and "Best Distance" and "Number of Conformations" specifically are related to the Serine and the ligand. The Table 3 shows the complete MM/PBSA and MM/GBSA datasets calculated from the molecular dynamics simulations.

The Figures 1 to 6 show more details on the remaining calculated information from the simulation. The concepts of RMSD, RMSF, Rg, Distance and Hydrogen bonds number were approached on the main part of this manuscript. The energies calculated mentioned in those figures was PEI which is correlated with an Average value that corresponds to the average of the individual interactions: Short Coulomb and van der Waals.

Table S1. Overview of the molecular dynamics average results obtained for Compounds 1 and 2.

		Average Distance (Å)	Best Distance (Å)	Number of Conformations/Total (0 < conf < 5Å)	Ser-LIG Energy (kJ/mol)	Average PEI (kJ/mol)	Average RMSD (Å)
**Compound 1**	**1**	7.43	5.53	0	-4.754	-258.193	1.55
	**2**	6.27	3.76	2834	-3.052	-217.346	1.82
	**3**	5.65	3.88	5512	-4.432	-369.369	1.56
**Compound 2**	**1**	7.13	5.08	0	-21.203	-538.182	1.81
	**2**	5.68	4.47	561	-15.506	-493.859	1.94
	**3**	9.16	6.61	0	-10.328	-577.029	1.68

Table S2. Overview of the molecular dynamics average results obtained for Compounds 3 to 6.

		Average Distance (Å)	Best Distance (Å)	Number of Conformations/Total (0 < conf < 5Å)	Ser-LIG Energy (kJ/mol)	Average PEI (kJ/mol)	Average RMSD (Å)
**Compound 3**	**1**	6.52	3.17	5675	-4.559	-929.828	1.87
	**2**	4.36	2.89	25866	-3.848	-787.525	1.89
	**3**	4.68	2.96	20773	-11.076	-623.466	2.18
**Compound 4**	**1**	6.39	2.86	10802	-0.055	-947.830	2.55
	**2**	6.15	3.02	2378	-8.072	-878.437	2.20
	**3**	6.27	2.87	11795	-12.554	-721.416	2.64
**Compound 5**	**1**	4.62	3.55	25812	-9.573	-1013.250	1.93
	**2**	4.74	2.76	18120	-28.665	-1068.160	2.06
	**3**	5.62	3.92	1689	-8.203	-998.960	1.85
**Compound 6**	**1**	7.53	3.58	1954	-6.020	-893.276	2.27
	**2**	5.30	3.58	7996	-56.771	-1322.700	2.49
	**3**	5.25	2.96	10152	-6.222	-990.779	1.75

Table S3. Complete MM/PBSA and MM/GBSA datasets for protein-ligand interactions.

Compound	Triplicate	Energy type	PBSA (kJ/mol)	SD (kJ/mol)	SD prop. (kJ/mol)	GBSA (kJ/mol)	SD (kJ/mol)	SD prop. (kJ/mol)
1	1	VDW	-180.33	11.46	4.64	-180.33	11.46	4.64
		EE	-1230.47	93.64	5.10	-1230.47	93.64	5.10
		Polar (ePB)	1287.63	94.06	2.05	1320.34	91.13	4.69
		SASA	-20.42	0.71	0.13	-24.73	1.21	0.00
		Binding (Tot)	-143.59	20.59	7.20	-115.19	10.50	8.33
	2	VDW	-203.01	14.39	5.48	-203.01	14.39	5.48
		EE	-1188.72	92.97	4.02	-1188.72	92.97	4.02
		Polar (ePB)	1287.63	79.75	7.53	1295.53	82.63	4.94
		SASA	-21.63	0.59	0.04	-25.77	1.46	0.13
		Binding (Tot)	-125.77	27.74	10.17	-122.01	17.45	8.41
	3	VDW	-219.03	12.68	5.23	-219.03	12.68	5.23
		EE	-1346.91	82.55	10.67	-1346.91	82.55	10.67
		Polar (ePB)	1409.25	68.49	3.89	1431.39	74.56	1.76
		SASA	-22.01	0.42	0.13	-30.54	1.09	0.13
		Binding (Tot)	-178.70	24.02	12.51	-165.06	16.82	12.01
2	1	VDW	-424.68	17.41	8.12	-424.68	17.41	8.12
		EE	-1385.03	58.83	4.06	-1385.03	58.83	4.06
		Polar (ePB)	1601.13	62.47	5.06	1591.93	59.91	0.88
		SASA	-33.43	0.50	0.21	-48.37	1.13	0.17
		Binding (Tot)	-242.00	43.81	10.42	-266.14	29.83	9.12
	2	VDW	-361.04	18.83	6.78	-361.04	18.83	6.78
		EE	-1366.70	112.26	5.77	-1366.70	112.26	5.77
		Polar (ePB)	1483.86	107.99	8.58	1486.45	107.45	4.56
		SASA	-32.80	0.75	0.21	-42.76	2.13	0.33
		Binding (Tot)	-276.69	26.11	12.38	-284.09	19.75	10.04
	3	VDW	-397.77	17.15	6.65	-397.77	17.15	6.65
		EE	-1552.10	106.44	26.07	-1552.10	106.44	26.07
		Polar (ePB)	1702.93	97.53	28.12	1684.98	96.32	26.07
		SASA	-34.18	0.67	0.04	-50.38	1.46	0.17
		Binding (Tot)	-281.12	25.94	38.91	-315.26	21.88	37.45
3	1	VDW	-387.44	36.19	3.43	-387.44	36.19	3.43
		EE	-5765.26	244.68	62.76	-5765.26	244.68	62.76
		Polar (ePB)	5787.73	230.91	69.20	5957.64	233.72	64.48
		SASA	-40.79	2.13	0.13	-54.94	3.35	0.08
		Binding (Tot)	-405.76	60.63	93.47	-249.95	48.66	90.04
	2	VDW	-354.85	22.09	5.69	-354.85	22.09	5.69
		EE	-4863.73	232.13	50.67	-4863.73	232.13	50.67
		Polar (ePB)	4933.10	227.44	34.06	5088.66	225.48	44.94
		SASA	-41.46	1.17	0.46	-53.72	2.47	0.25
		Binding (Tot)	-326.94	61.88	61.30	-183.64	36.19	67.95
	3	VDW	-359.66	22.55	5.77	-359.66	22.55	5.77
		EE	-4965.74	222.09	26.78	-4965.74	222.09	26.78
		Polar (ePB)	5043.23	214.30	15.06	5196.07	210.66	39.46
		SASA	-41.76	1.34	0.33	-53.93	3.01	0.17
		Binding (Tot)	-323.93	61.92	31.25	-183.26	33.51	48.03

Table S3. Complete MM/PBSA and MM/GBSA datasets for protein-ligand interactions. (cont.)

Compound	Triplicate	Energy type	PBSA (kJ/mol)	SD (kJ/mol)	SD prop. (kJ/mol)	GBSA (kJ/mol)	SD (kJ/mol)	SD prop. (kJ/mol)
4	1	VDW	-464.55	24.89	4.52	-464.55	24.89	4.52
		EE	-4439.01	217.86	44.22	-4439.01	217.86	44.22
		Polar (ePB)	4579.97	199.95	39.16	4682.90	207.19	40.58
		SASA	-48.79	2.01	0.21	-64.06	2.85	0.00
		Binding (Tot)	-372.38	47.11	59.29	-284.76	34.98	60.21
	2	VDW	-516.72	23.30	7.24	-516.72	23.30	7.24
		EE	-4209.94	226.23	4.35	-4209.94	226.23	4.35
		Polar (ePB)	4436.25	215.98	15.48	4515.75	217.61	13.72
		SASA	-55.52	1.26	0.67	-70.58	2.47	0.75
		Binding (Tot)	-345.89	44.48	17.66	-281.46	24.06	16.15
	3	VDW	-517.52	20.67	9.12	-517.52	20.67	9.12
		EE	-4229.65	234.68	17.66	-4229.65	234.68	17.66
		Polar (ePB)	4515.46	234.26	18.83	4551.10	228.53	14.14
		SASA	-51.34	1.63	0.67	-63.68	2.64	0.33
		Binding (Tot)	-283.05	41.55	27.41	-259.78	32.05	24.43
5	1	VDW	-749.52	29.16	1.34	-749.52	29.16	1.34
		EE	-4587.21	294.68	6.19	-4587.21	294.68	6.19
		Polar (ePB)	4904.65	284.47	15.69	4974.99	283.01	6.49
		SASA	-64.64	1.21	0.21	-89.91	2.76	0.42
		Binding (Tot)	-496.77	47.11	16.90	-451.66	35.10	9.08
	2	VDW	-802.70	25.23	13.47	-802.70	25.23	13.47
		EE	-4052.54	208.74	36.90	-4052.54	208.74	36.90
		Polar (ePB)	4517.46	185.64	45.98	4430.52	197.82	31.92
		SASA	-68.12	1.34	0.67	-93.64	2.26	0.71
		Binding (Tot)	-405.89	63.47	60.50	-518.40	31.97	50.67
	3	VDW	-672.41	28.45	10.96	-672.41	28.45	10.96
		EE	-3970.20	250.71	15.94	-3970.20	250.71	15.94
		Polar (ePB)	4245.00	238.03	19.08	4297.39	236.73	25.02
		SASA	-62.89	1.67	0.42	-83.68	2.68	0.17
		Binding (Tot)	-460.49	42.01	27.15	-428.90	31.63	31.63
6	1	VDW	-725.63	43.60	14.81	-725.63	43.60	14.81
		EE	-3628.07	313.13	43.01	-3628.07	313.13	43.01
		Polar (ePB)	3930.53	300.20	3.64	3979.24	308.99	9.37
		SASA	-69.29	2.64	0.46	-90.42	5.31	0.67
		Binding (Tot)	-492.46	52.51	45.65	-464.93	46.11	46.44
	2	VDW	-945.33	31.42	4.60	-945.33	31.42	4.60
		EE	-4212.70	269.24	18.03	-4212.70	269.24	18.03
		Polar (ePB)	4595.04	236.31	48.28	4584.41	244.81	56.27
		SASA	-85.40	1.38	0.46	-113.22	2.47	0.59
		Binding (Tot)	-648.44	56.65	51.71	-686.89	41.88	59.29
	3	VDW	-798.10	33.01	11.46	-798.10	33.01	11.46
		EE	-3714.43	291.25	5.61	-3714.43	291.25	5.61
		Polar (ePB)	4070.91	280.54	30.79	4071.91	273.55	24.69
		SASA	-75.48	1.59	0.50	-98.32	3.51	0.67
		Binding (Tot)	-517.06	46.61	33.35	-538.90	44.85	27.78

Table S4. Complete MM/PBSA and MM/GBSA datasets for serin-ligand interactions.

Compound	Triplicate	Energy type	PBSA (kJ/mol)	SD (kJ/mol)	SD prop. (kJ/mol)	GBSA (kJ/mol)	SD (kJ/mol)	SD prop. (kJ/mol)
		VDW	-3.97	2.09	0.25	-3.97	2.09	0.25
		EE	11.09	14.27	6.82	11.09	14.27	6.82
	1	Polar (ePB)	-0.13	11.51	5.90	-0.38	13.22	7.82
		SASA	-1.72	0.21	0.08	-1.42	0.25	0.13
		Binding (Tot)	5.23	3.39	9.04	5.31	4.23	10.42
		VDW	-2.55	1.80	0.25	-2.55	1.80	0.25
		EE	4.52	8.08	6.53	4.52	8.08	6.53
1	2	Polar (ePB)	-1.00	9.50	3.89	-0.54	8.28	2.59
		SASA	-0.92	0.63	0.46	-0.46	0.59	0.42
		Binding (Tot)	0.08	3.64	7.61	0.96	1.51	7.03
		VDW	-4.94	1.80	0.33	-4.94	1.80	0.33
		EE	12.34	3.89	2.26	12.34	3.89	2.26
	3	Polar (ePB)	-7.20	3.77	0.21	-7.82	3.56	0.29
		SASA	-1.63	0.33	0.17	-1.00	0.54	0.33
		Binding (Tot)	-1.46	1.51	2.30	-1.42	1.51	2.34
		VDW	-22.05	3.93	0.13	-22.05	3.93	0.13
		EE	10.59	15.56	7.45	10.59	15.56	7.45
	1	Polar (ePB)	10.92	13.31	2.38	8.87	12.68	3.35
		SASA	-3.51	0.17	0.08	-3.72	0.33	0.00
		Binding (Tot)	-4.06	5.02	7.82	-6.32	5.56	8.16
		VDW	-20.79	2.68	0.67	-20.79	2.68	0.67
		EE	23.56	3.35	3.31	23.56	3.35	3.31
2	2	Polar (ePB)	-6.23	2.43	2.09	-10.84	2.30	2.05
		SASA	-3.05	0.25	0.04	-2.93	0.29	0.00
		Binding (Tot)	-6.53	2.76	3.97	-11.00	2.89	3.93
		VDW	-18.03	3.26	0.67	-18.03	3.26	0.67
		EE	39.33	4.10	1.63	39.33	4.10	1.63
	3	Polar (ePB)	-19.16	2.72	0.67	-25.69	2.76	0.63
		SASA	-3.35	0.21	0.08	-3.31	0.33	0.21
		Binding (Tot)	-1.21	3.47	1.88	-7.70	3.64	1.88
		VDW	-5.73	5.40	2.38	-5.73	5.40	2.38
		EE	36.94	13.31	21.34	36.94	13.31	21.34
	1	Polar (ePB)	-27.07	17.15	10.21	-27.11	14.27	7.03
		SASA	-1.76	1.13	0.88	-1.09	1.34	1.00
		Binding (Tot)	2.43	5.56	23.81	3.01	3.56	22.59
		VDW	-9.50	2.47	0.13	-9.50	2.47	0.13
		EE	31.34	12.22	14.69	31.34	12.22	14.69
3	2	Polar (ePB)	-14.06	10.71	4.44	-14.18	12.05	4.69
		SASA	-2.38	0.25	0.04	-2.30	0.46	0.00
		Binding (Tot)	5.40	5.77	15.31	5.31	5.10	15.40
		VDW	-9.41	3.77	0.29	-9.41	3.77	0.29
		EE	17.03	18.37	1.97	17.03	18.37	1.97
	3	Polar (ePB)	-3.01	15.86	1.84	2.68	16.78	2.26
		SASA	-2.64	0.21	0.00	-2.55	0.38	0.08
		Binding (Tot)	2.01	5.15	2.72	7.78	6.32	3.01

Table S4. Complete MM/PBSA and MM/GBSA datasets for serin-ligand interactions. (cont.)

Compound	Triplicate	Energy type	PBSA (kJ/mol)	SD (kJ/mol)	SD prop. (kJ/mol)	GBSA (kJ/mol)	SD (kJ/mol)	SD prop. (kJ/mol)
4	1	VDW	-3 .89	1 .59	0 .50	-3 .89	1 .59	0 .50
		EE	22 .93	11 .25	7 .11	22 .93	11 .25	7 .11
		Polar (ePB)	-18 .41	12 .59	2 .72	-15 .73	12 .05	1 .59
		SASA	-1 .17	0 .63	0 .29	-0 .46	0 .46	0 .21
		Binding (Tot)	-0 .54	1 .76	7 .66	2 .85	0 .84	7 .32
	2	VDW	-10 .84	1 .63	0 .25	-10 .84	1 .63	0 .25
		EE	32 .13	7 .61	1 .38	32 .13	7 .61	1 .38
		Polar (ePB)	-20 .21	5 .82	1 .67	-20 .08	5 .56	0 .67
		SASA	-2 .55	0 .29	0 .17	-1 .97	0 .42	0 .21
		Binding (Tot)	-1 .46	6 .90	2 .18	-0 .75	3 .64	1 .55
	3	VDW	-8 .37	3 .01	0 .04	-8 .37	3 .01	0 .04
		EE	14 .06	6 .53	4 .64	14 .06	6 .53	4 .64
		Polar (ePB)	-5 .48	6 .15	1 .97	-3 .60	5 .23	1 .63
		SASA	-2 .18	0 .50	0 .00	-1 .72	0 .59	0 .04
		Binding (Tot)	-1 .97	2 .76	5 .06	0 .38	2 .01	4 .94
5	1	VDW	-11 .00	2 .59	0 .42	-11 .00	2 .59	0 .42
		EE	43 .81	4 .94	2 .64	43 .81	4 .94	2 .64
		Polar (ePB)	-35 .35	4 .18	1 .26	-30 .46	4 .10	1 .42
		SASA	-2 .64	0 .17	0 .00	-2 .34	0 .25	0 .04
		Binding (Tot)	-5 .19	2 .55	2 .97	0 .00	2 .85	3 .05
	2	VDW	-20 .75	4 .48	1 .09	-20 .75	4 .48	1 .09
		EE	-3 .26	15 .36	4 .94	-3 .26	15 .36	4 .94
		Polar (ePB)	25 .15	14 .64	4 .23	20 .59	14 .14	1 .76
		SASA	-3 .97	0 .38	0 .04	-4 .39	0 .79	0 .33
		Binding (Tot)	-2 .85	7 .11	6 .57	-7 .87	5 .82	5 .36
	3	VDW	-10 .25	1 .97	0 .04	-10 .25	1 .97	0 .04
		EE	6 .44	2 .80	5 .19	6 .44	2 .80	5 .19
		Polar (ePB)	1 .55	2 .93	2 .01	2 .68	2 .76	2 .22
		SASA	-2 .76	0 .25	0 .04	-2 .18	0 .38	0 .00
		Binding (Tot)	-5 .06	1 .80	5 .56	-3 .31	1 .76	5 .65
6	1	VDW	-6 .82	2 .76	0 .71	-6 .82	2 .76	0 .71
		EE	2 .89	12 .97	7 .36	2 .89	12 .97	7 .36
		Polar (ePB)	2 .93	13 .93	0 .50	6 .65	13 .18	0 .38
		SASA	-2 .01	0 .54	0 .04	-0 .96	0 .67	0 .13
		Binding (Tot)	-3 .01	1 .88	7 .45	1 .76	1 .67	7 .41
	2	VDW	-27 .95	4 .69	0 .54	-27 .95	4 .69	0 .54
		EE	-62 .26	16 .36	14 .94	-62 .26	16 .36	14 .94
		Polar (ePB)	88 .41	12 .93	9 .16	81 .63	13 .26	8 .08
		SASA	-5 .44	0 .33	0 .13	-6 .36	0 .50	0 .17
		Binding (Tot)	-7 .24	6 .07	17 .53	-14 .94	4 .98	16 .99
	3	VDW	-6 .57	1 .30	0 .00	-6 .57	1 .30	0 .00
		EE	-3 .14	9 .08	20 .21	-3 .14	9 .08	20 .21
		Polar (ePB)	9 .08	9 .37	2 .68	11 .46	8 .37	1 .34
		SASA	-1 .88	0 .33	0 .08	-1 .17	0 .38	0 .04
		Binding (Tot)	-2 .47	1 .76	20 .42	0 .63	1 .55	20 .25

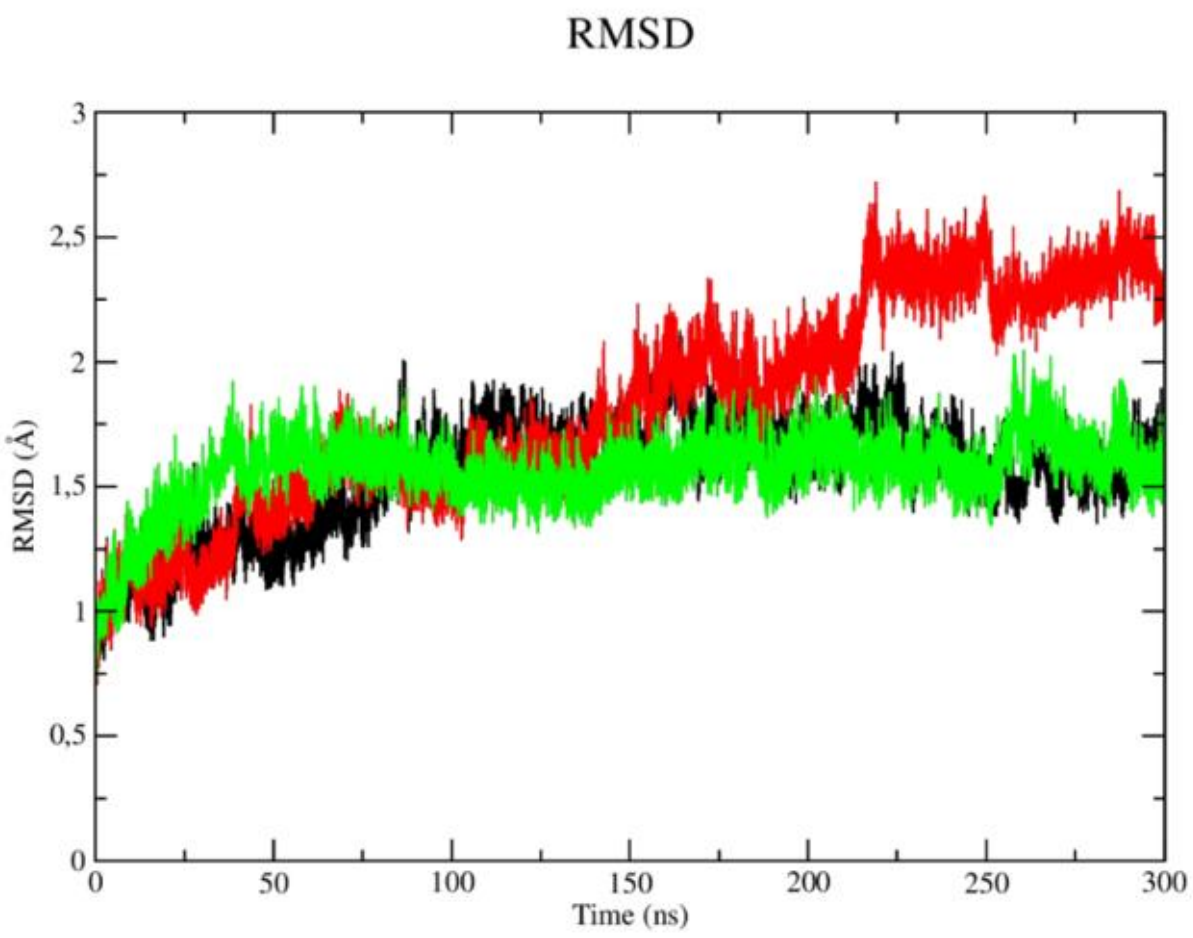

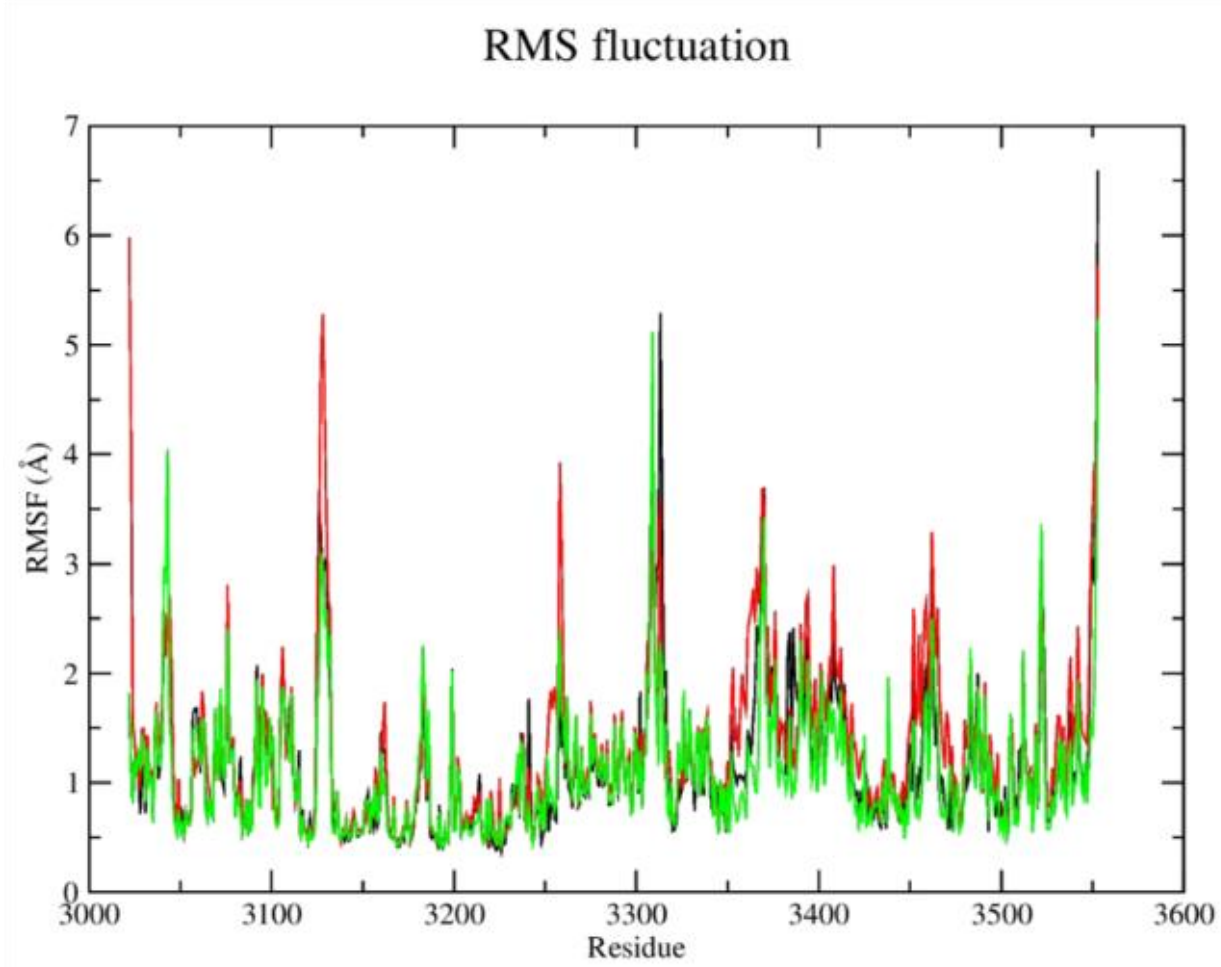

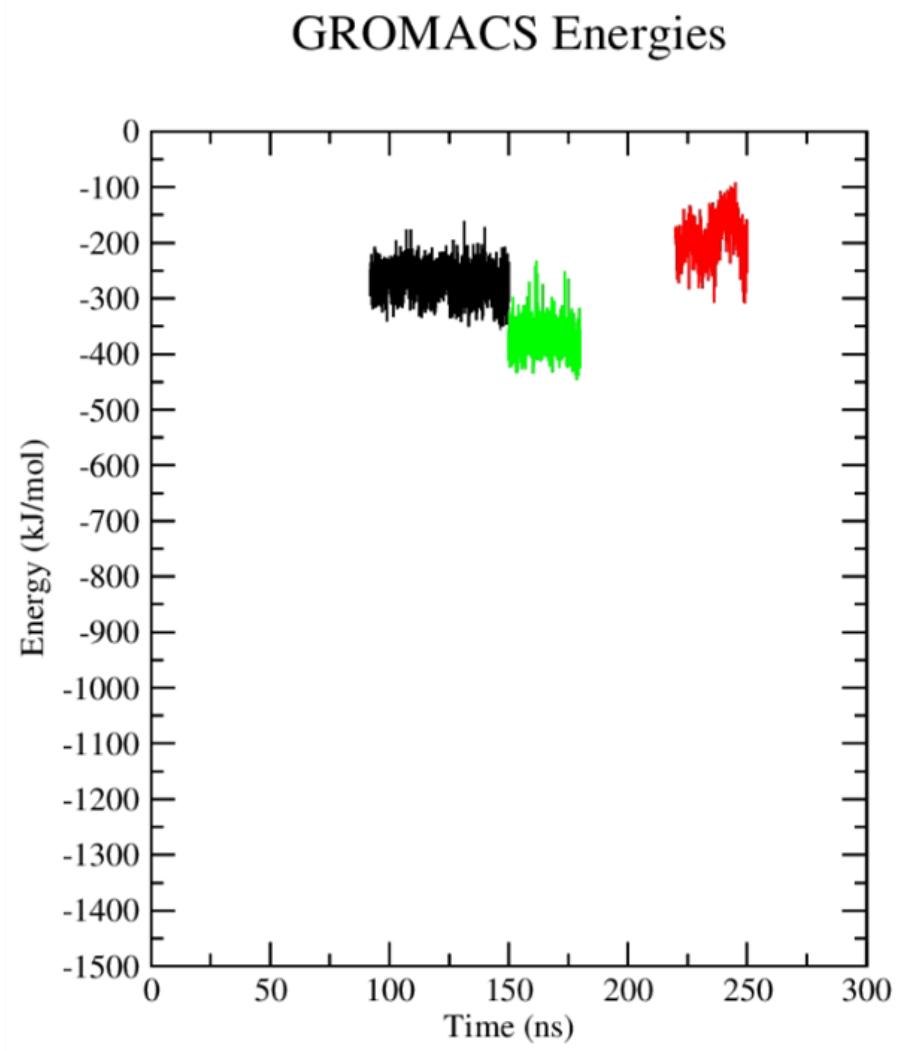

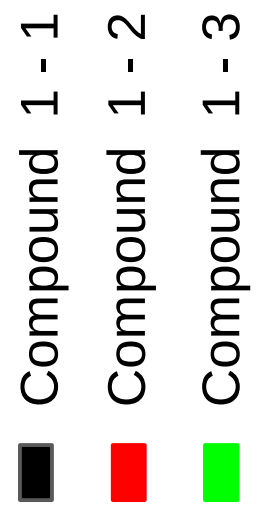

Energy	Average	Err.Est.	RMSD	Tot-Drift	
Coul-SR:LIG-Protein	-109.488	4	26.1997	24.0967	(kJ/mol)
LJ-SR:LIG-Protein	-148.706	0.83	11.8589	1.96998	(kJ/mol)
Total	-258.193	4.6	--	26.0666	(kJ/mol)

Energy	Average	Err.Est.	RMSD	Tot-Drift	
Coul-SR:LIG-Protein	-69.9623	6.9	37.6161	-31.1118	(kJ/mol)
LJ-SR:LIG-Protein	-147.383	4.3	14.751	-26.6666	(kJ/mol)
Total	-217.346	11	--	-57.7784	(kJ/mol)

Energy	Average	Err.Est.	RMSD	Tot-Drift	
Coul-SR:LIG-Protein	-179.784	2.5	31.7554	-15.8876	(kJ/mol)
LJ-SR:LIG-Protein	-189.585	1.1	13.2472	-6.06086	(kJ/mol)
Total	-369.369	3.2	--	-21.9485	(kJ/mol)

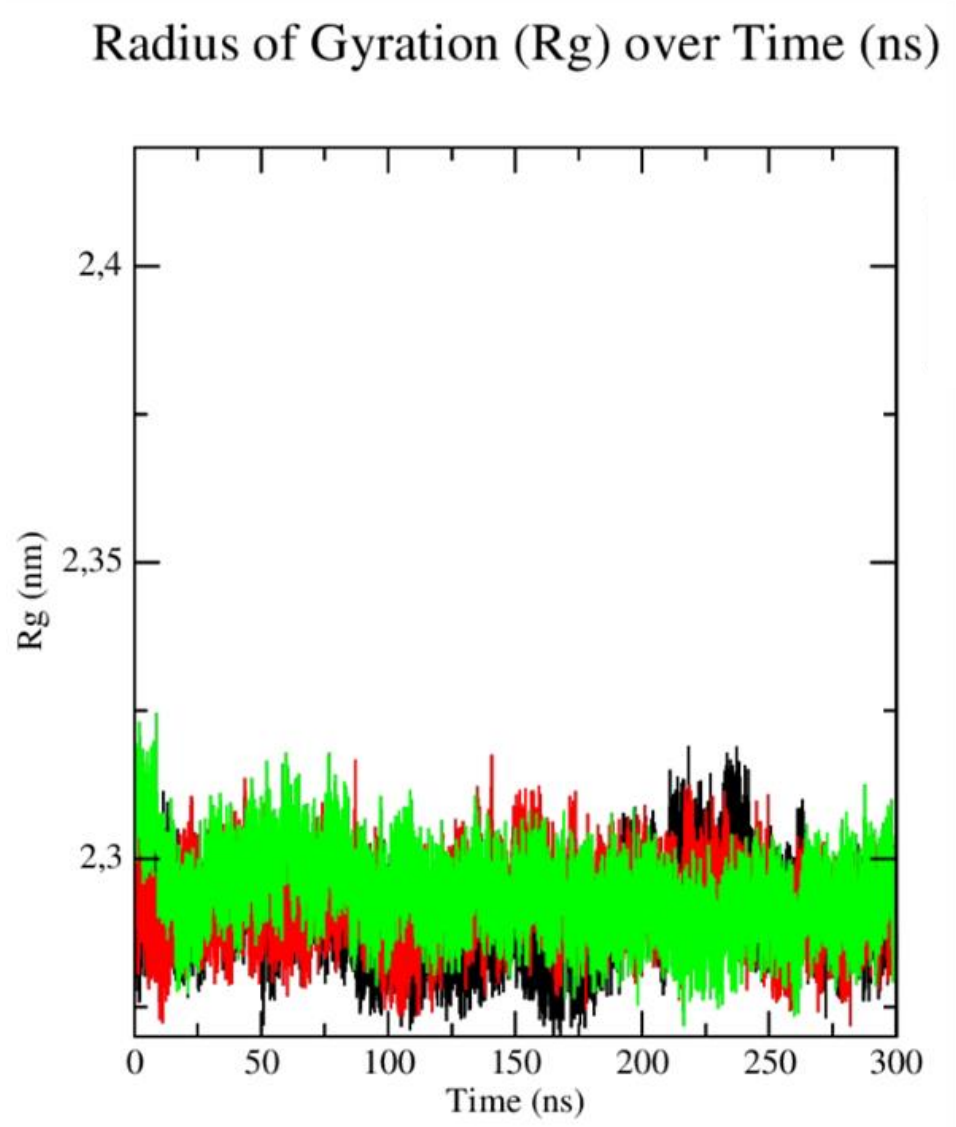

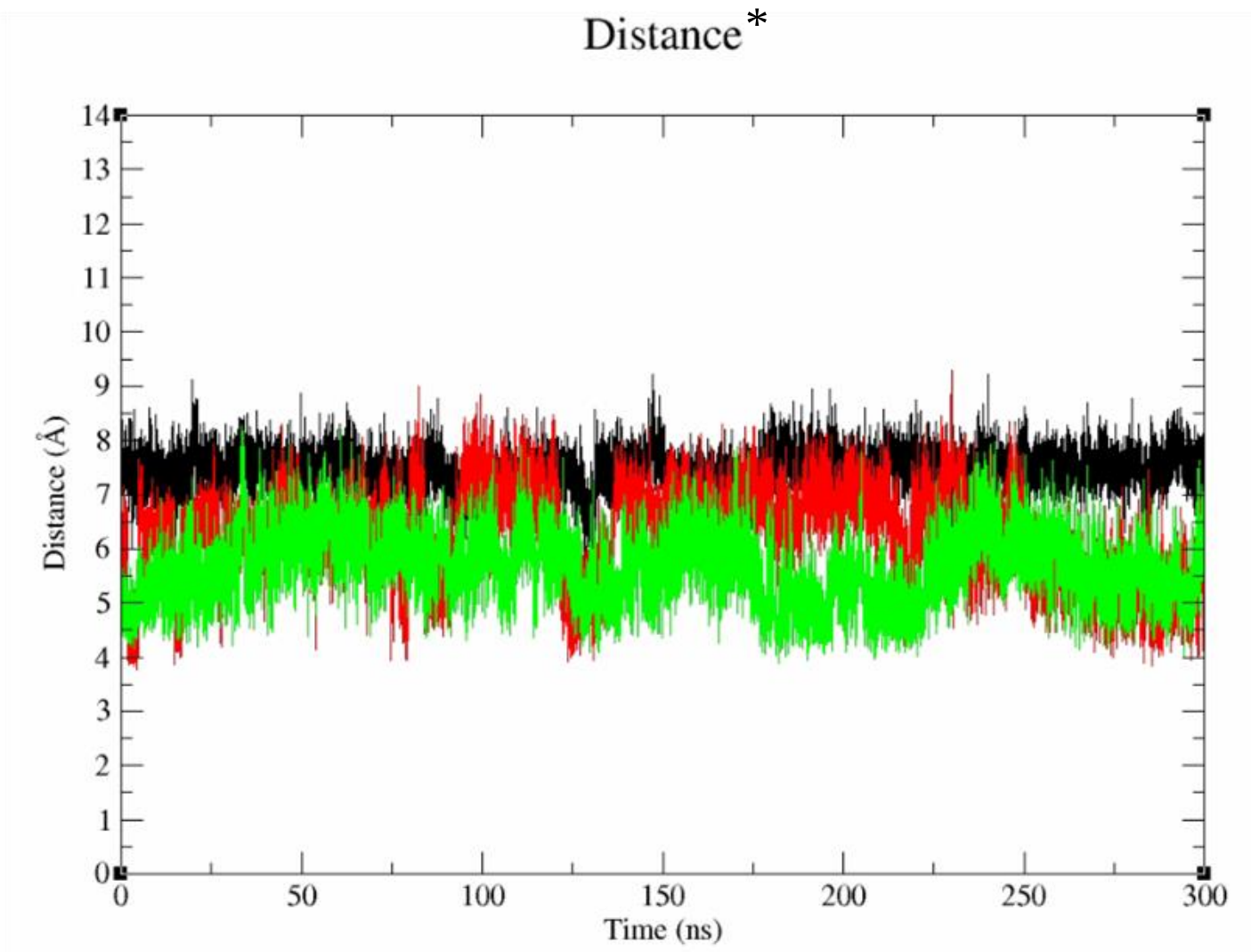

*this graph considers the distances between the closest carbonyl group of the compound to the hydroxyl group of the Ser221

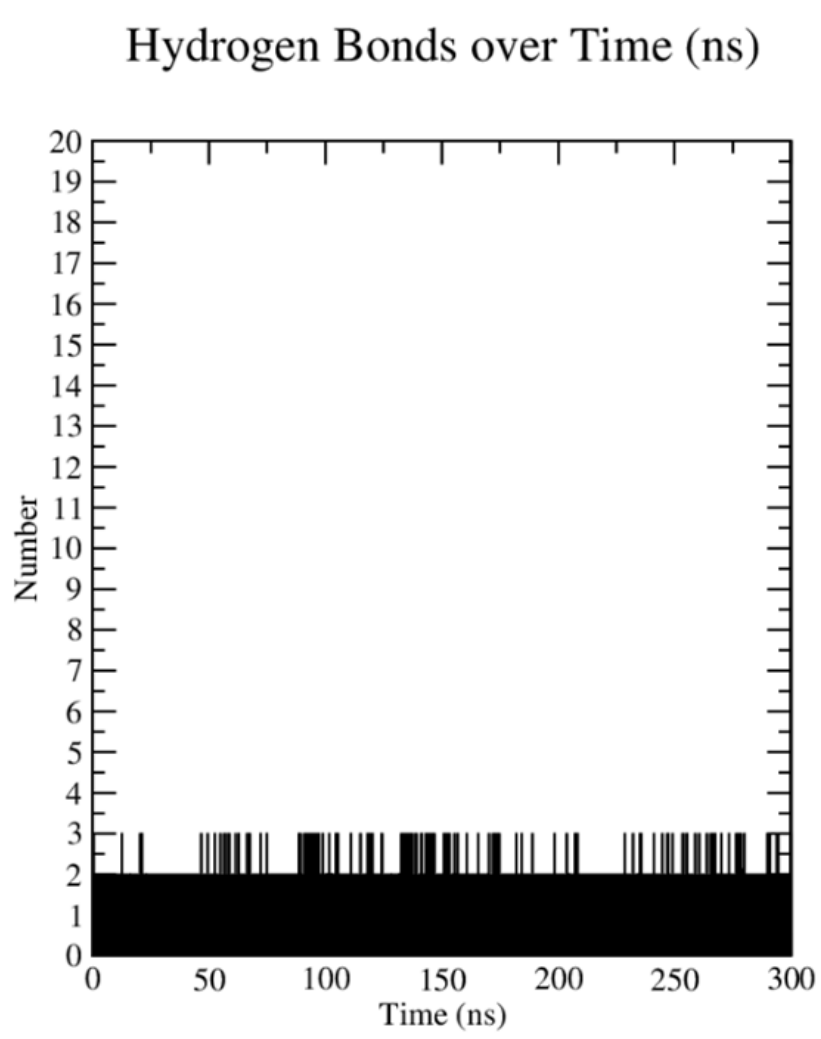

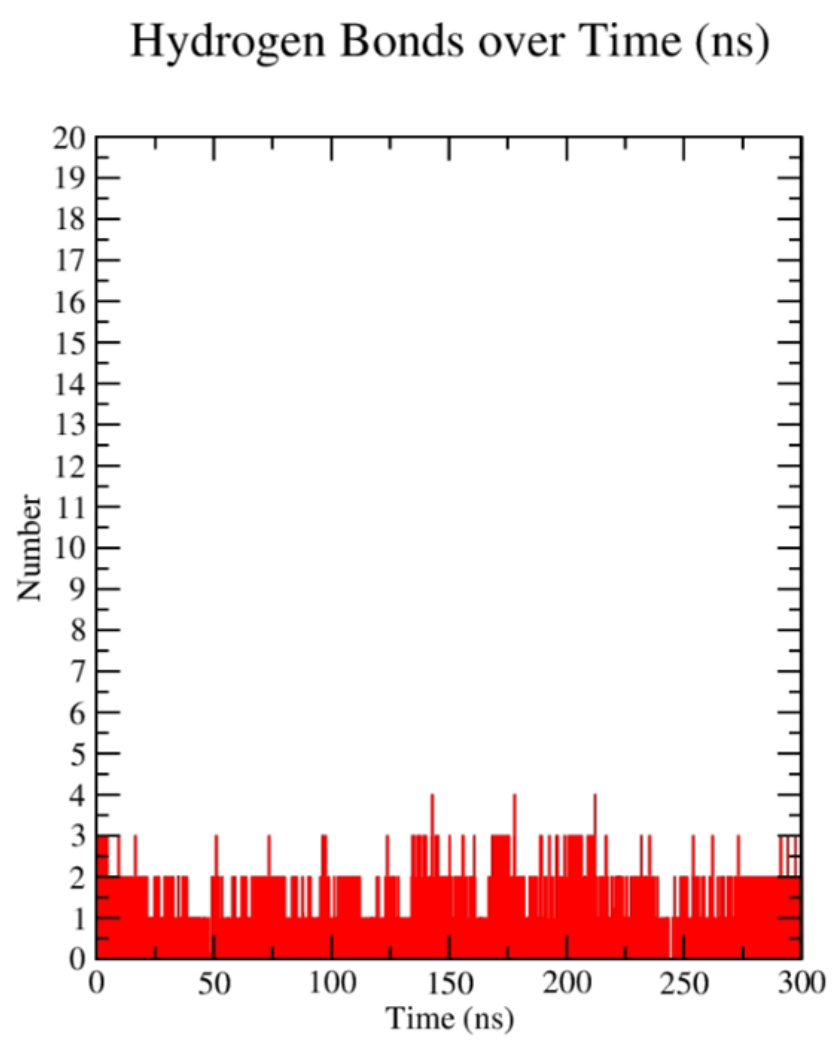

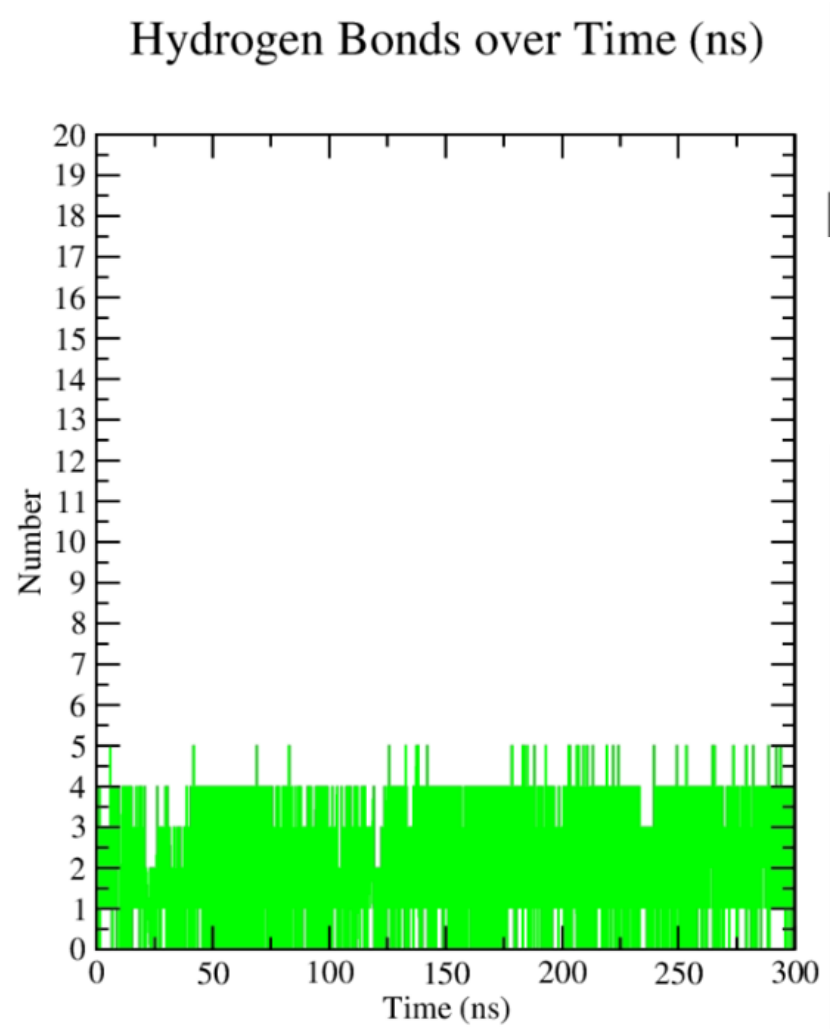

Figure S1. Molecular dynamic results for Compound 1.

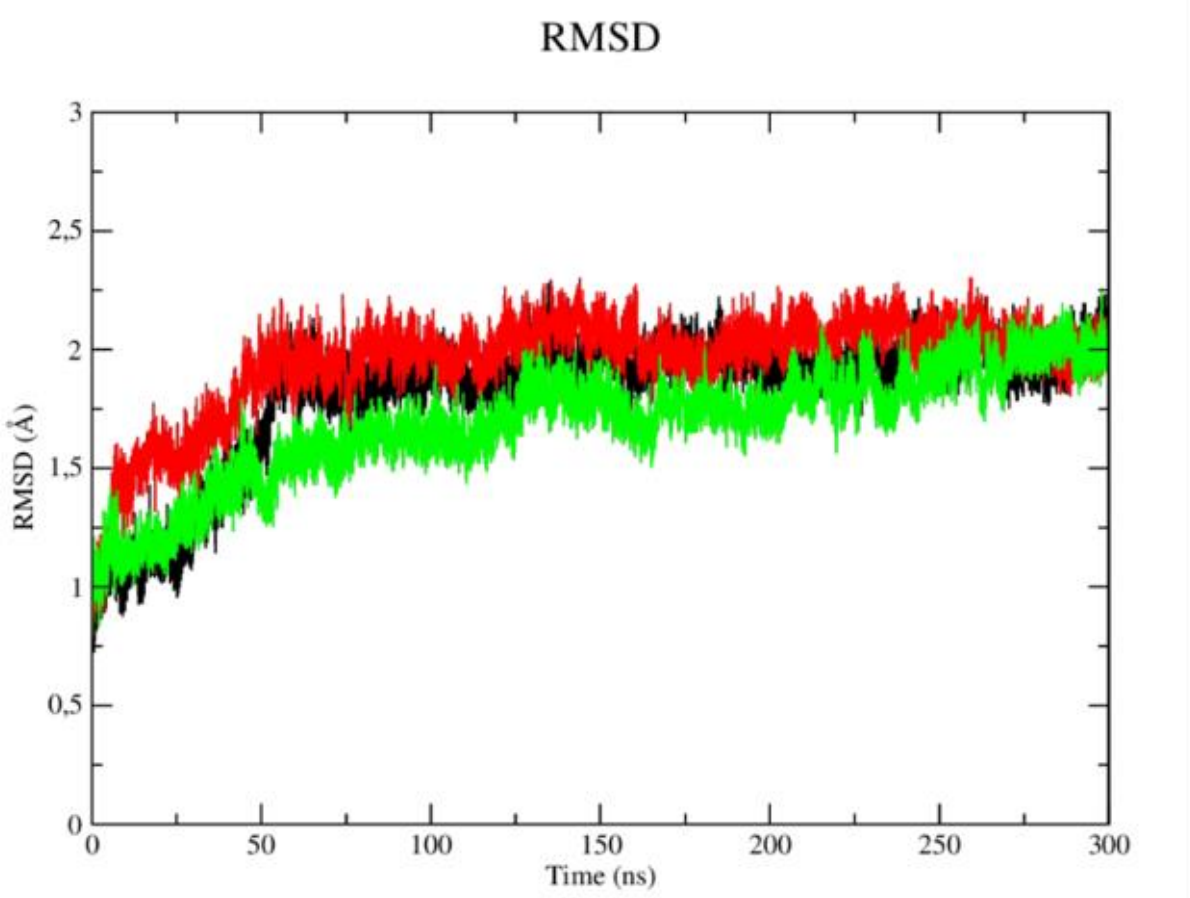

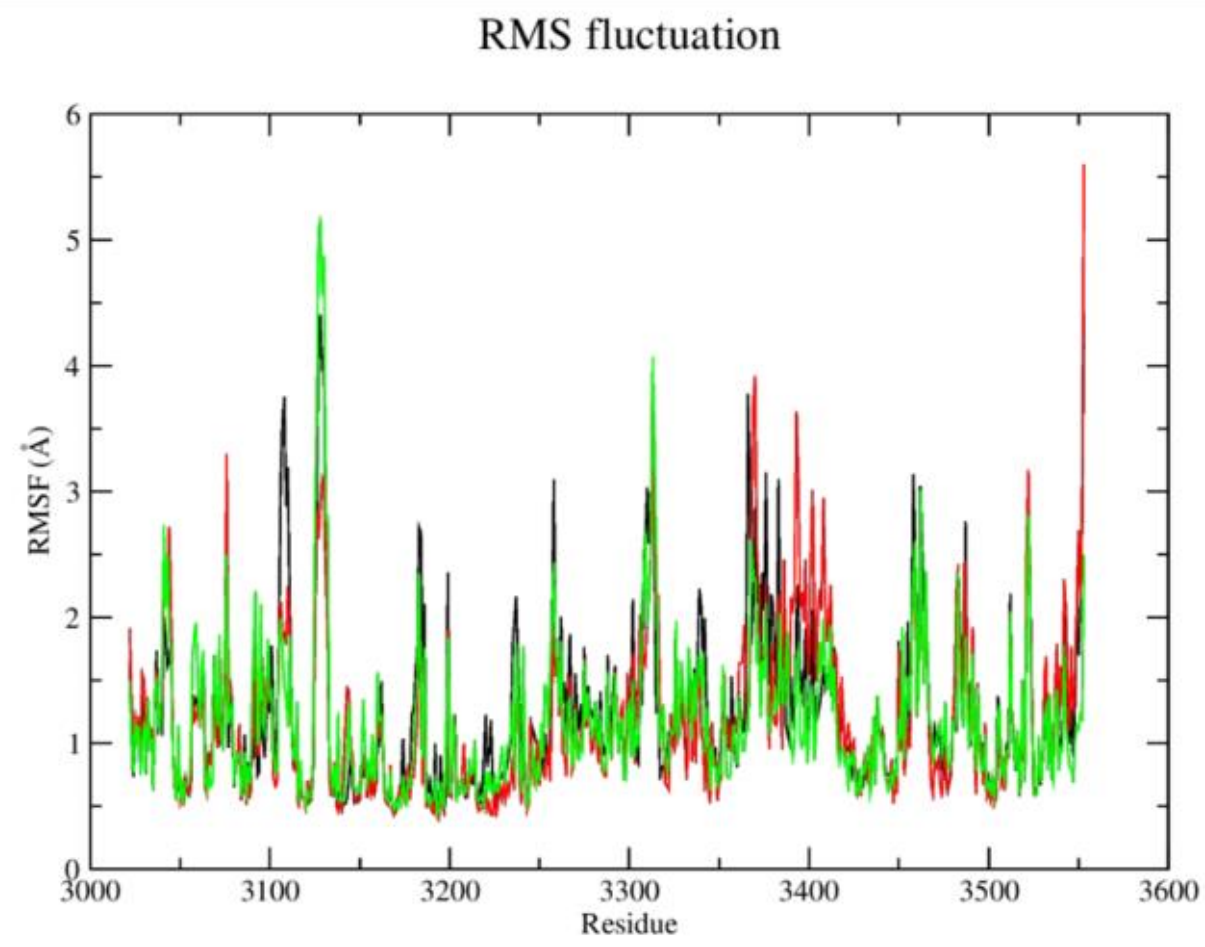

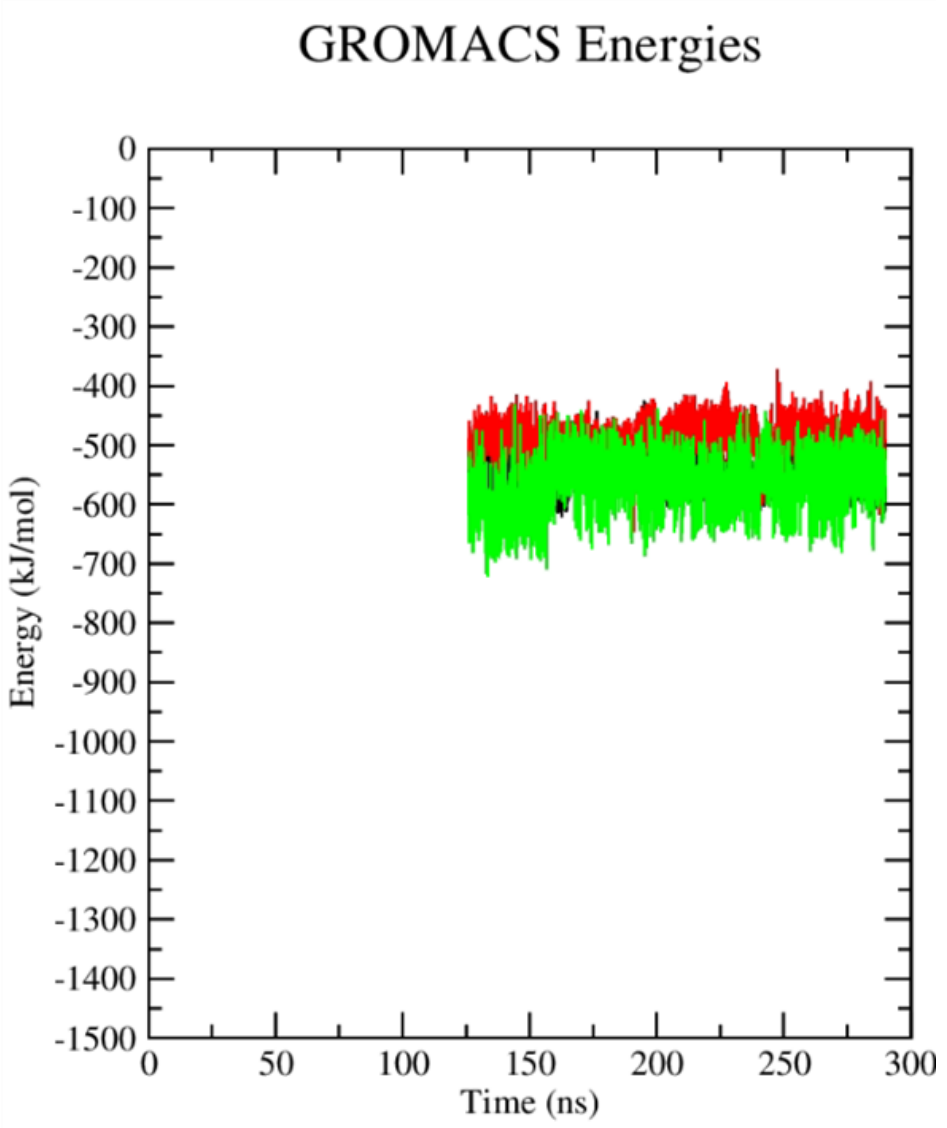

**Energy**	Average	Err.Est.	RMSD	Tot-Drift
Coul-SR:LIG-Protein	-171.997	6.2	23.1968	-38.5268 (kJ/mol)
LJ-SR:LIG-Protein	-366.185	3.3	17.7425	-2.81376 (kJ/mol)
Total	-538.182	6.5	--	-41.3406 (kJ/mol)

**Energy**	Average	Err.Est.	RMSD	Tot-Drift
Coul-SR:LIG-Protein	-183.642	3.1	24.2494	2.7622 (kJ/mol)
LJ-SR:LIG-Protein	-310.217	3.2	17.6546	12.6443 (kJ/mol)
Total	-493.859	3.1	--	15.4065 (kJ/mol)

**Energy**	Average	Err.Est.	RMSD	Tot-Drift
Coul-SR:LIG-Protein	-233.579	9.6	45.1389	56.0466 (kJ/mol)
LJ-SR:LIG-Protein	-343.45	1.7	16.3275	-4.05682 (kJ/mol)
Total	-577.029	9.6	--	51.9898 (kJ/mol)

Compound 2 - 1
Compound 2 - 2
Compound 2 - 3

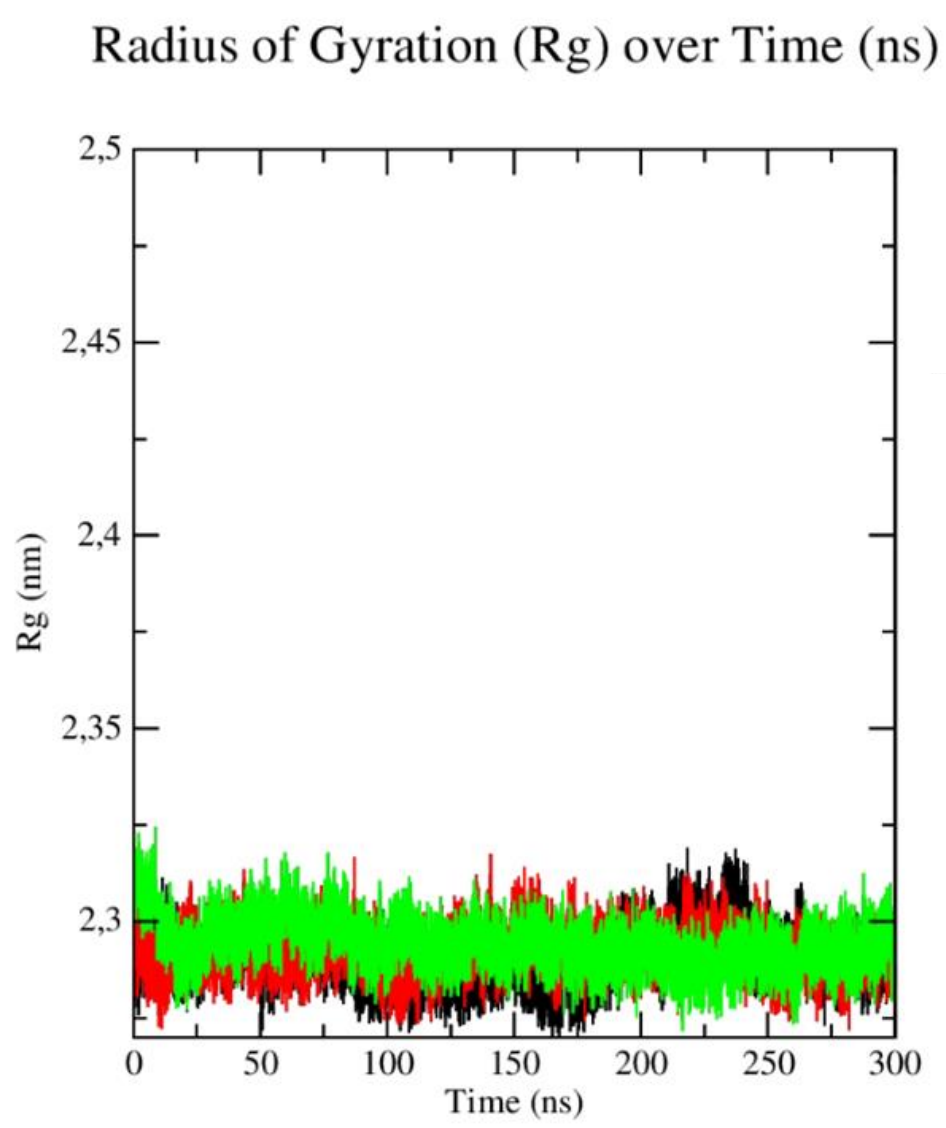

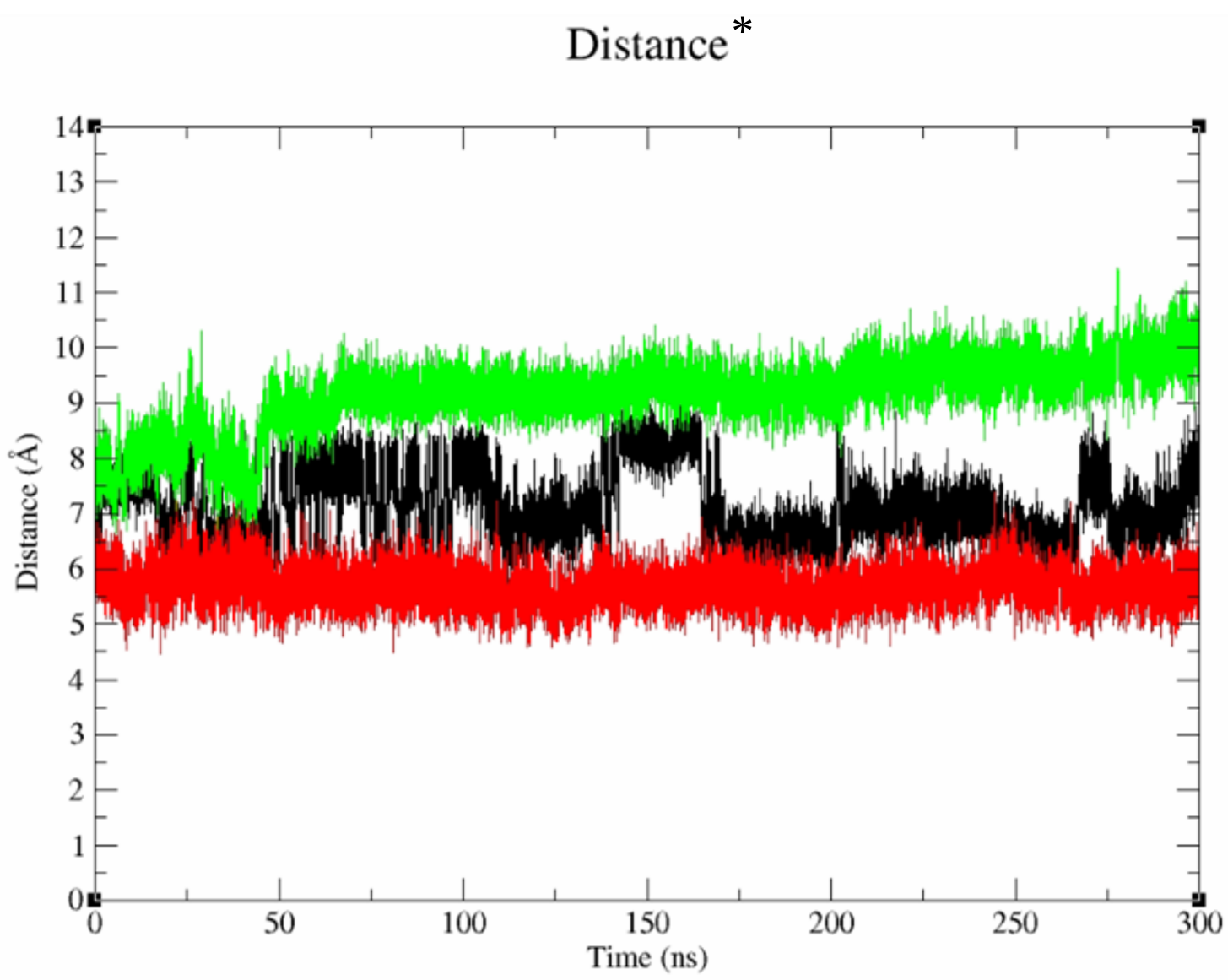

*this graph considers the distances between the closest carbonyl group of the compound to the hydroxyl group of the Ser221

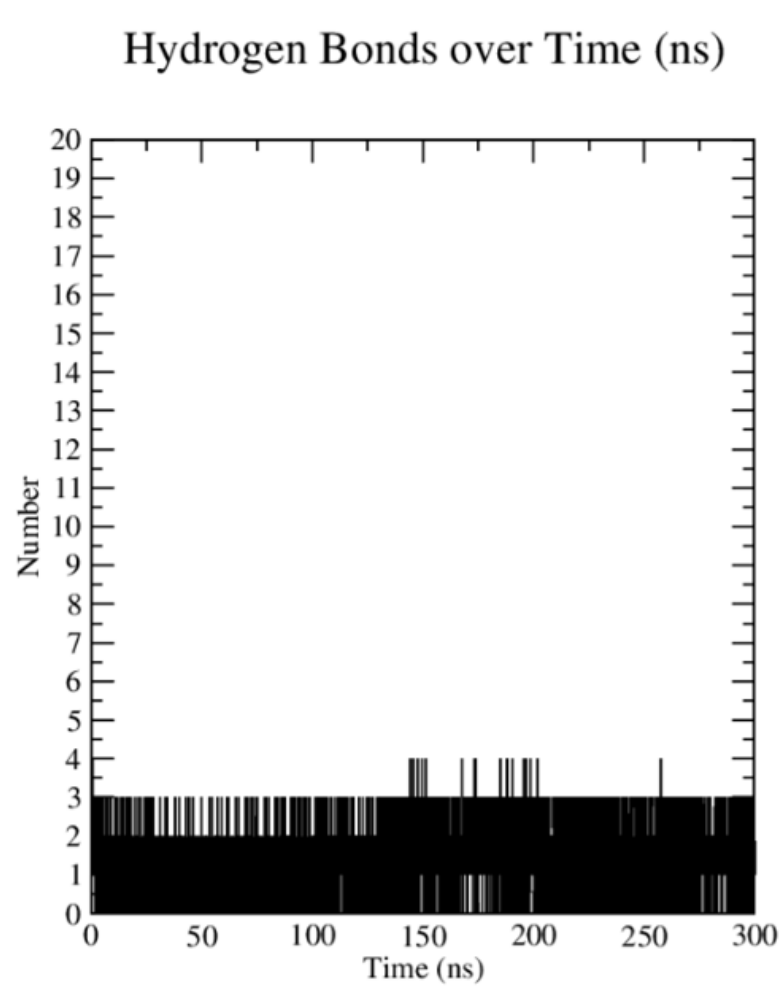

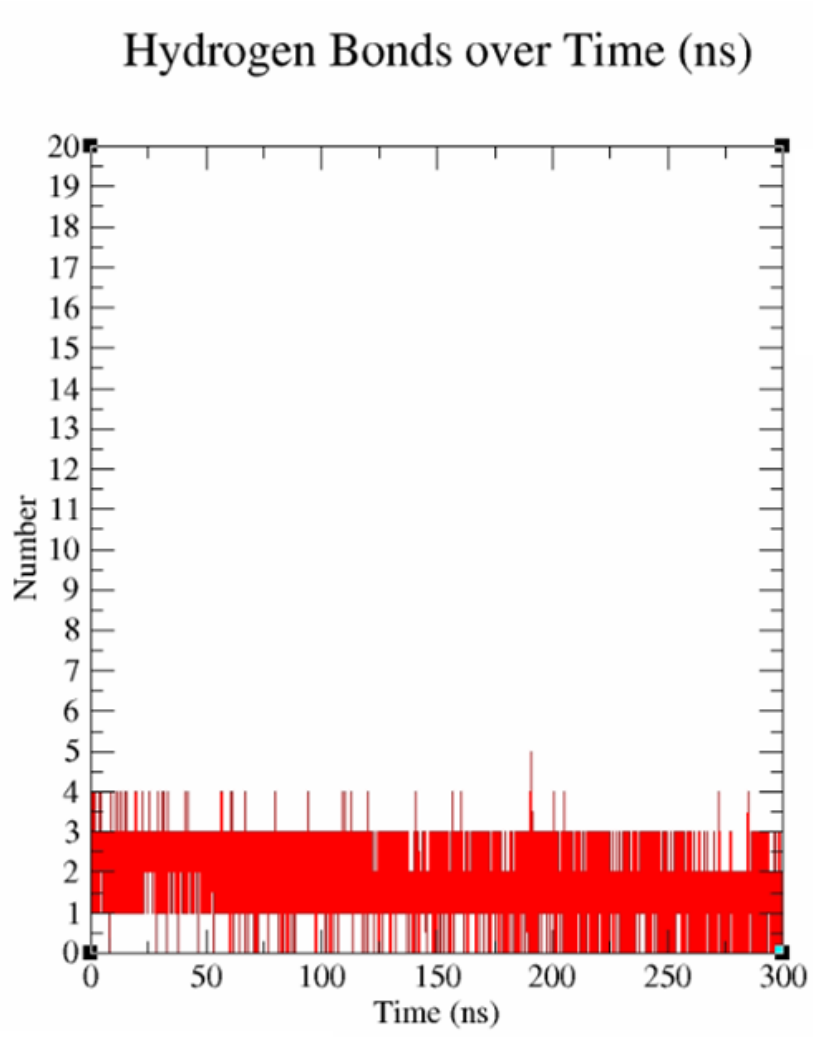

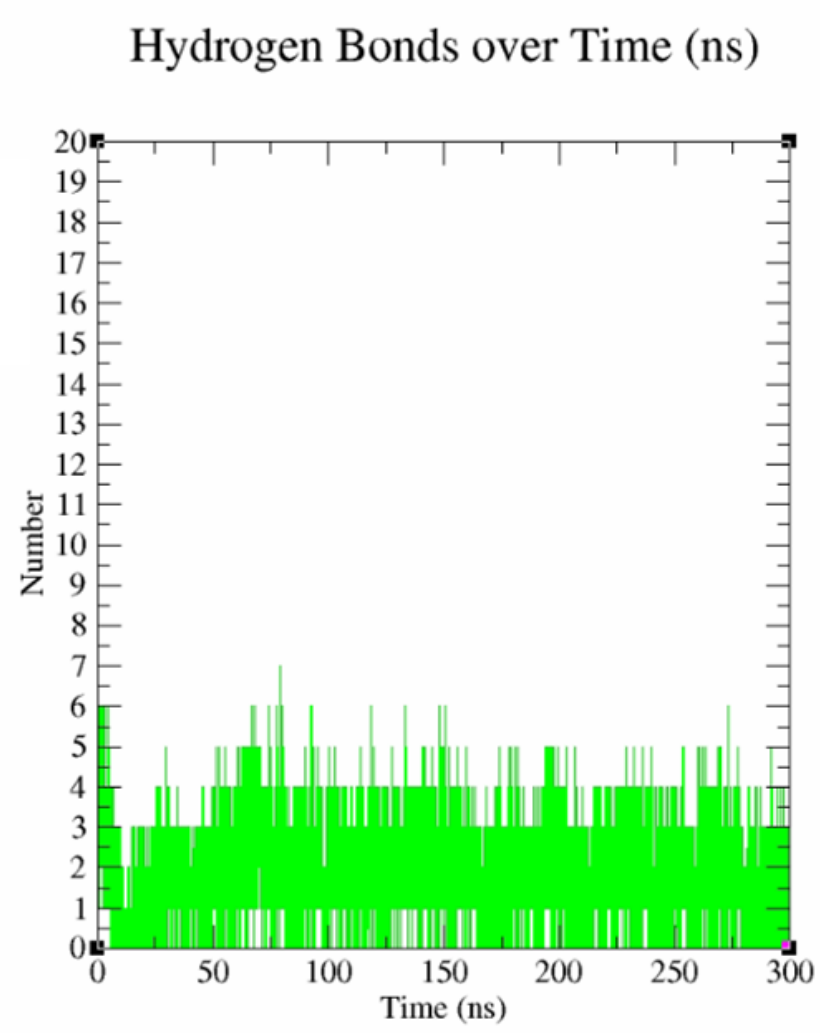

Figure S2. Molecular dynamic results for Compound 2.

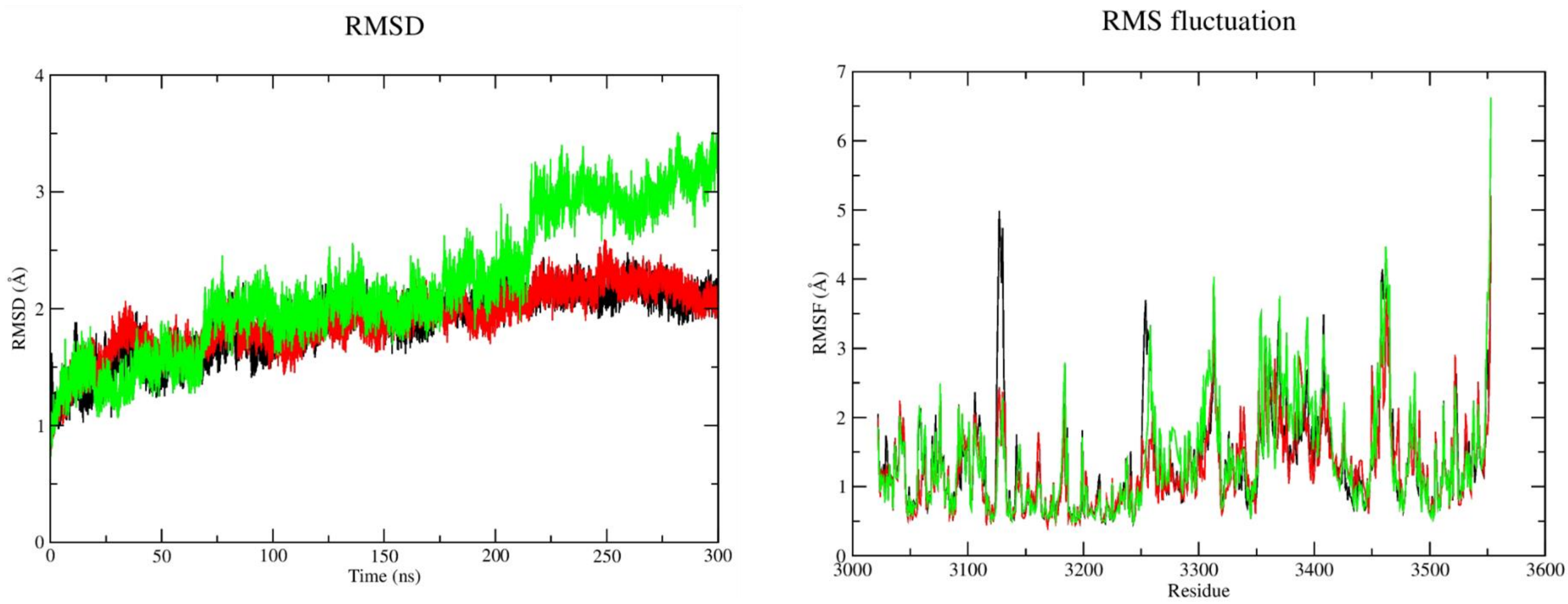

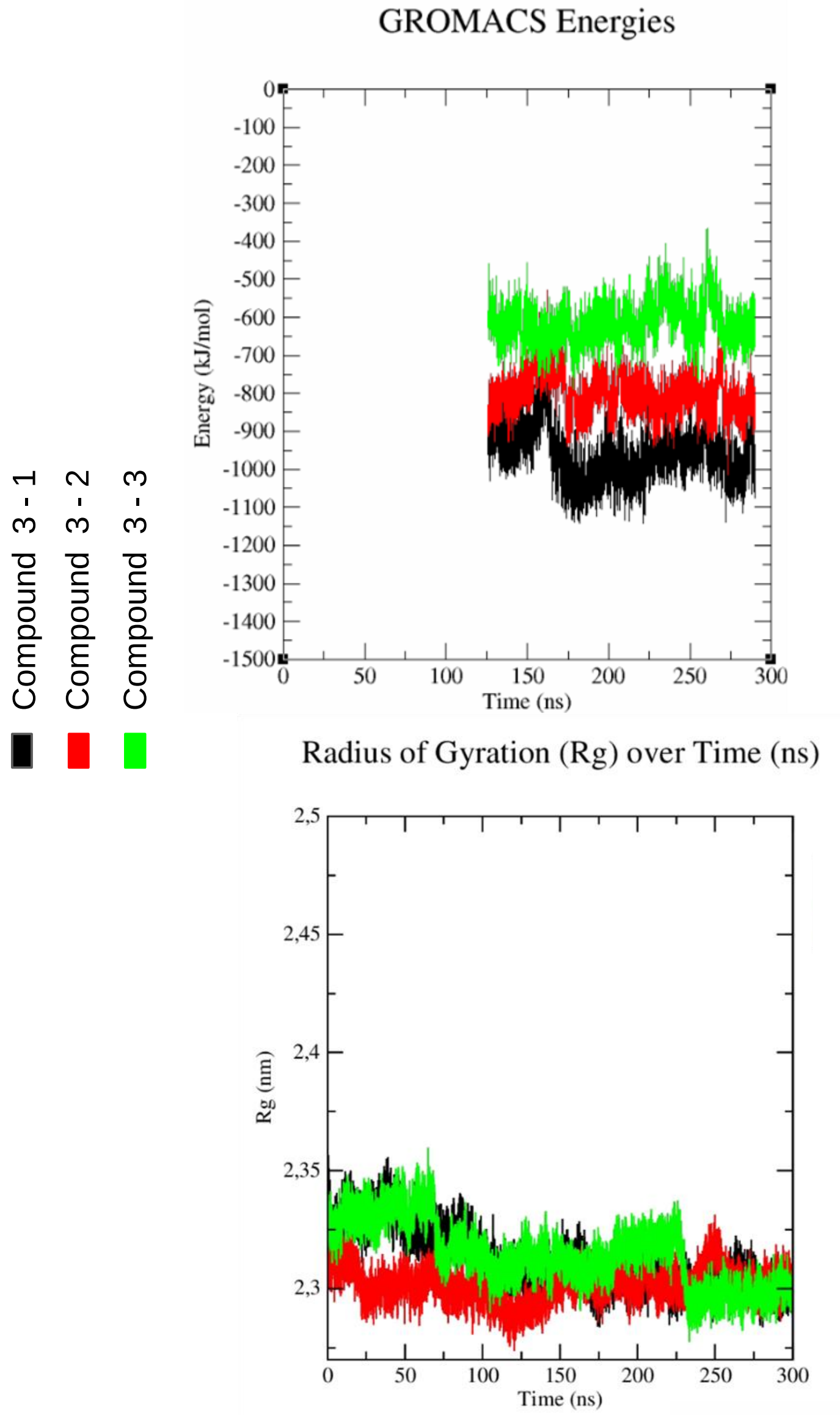

Energy	Average	Err.Est.	RMSD	Tot-Drift
Coul-SR:LIG-Protein	-598.929	11	64.2662	-66.6483 (kJ/mol)
LJ-SR:LIG-Protein	-330.898	13	35.8515	-87.3789 (kJ/mol)
Total	-929.828	23	--	-154.027 (kJ/mol)

Energy	Average	Err.Est.	RMSD	Tot-Drift
Coul-SR:LIG-Protein	-486.867	10	56.5799	-64.494 (kJ/mol)
LJ-SR:LIG-Protein	-300.658	3	22.2447	-0.2061 (kJ/mol)
Total	-787.525	11	--	-64.7001 (kJ/mol)

Energy	Average	Err.Est.	RMSD	Tot-Drift
Coul-SR:LIG-Protein	-308.562	3.2	41.456	-15.9627 (kJ/mol)
LJ-SR:LIG-Protein	-314.904	2.6	20.978	-11.8405 (kJ/mol)
Total	-623.466	5.4	--	-27.8032 (kJ/mol)

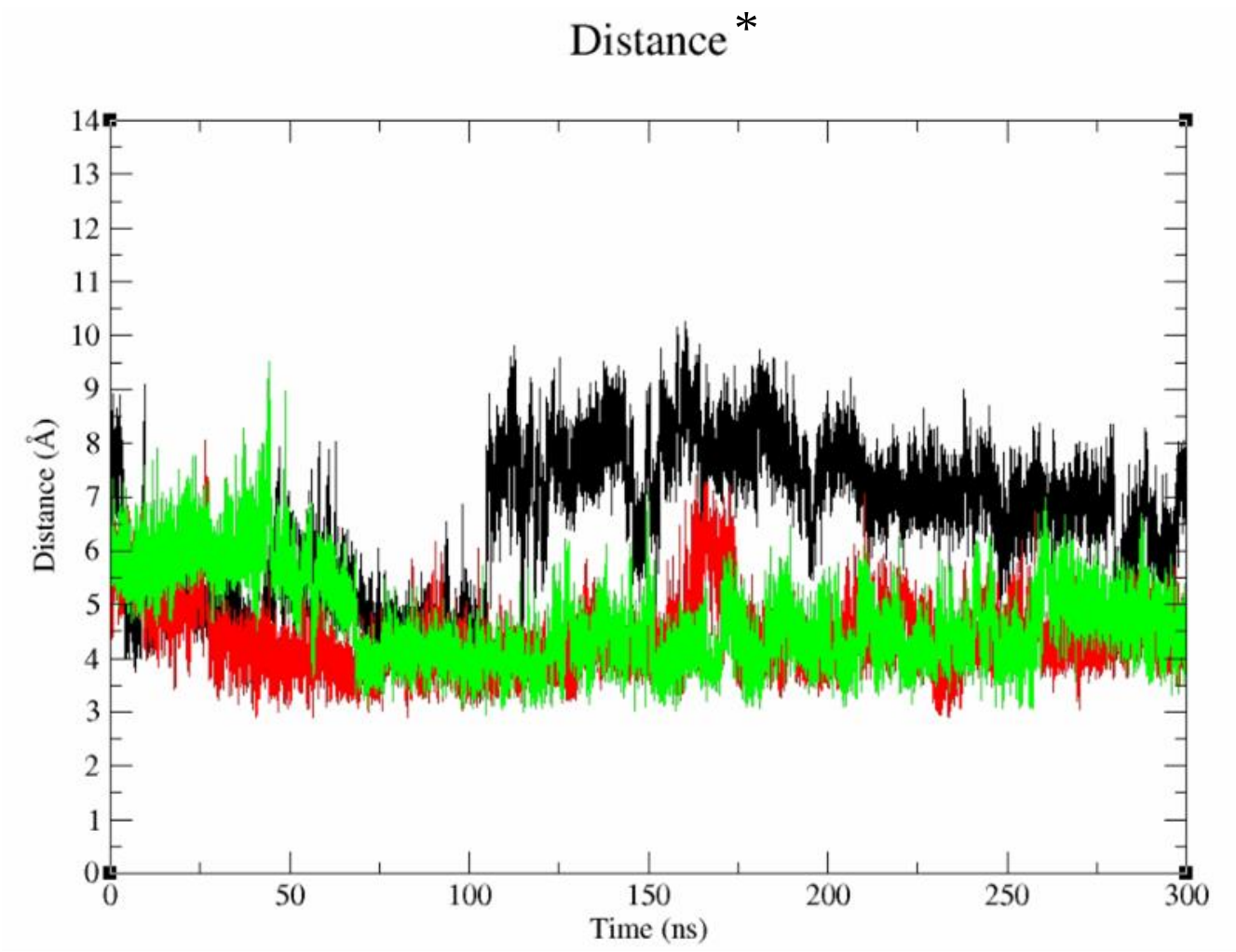

*this graph considers the distances between the closest carbonyl group of the compound to the hydroxyl group of the Ser221

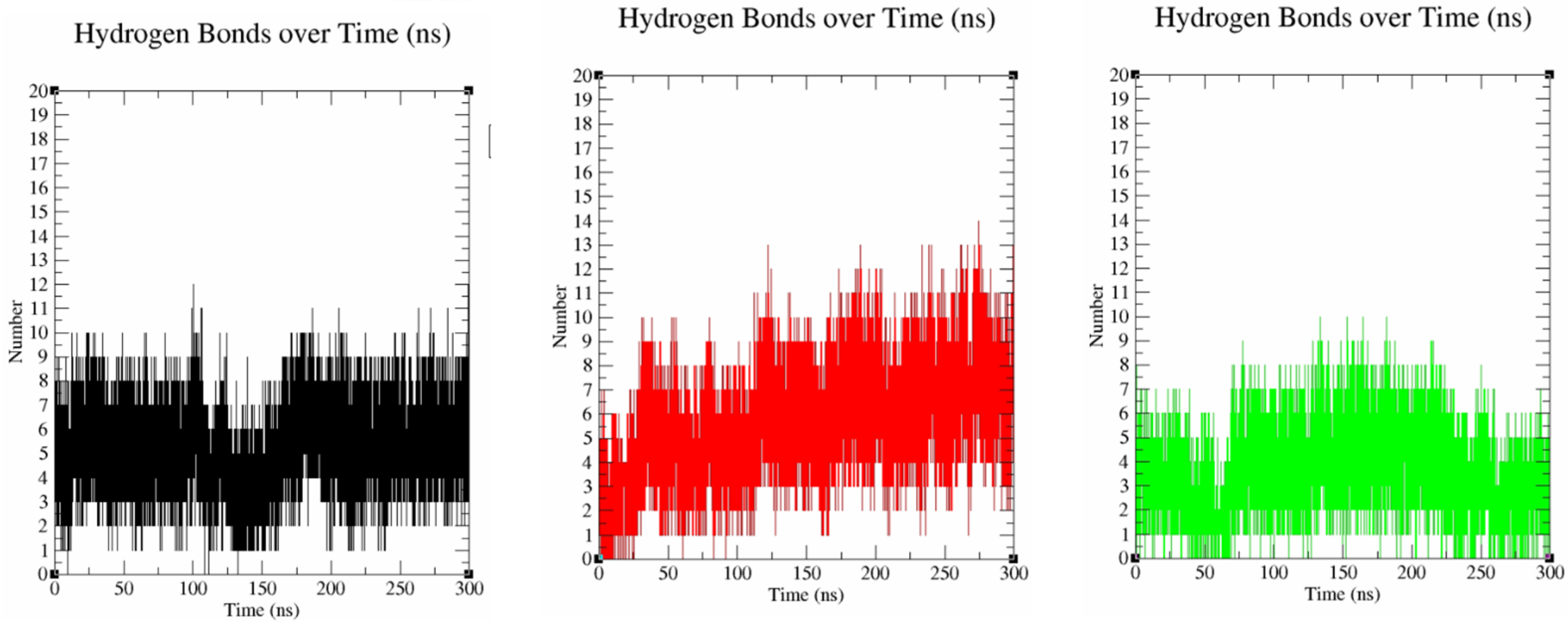

Figure S3. Molecular dynamic results for Compound 3.

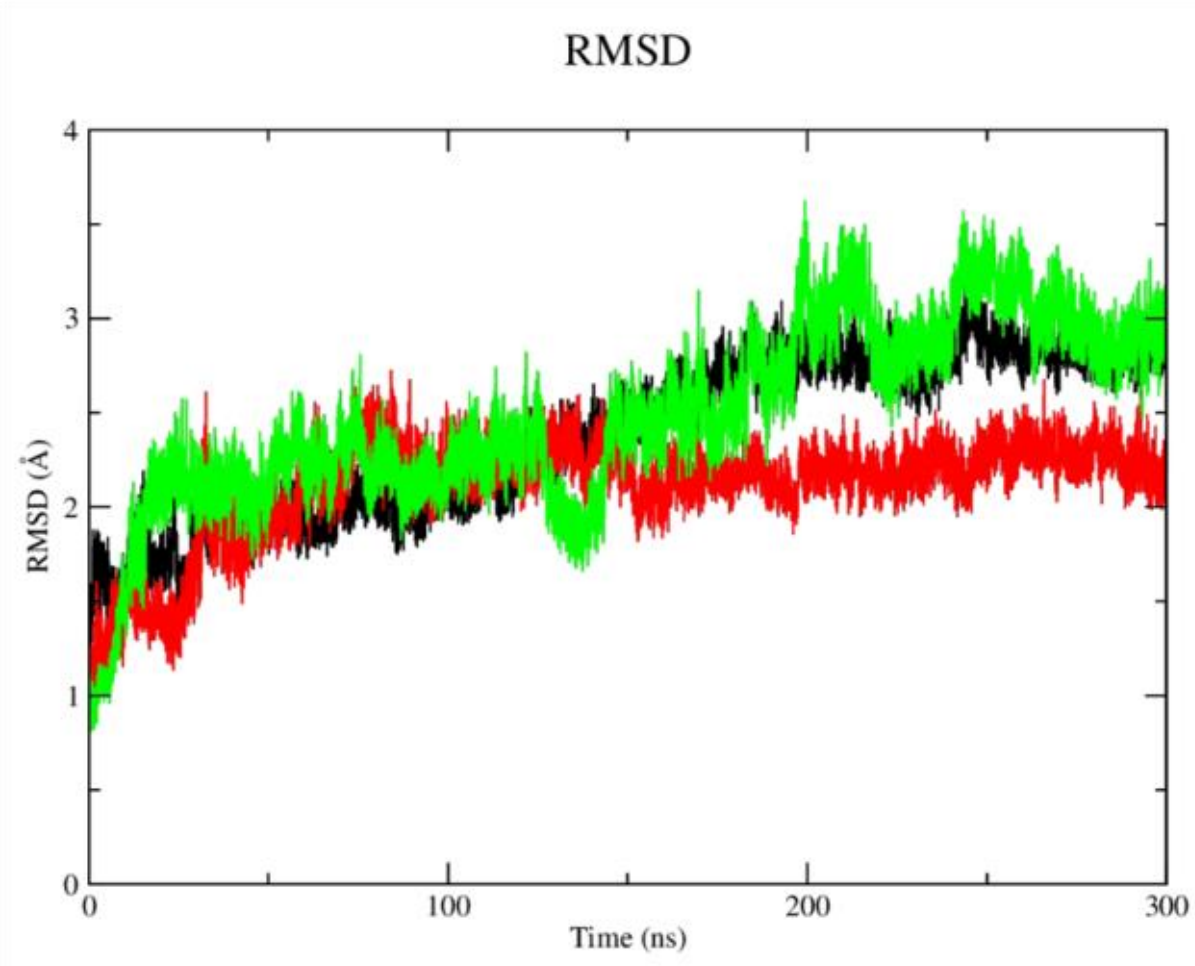

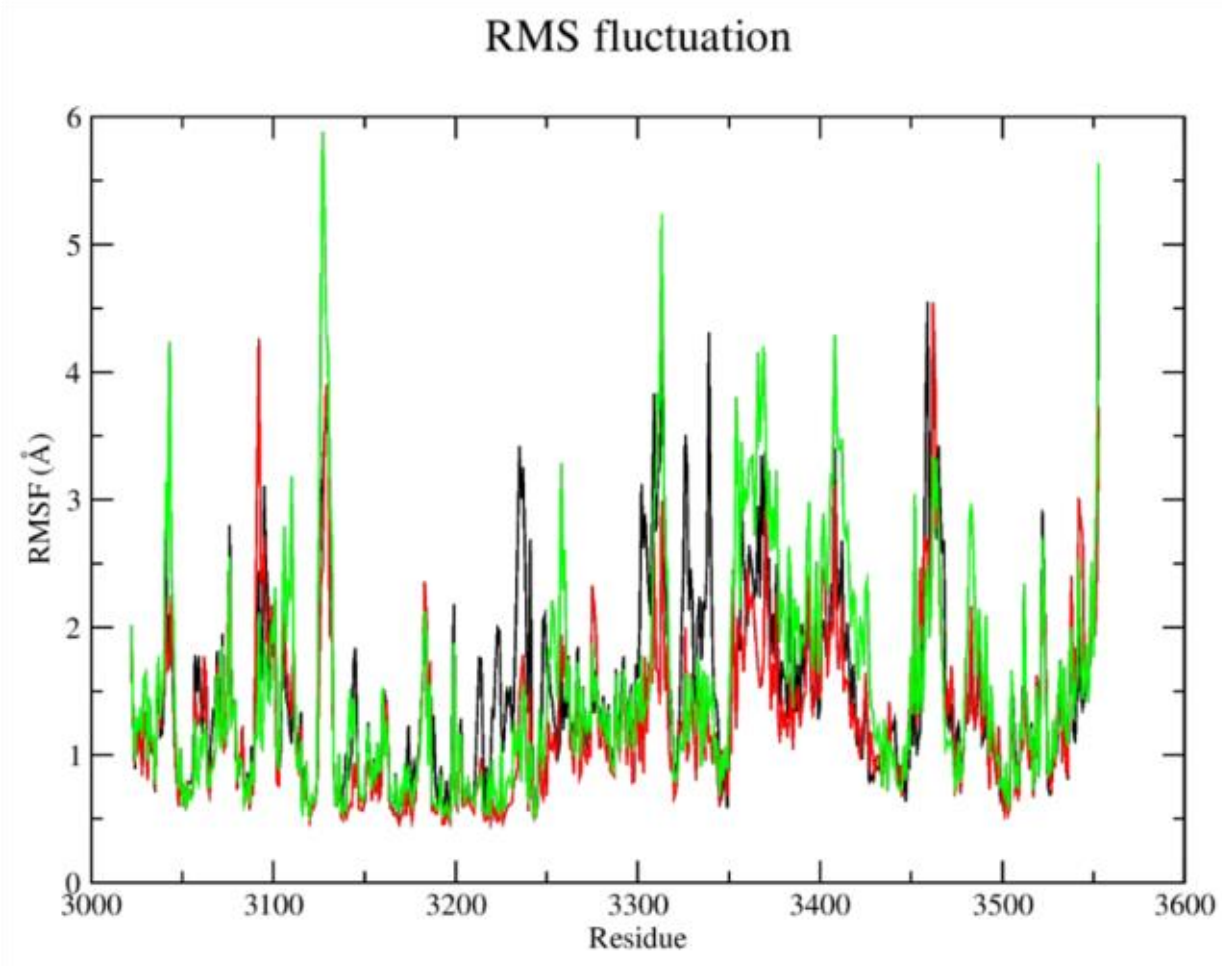

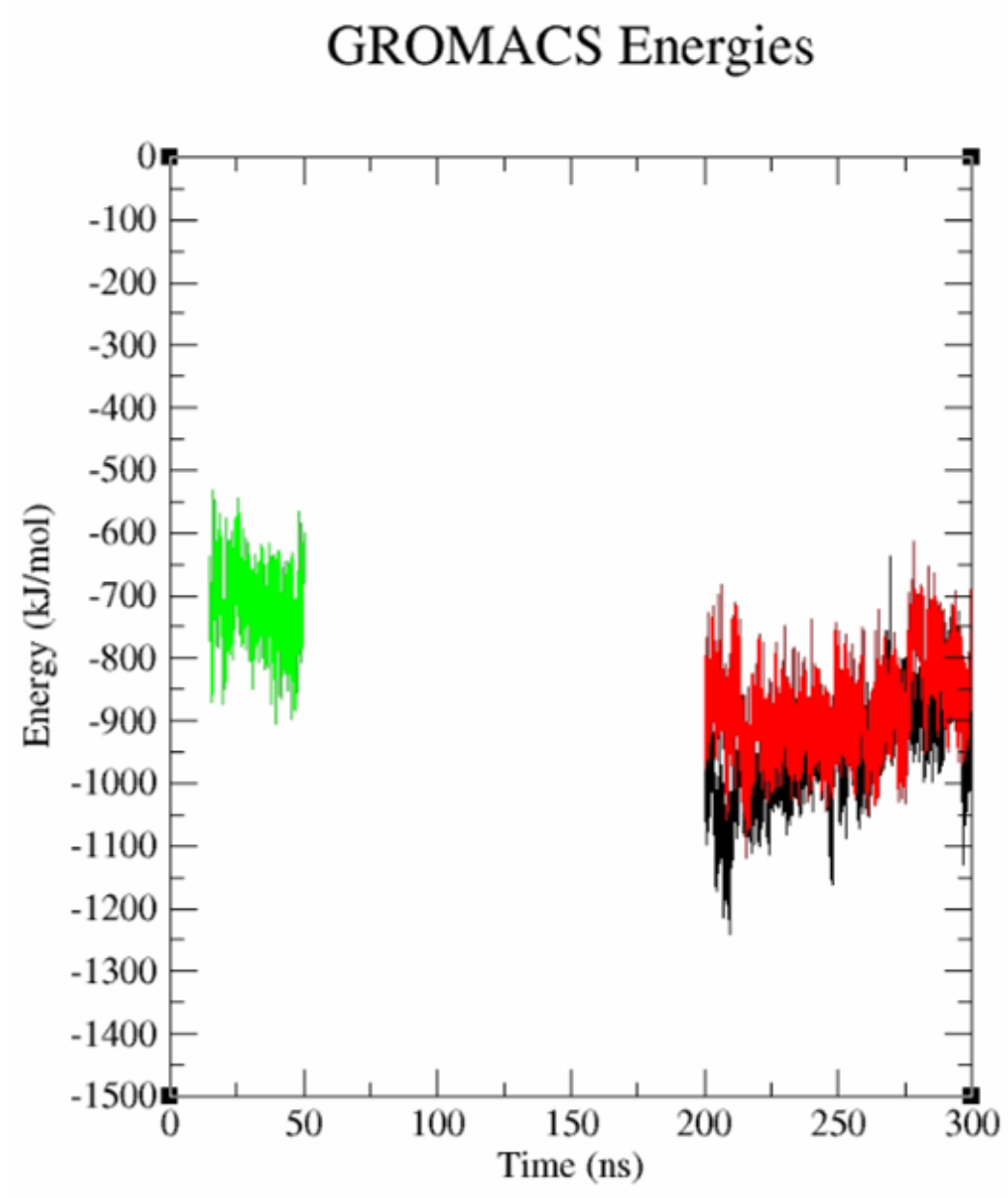

**Energy**	Average	Err.Est.	RMSD	Tot-Drift	
Coul-SR:LIG-Protein	-556.192	24	69.7223	167.034	(kJ/mol)
LJ-SR:LIG-Protein	-391.638	3.3	25.0124	17.7893	(kJ/mol)
Total	-947.83	27	--	184.824	(kJ/mol)

**Energy**	Average	Err.Est.	RMSD	Tot-Drift	
Coul-SR:LIG-Protein	-434.158	11	63.326	-12.1007	(kJ/mol)
LJ-SR:LIG-Protein	-444.279	3.5	24.115	18.9245	(kJ/mol)
Total	-878.437	10	--	6.82384	(kJ/mol)

**Energy**	Average	Err.Est.	RMSD	Tot-Drift	
Coul-SR:LIG-Protein	-285.981	16	65.7979	-101.492	(kJ/mol)
LJ-SR:LIG-Protein	-435.435	5	25.8652	27.7592	(kJ/mol)
Total	-721.416	12	--	-73.733	(kJ/mol)

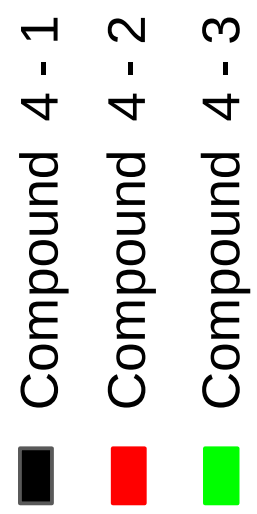

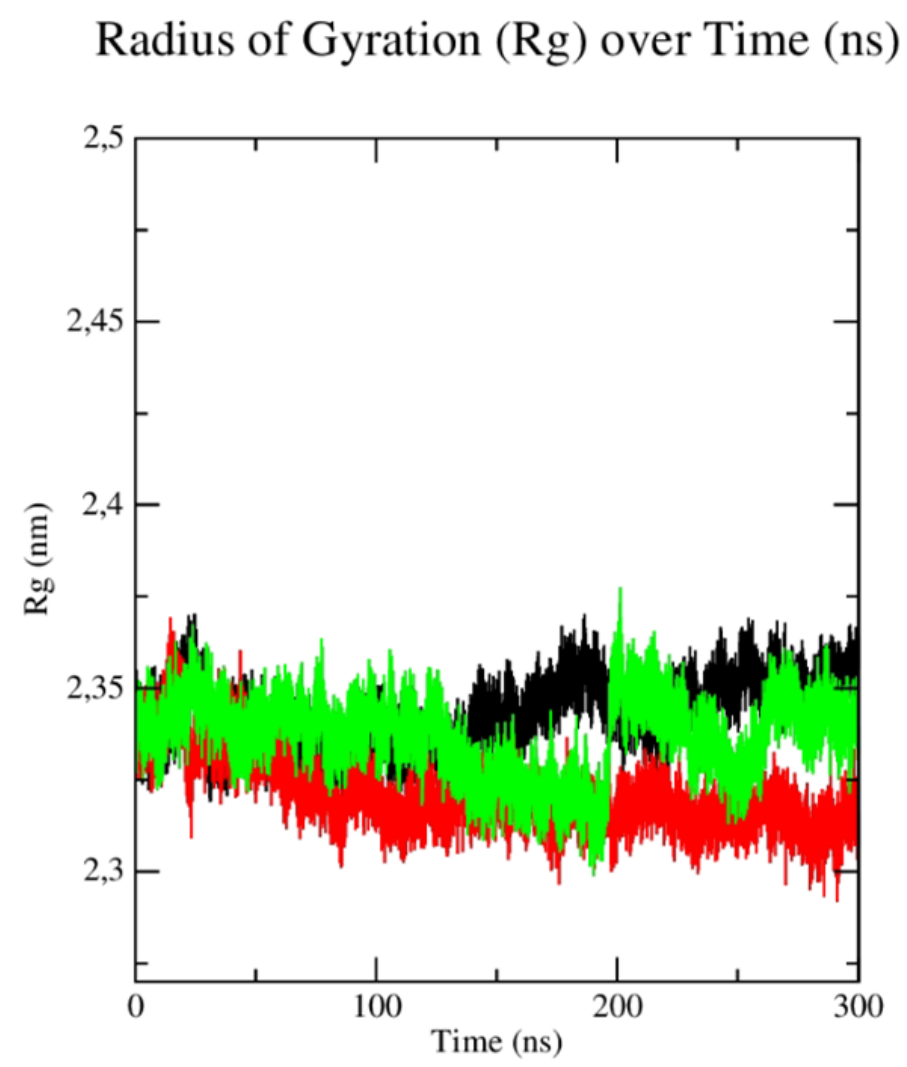

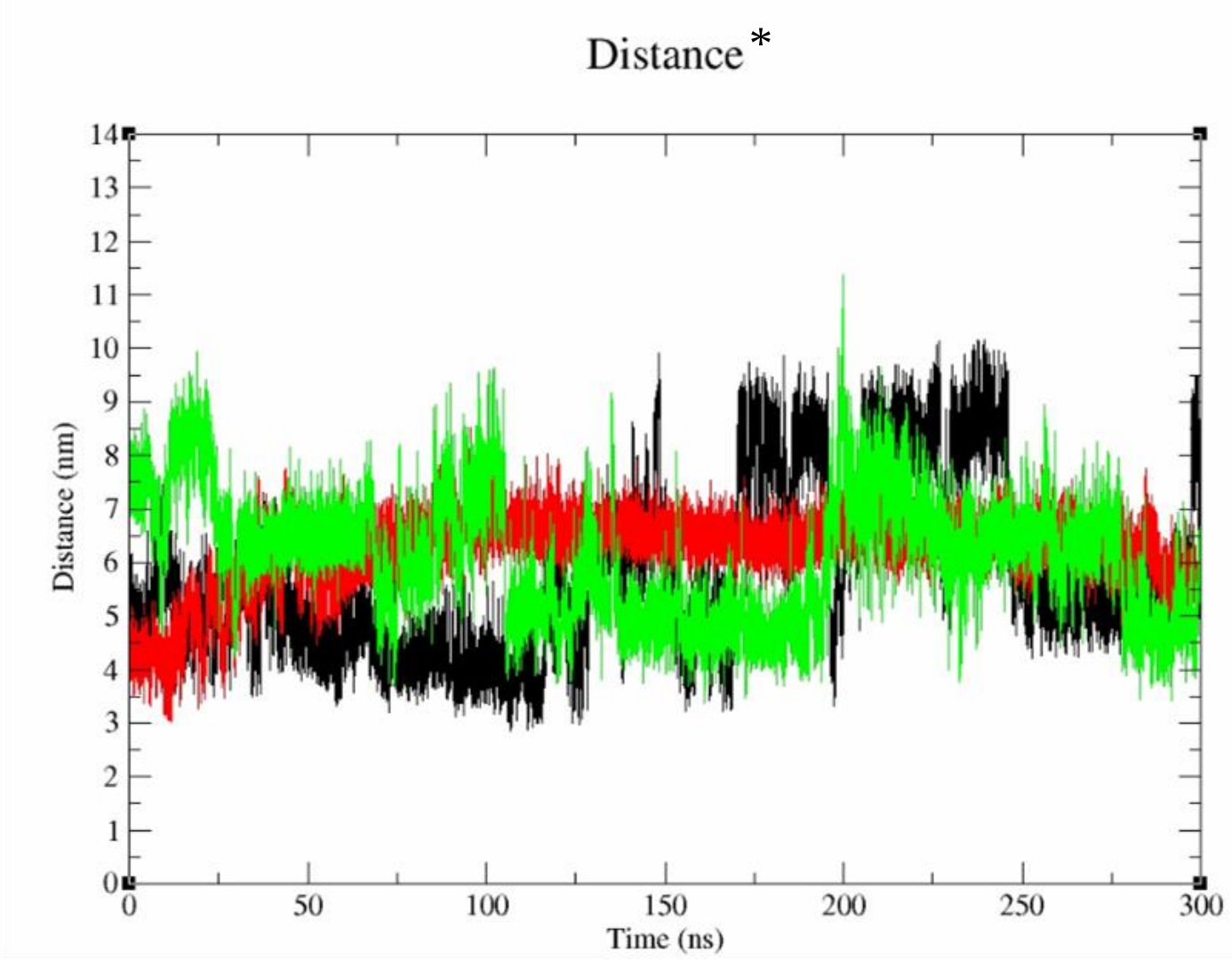

*this graph considers the distances between the closest carbonyl group of the compound to the hydroxyl group of the Ser221

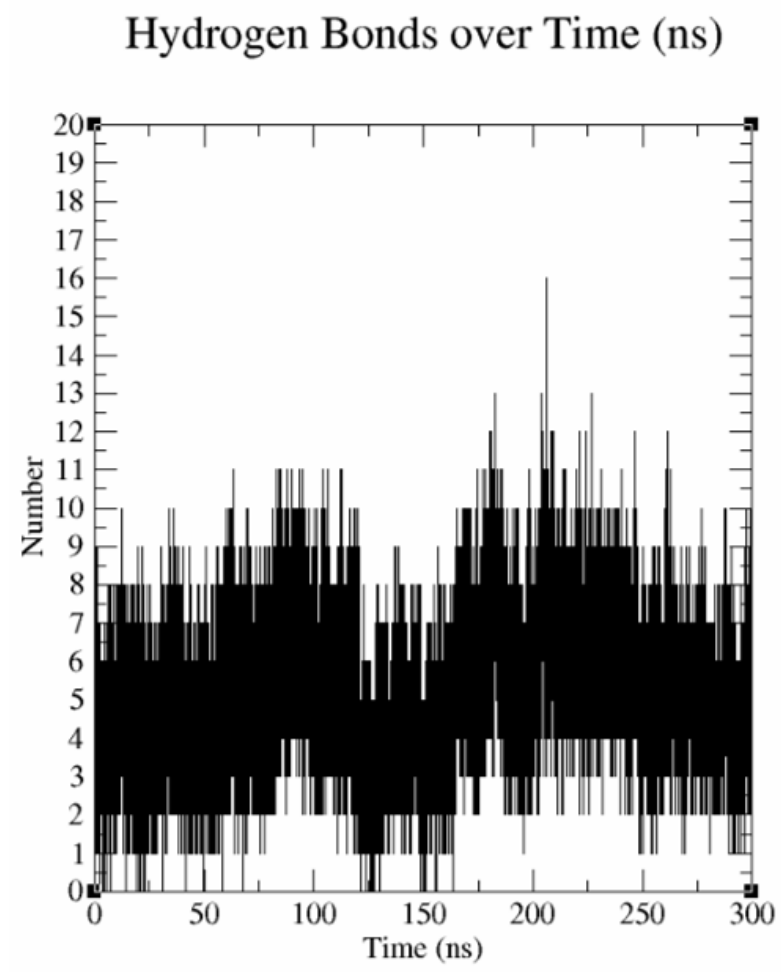

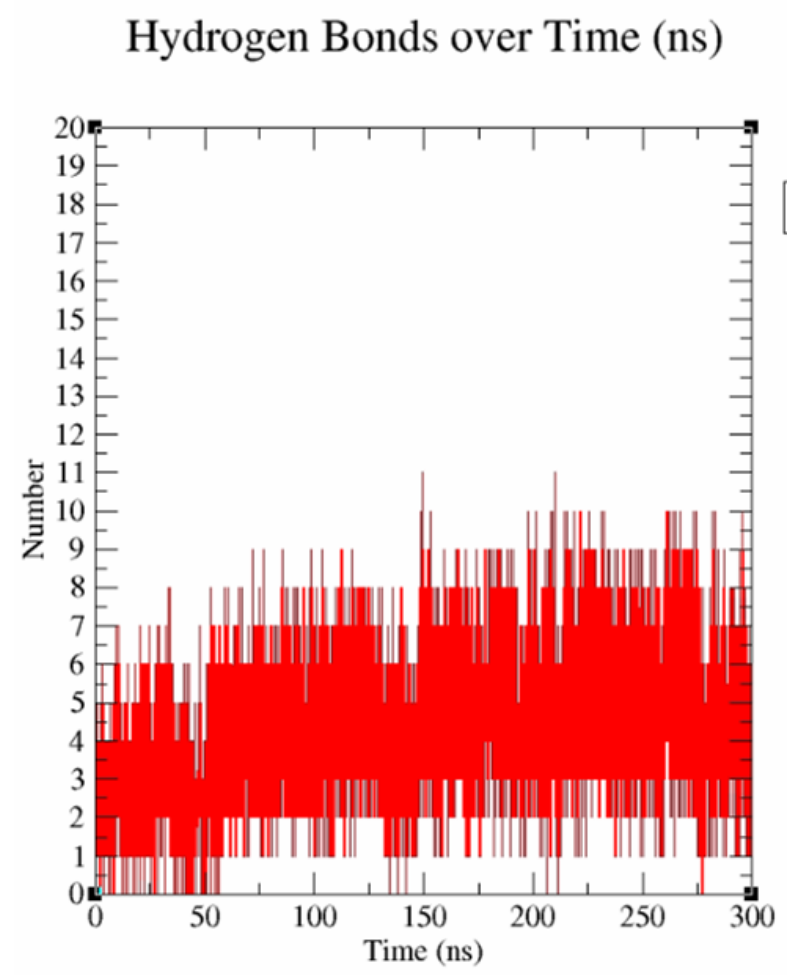

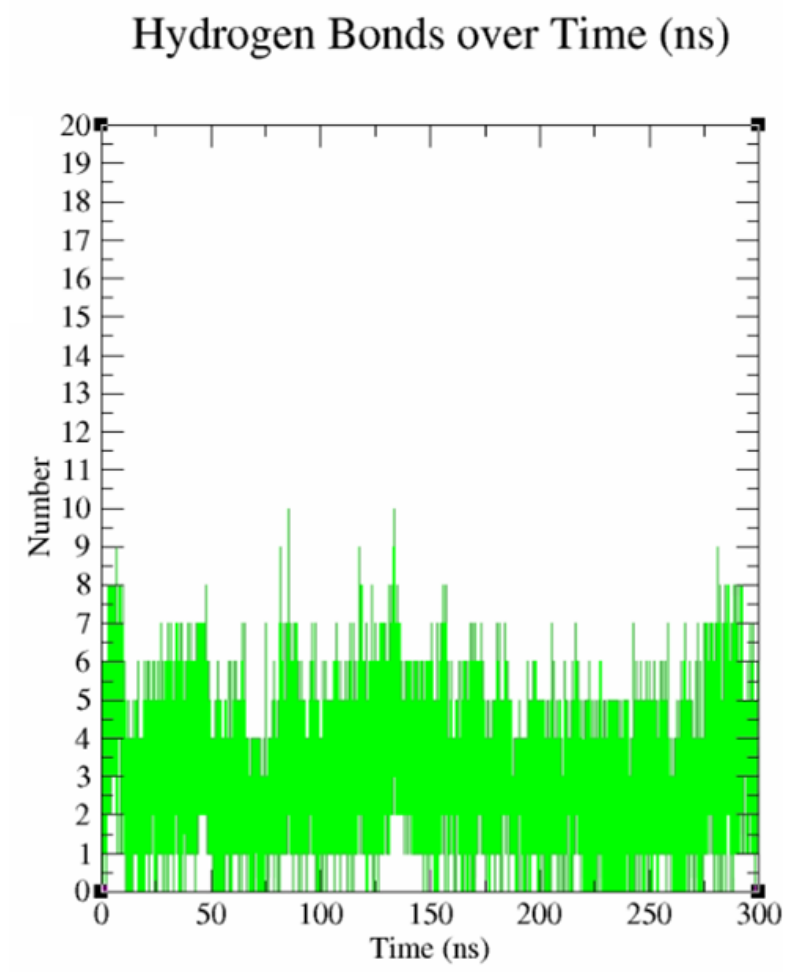

Figure S4. Molecular dynamic results for Compound 4.

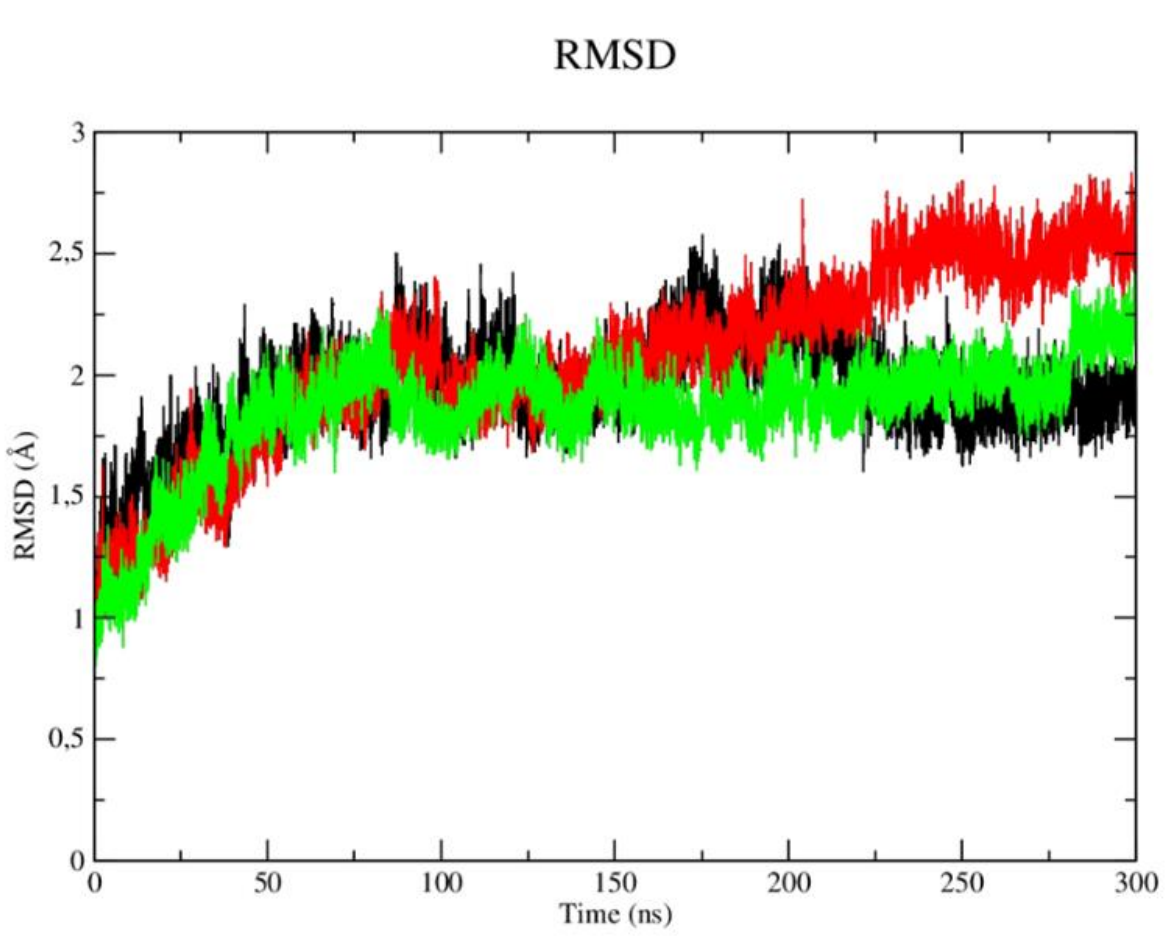

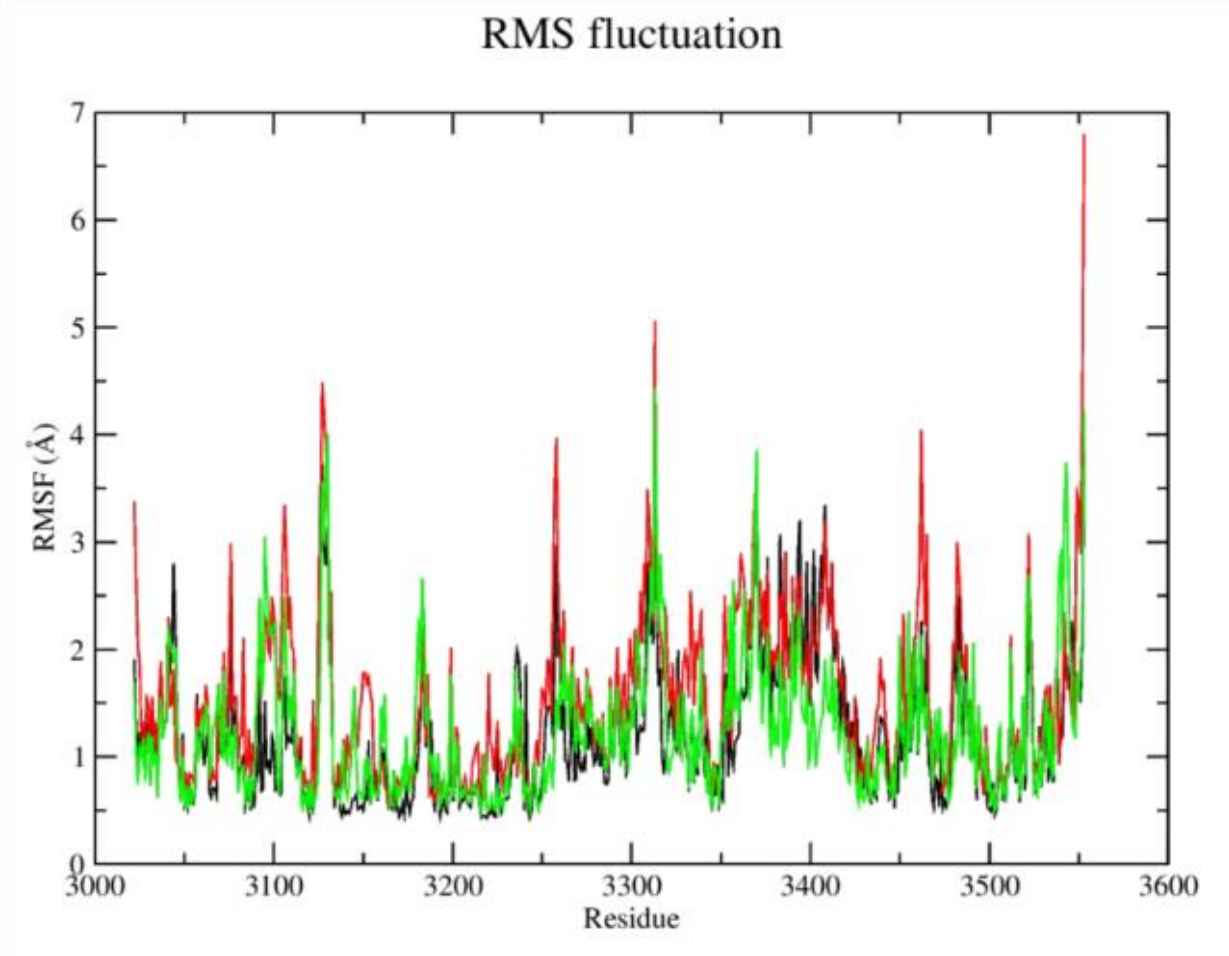

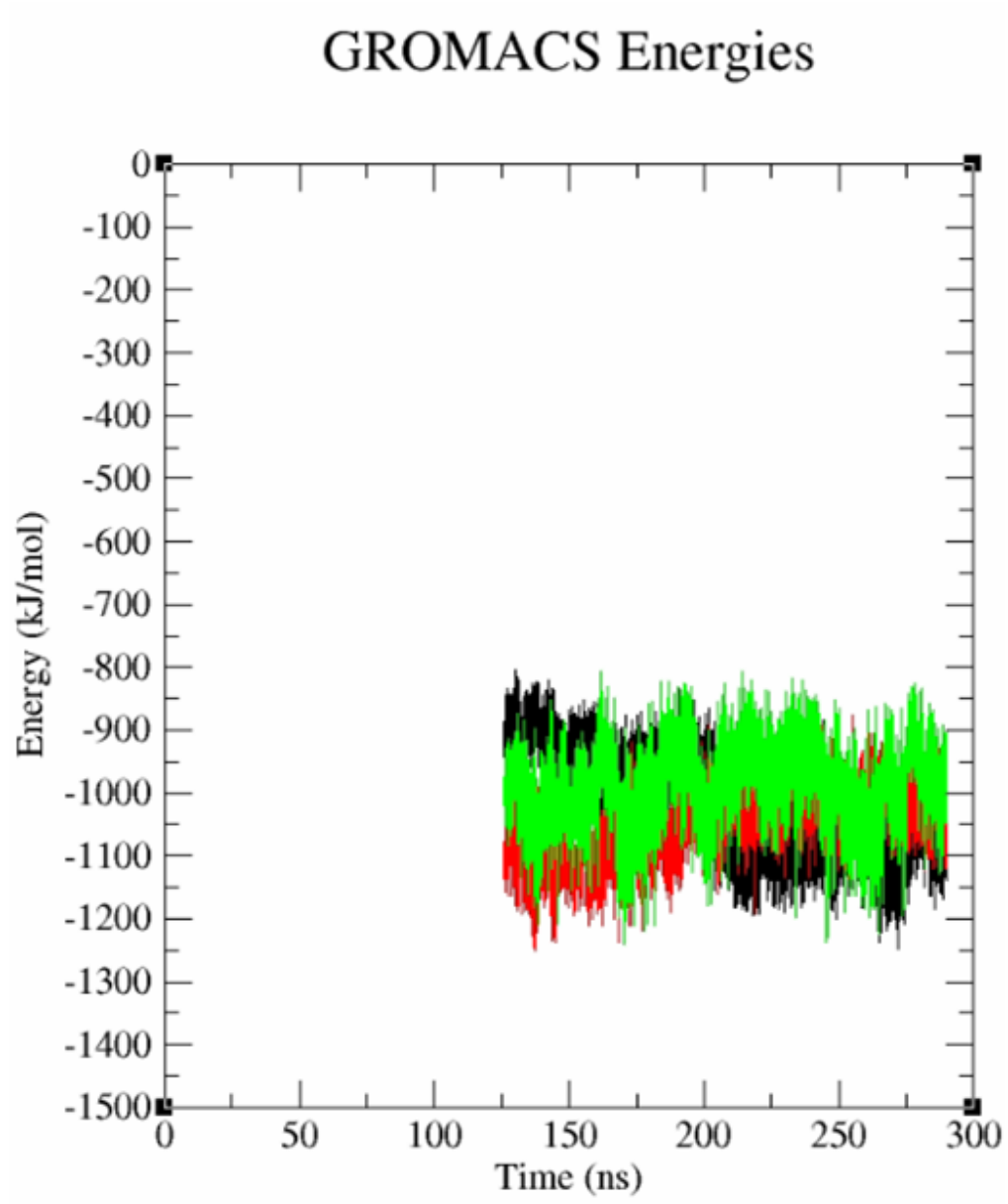

**Energy**	Average	Err.Est.	RMSD	Tot-Drift	
Coul-SR:LIG-Protein	-368.737	26	71.448	-154.349	(kJ/mol)
LJ-SR:LIG-Protein	-644.514	6	26.9004	-32.0884	(kJ/mol)
Total	-1013.25	31	--	-186.437	(kJ/mol)

**Energy**	Average	Err.Est.	RMSD	Tot-Drift	
Coul-SR:LIG-Protein	-372.293	15	51.1971	62.7657	(kJ/mol)
LJ-SR:LIG-Protein	-695.869	1.8	23.4682	6.83418	(kJ/mol)
Total	-1068.16	15	--	69.599	(kJ/mol)

**Energy**	Average	Err.Est.	RMSD	Tot-Drift	
Coul-SR:LIG-Protein	-416.162	16	65.3331	100.87	(kJ/mol)
LJ-SR:LIG-Protein	-582.799	5.6	27.9636	-33.7458	(kJ/mol)
Total	-998.96	11	--	67.1239	(kJ/mol)

Compound 5 - 1
Compound 5 - 2
Compound 5 - 3

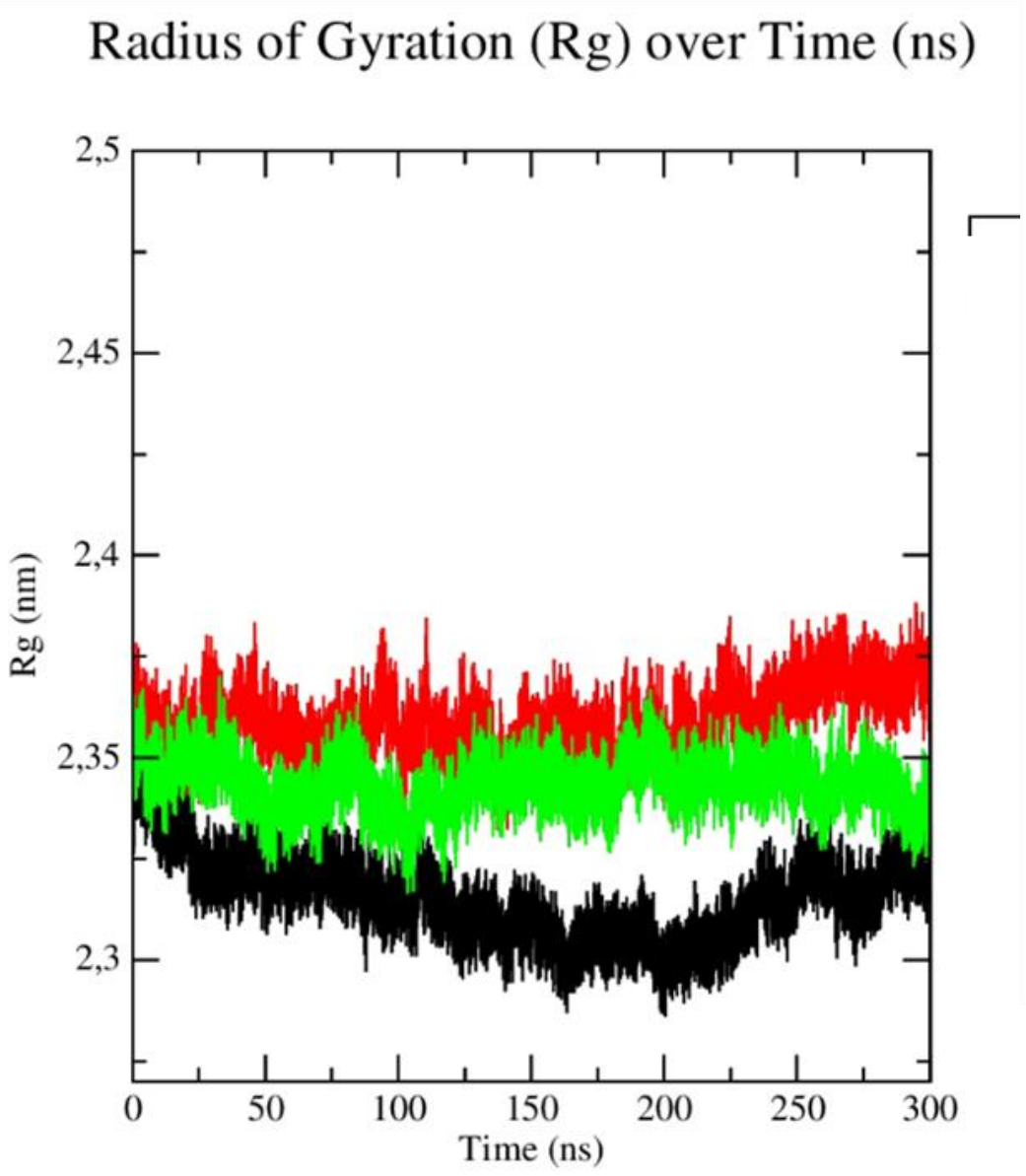

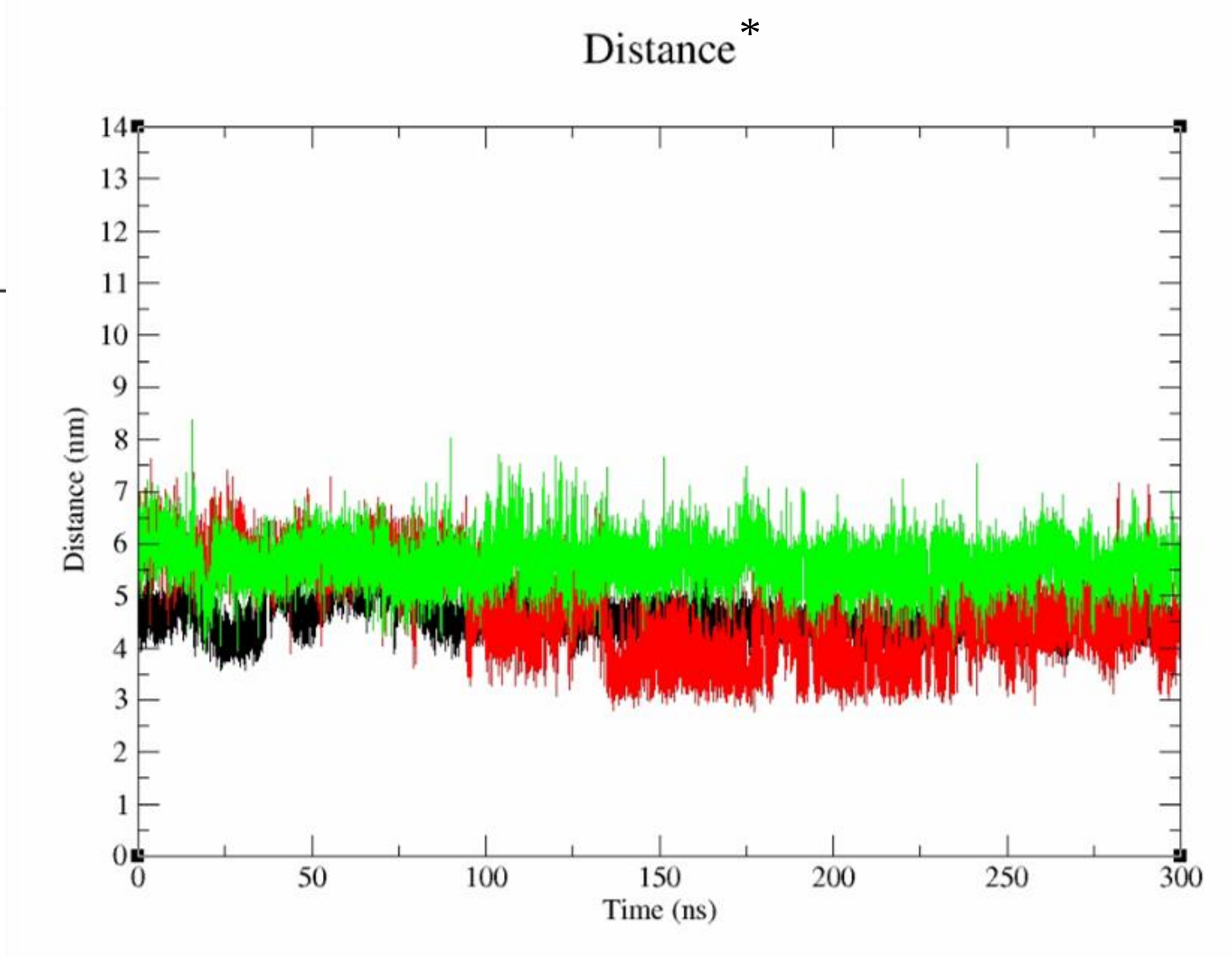

*this graph considers the distances between the closest carbonyl group of the compound to the hydroxyl group of the Ser221

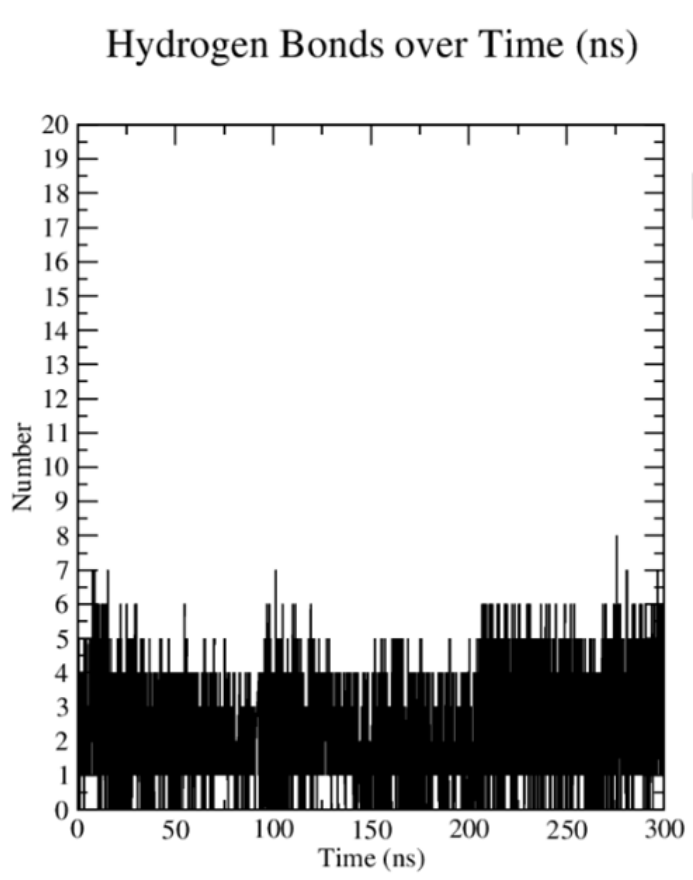

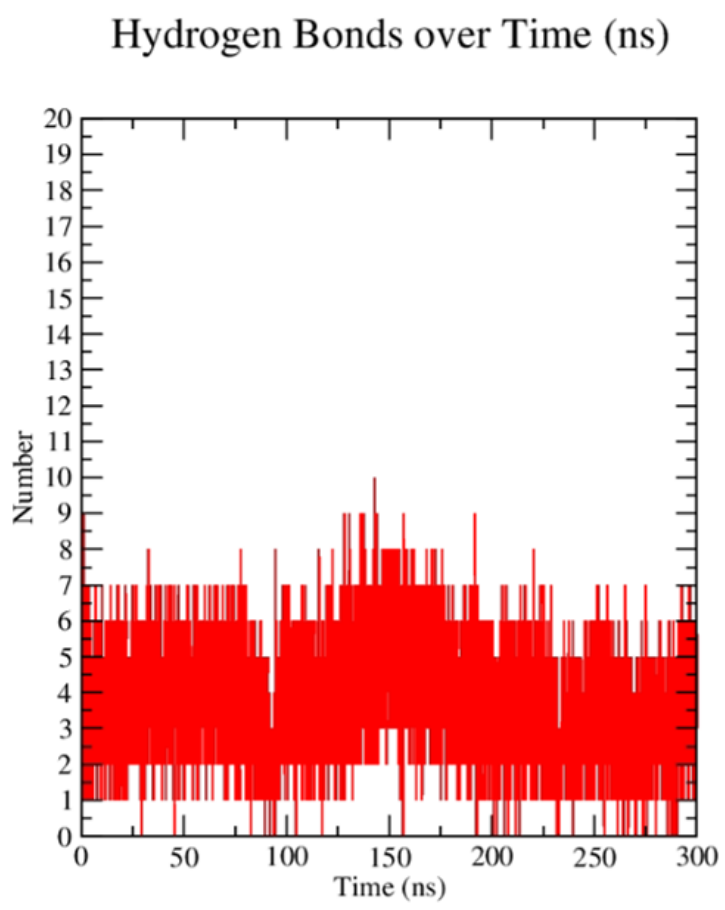

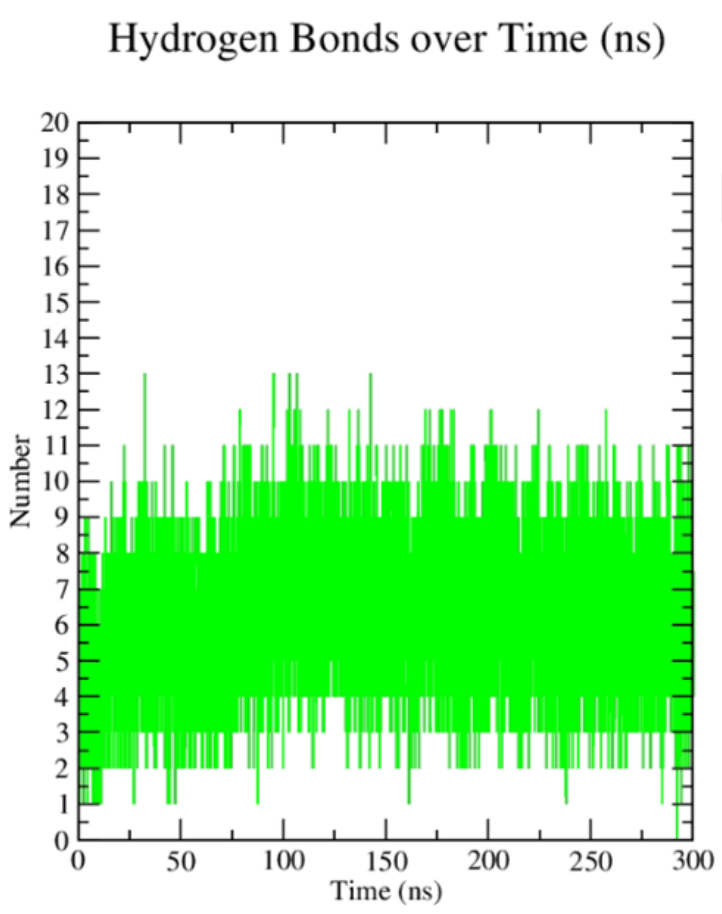

Figure S5. Molecular dynamic results for Compound 5.

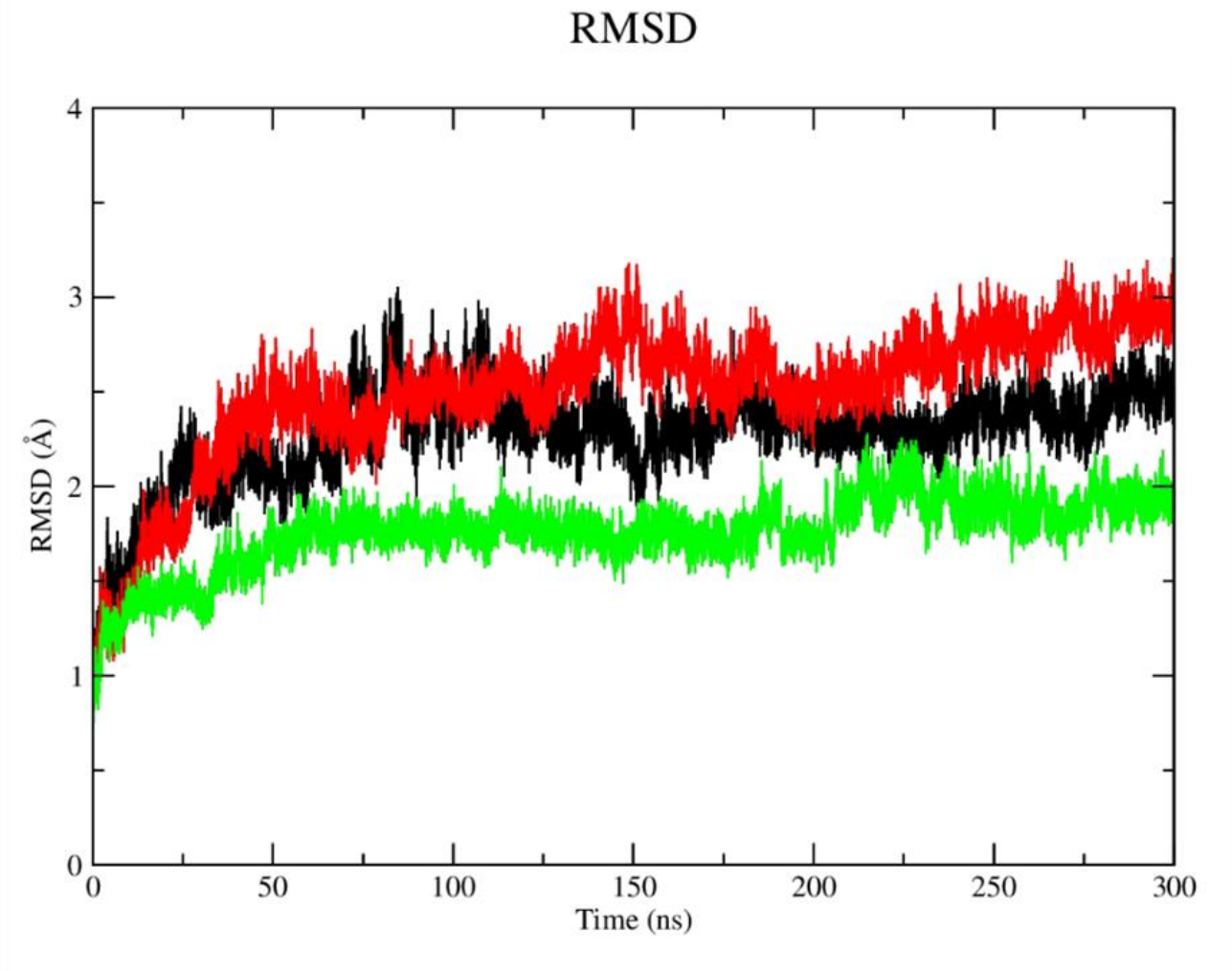

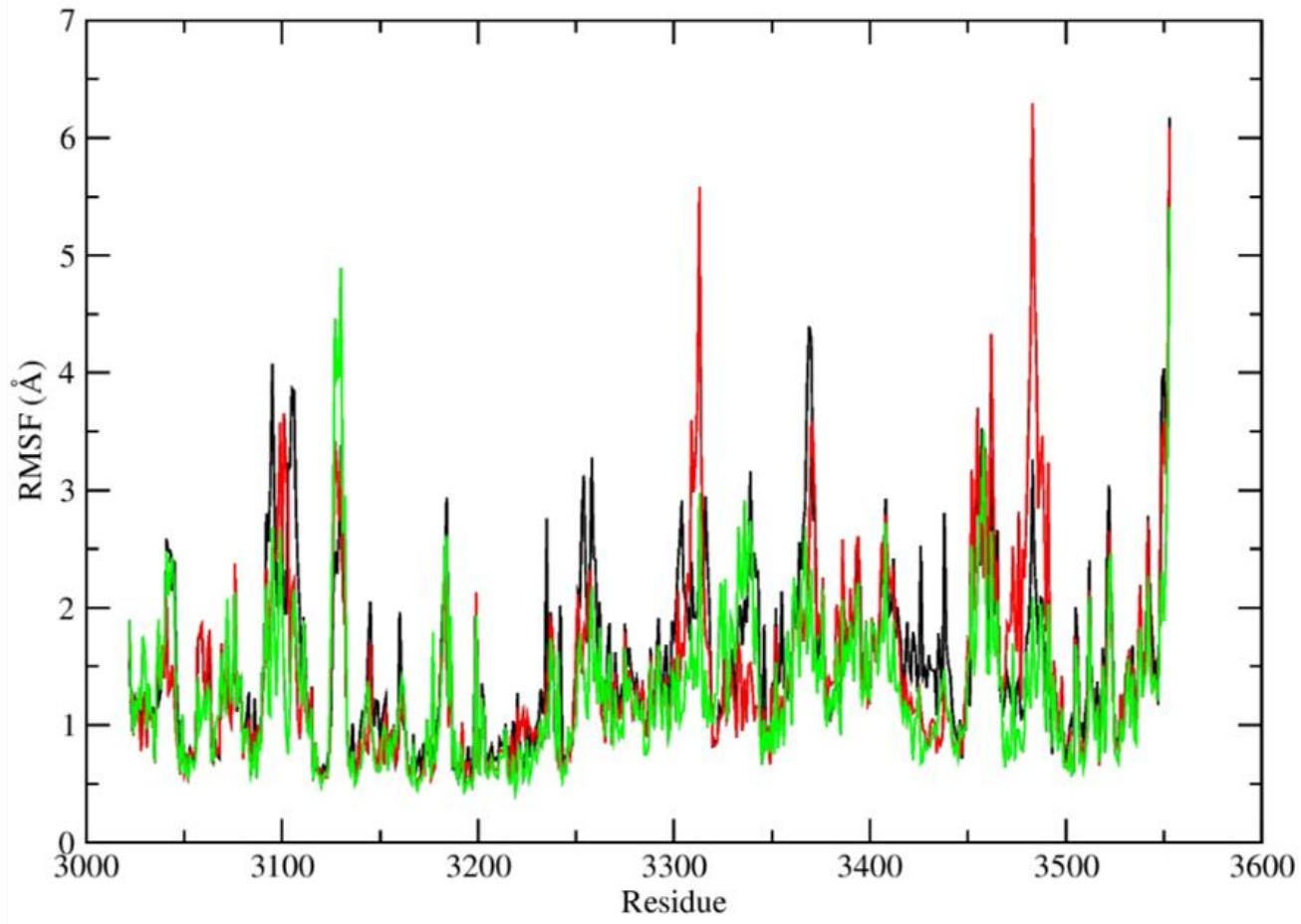

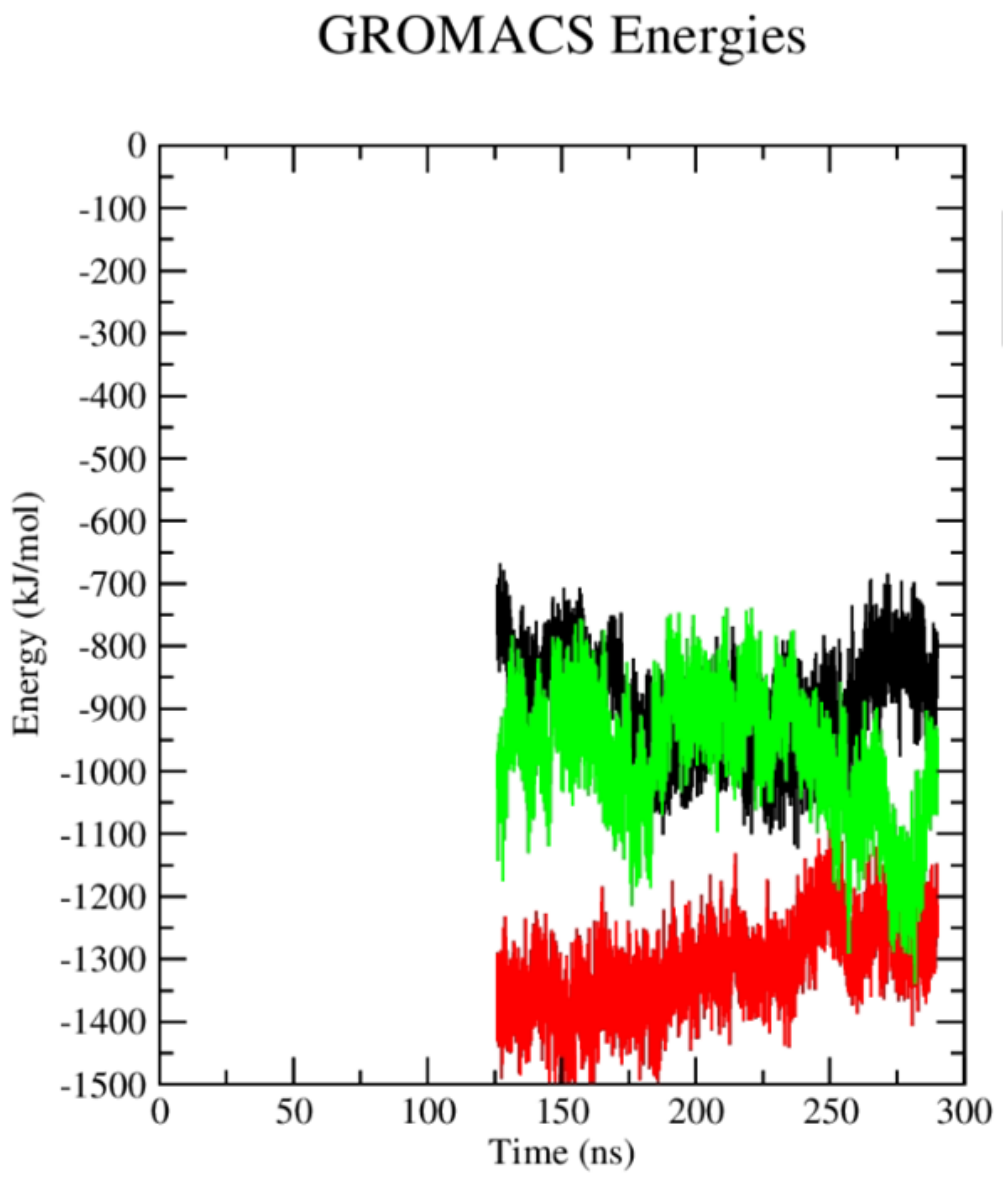

**Energy**	Average	Err.Est.	RMSD	Tot-Drift	
Coul-SR:LIG-Protein	-281.128	19	66.7249	1.73108	(kJ/mol)
LJ-SR:LIG-Protein	-612.148	16	42.1333	48.9786	(kJ/mol)
Total	-893.276	32	--	50.7097	(kJ/mol)

**Energy**	Average	Err.Est.	RMSD	Tot-Drift	
Coul-SR:LIG-Protein	-503.58	21	64.4098	109.259	(kJ/mol)
LJ-SR:LIG-Protein	-819.117	6.3	30.139	-15.5296	(kJ/mol)
Total	-1322.7	23	--	93.7296	(kJ/mol)

**Energy**	Average	Err.Est.	RMSD	Tot-Drift	
Coul-SR:LIG-Protein	-313.641	22	72.7188	-16.3053	(kJ/mol)
LJ-SR:LIG-Protein	-677.138	5.7	32.258	-1.16556	(kJ/mol)
Total	-990.779	27	--	-17.4709	(kJ/mol)

Compound 6 - 1
Compound 6 - 2
Compound 6 - 3

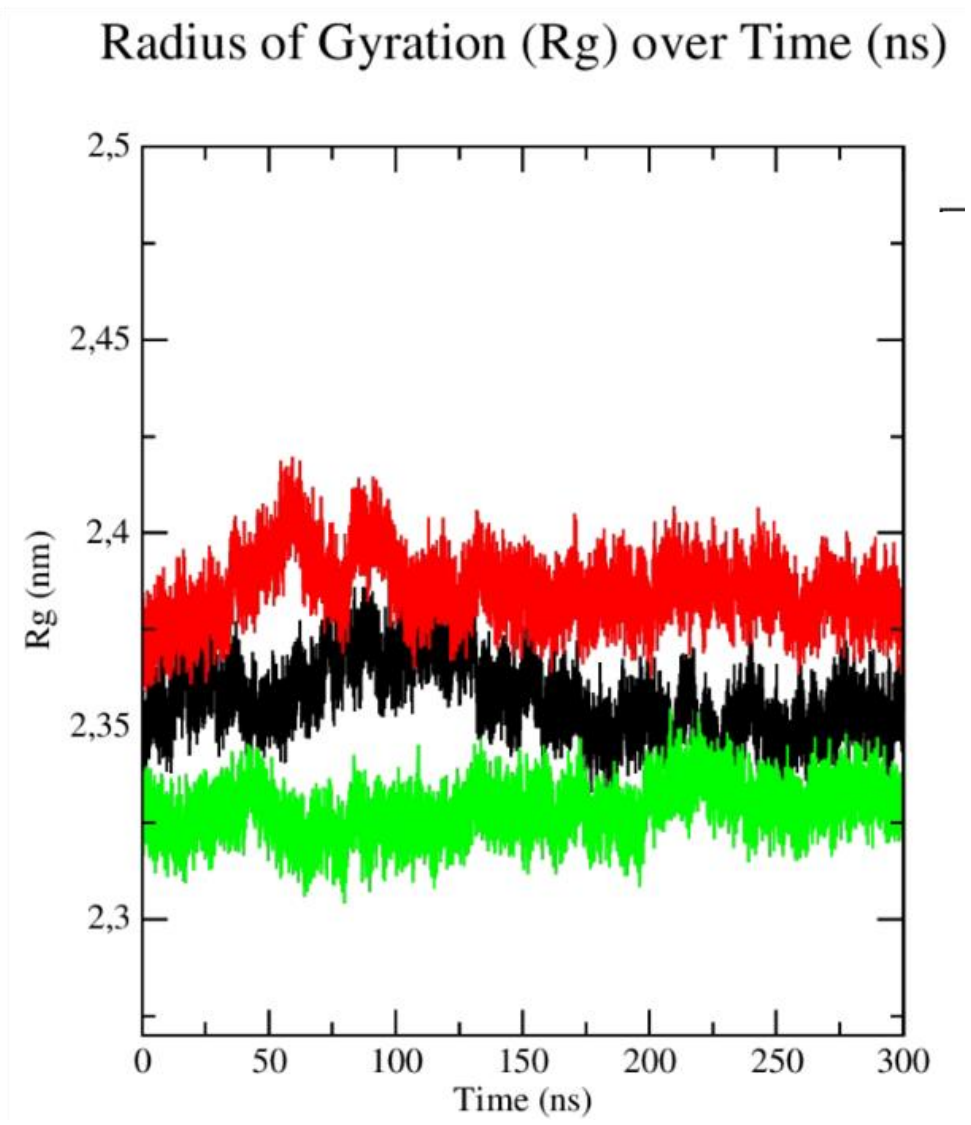

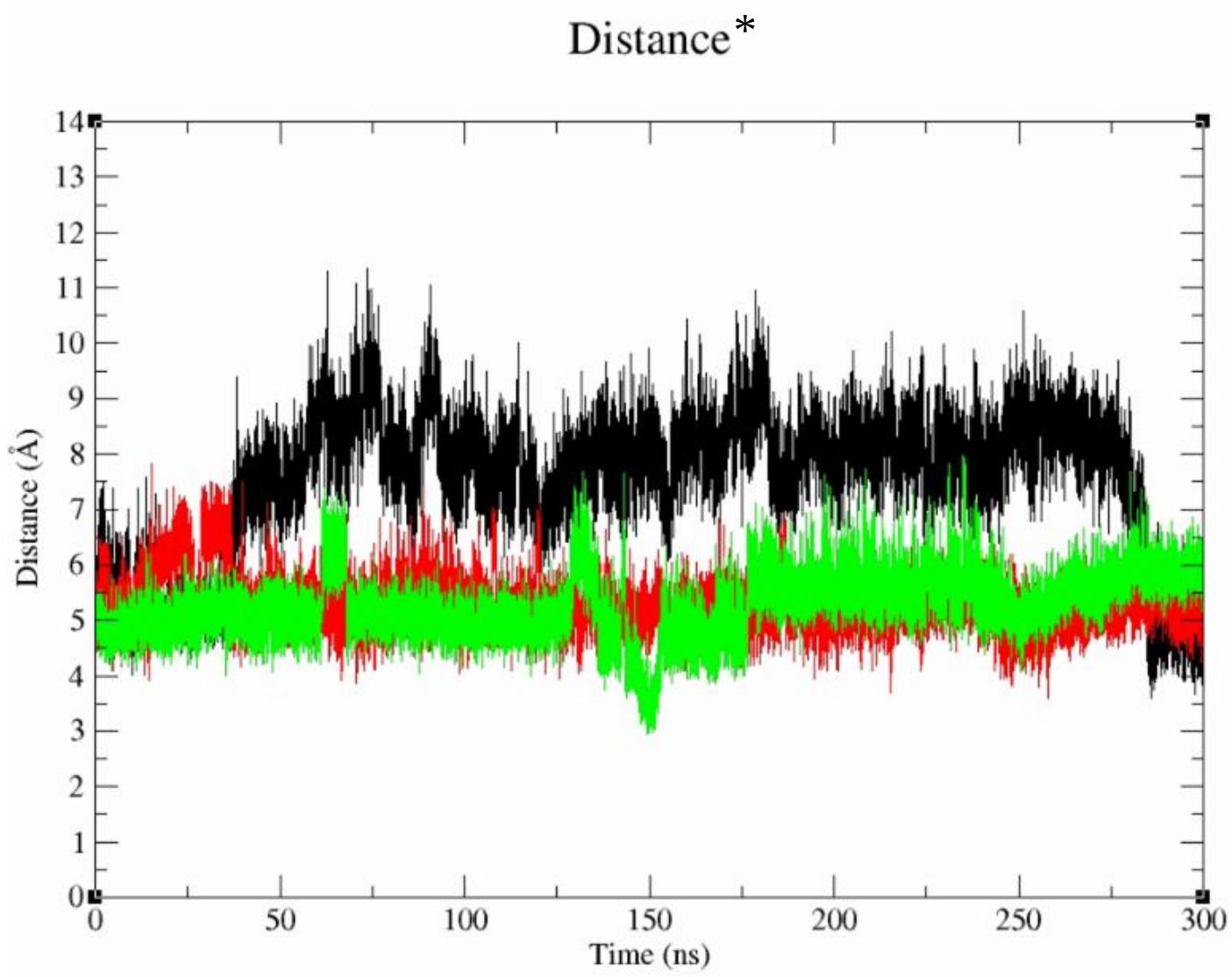

*this graph considers the distances between the closest carbonyl group of the compound to the hydroxyl group of the Ser221

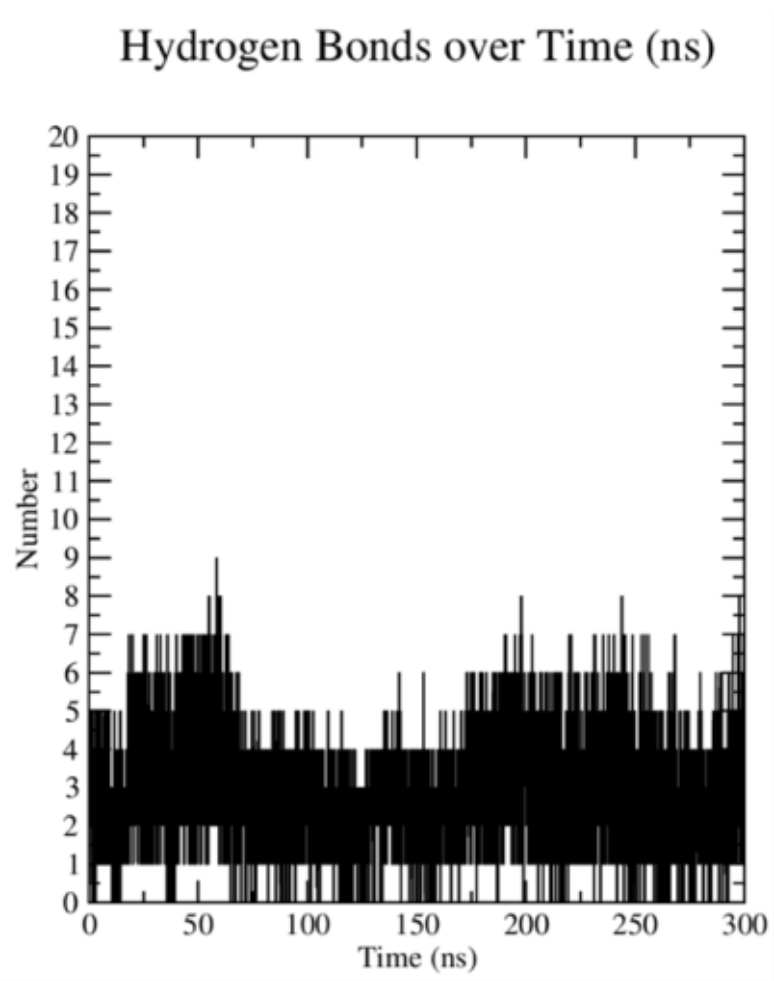

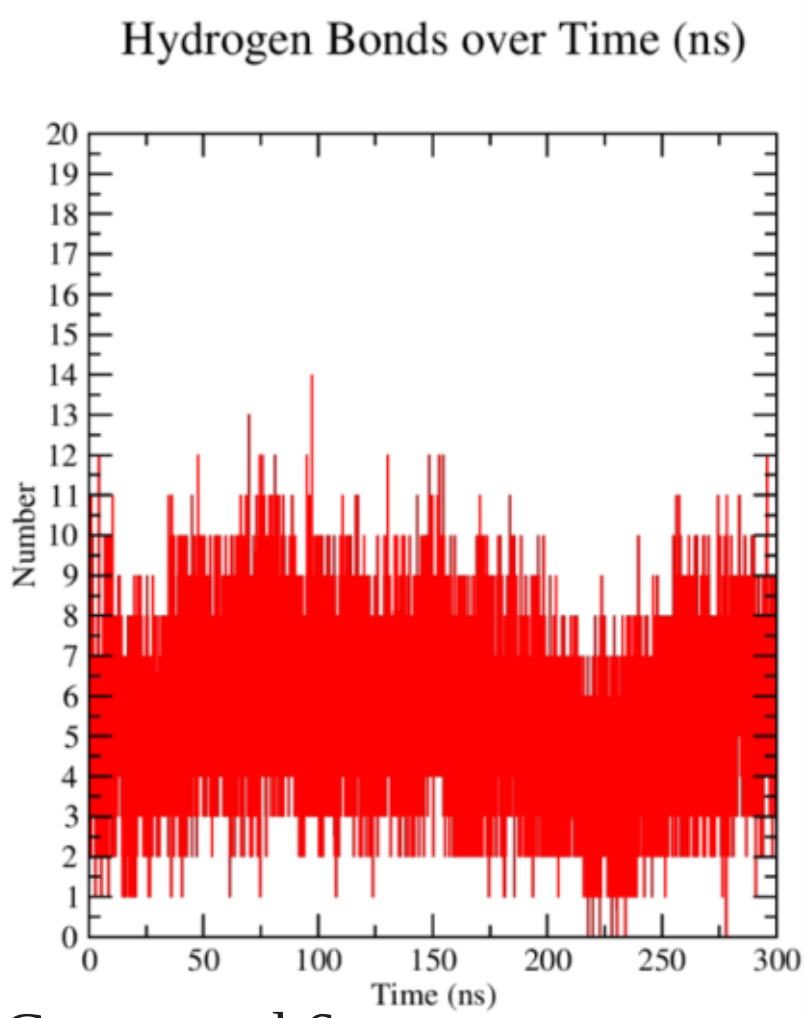

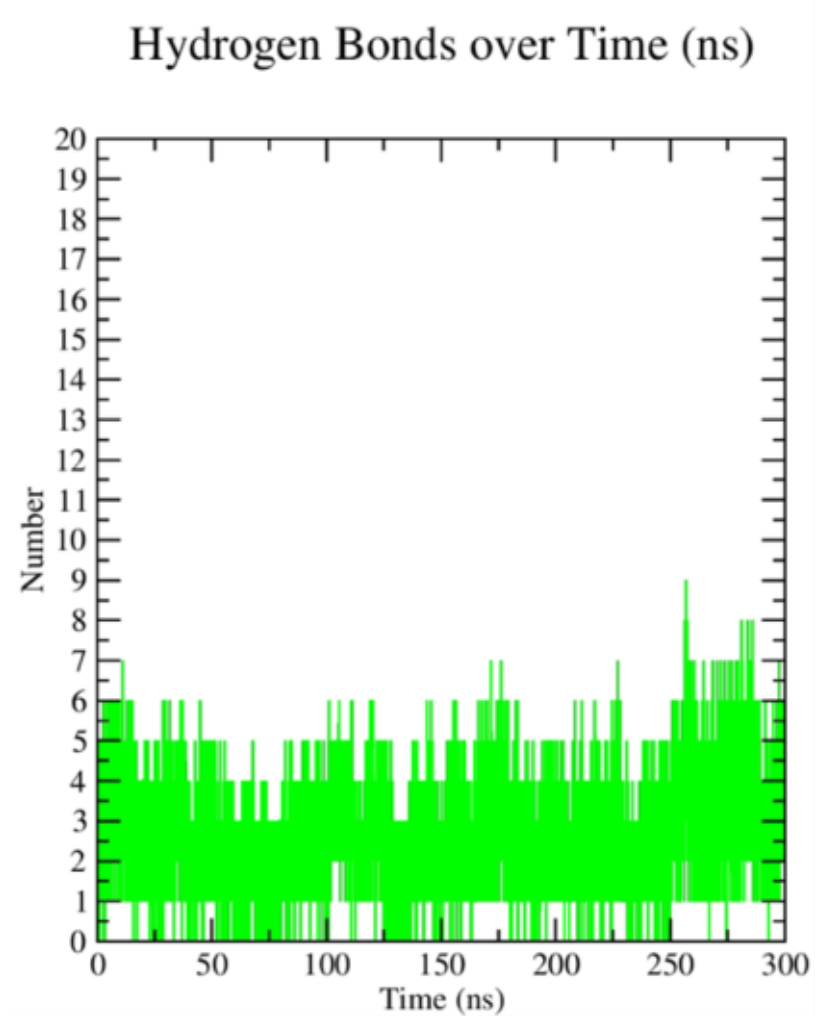

Figure S6. Molecular dynamic results for Compound 6.
